# Catalyst-free hydrophosphination of alkenes in presence of 2-methyltetrahydrofuran: a green and easy access to a wide range of tertiary phosphines†

**DOI:** 10.1039/c9ra04896k

**Published:** 2019-08-30

**Authors:** Damien Bissessar, Julien Egly, Thierry Achard, Pascal Steffanut, Stéphane Bellemin-Laponnaz

**Affiliations:** Institut de Physique et Chimie des Matériaux de Strasbourg (IPCMS), Université de Strasbourg, CNRS UMR 7504 23 Rue du Loess, BP 43 F-67034 Strasbourg Cedex 2 France bellemin@unistra.fr; CLARIANT Plastics and Coatings AG Rothausstrasse 61 4132 Muttenz Switzerland

## Abstract

A hydrophosphination reaction that is free of base, acid and catalyst, using only 2-methyltetrahydrofuran as additive has been performed. A new family of mono-, di-, tri- and tetra-phosphines compounds are obtained in good to excellent yields by adding diphenylphosphine to alkenes, mono- and polyfunctional acrylics (based on acrylate and methacrylate motifs) and acrylamide substrates. Addition of four equivalent of bio-mass derived 2-MeTHF into the reaction media improves both conversion and time of the reaction and reduces the sensitivity of the reactants over oxidation. This simple, straightforward and atom-economic method respects the principles of Green Chemistry. Furthermore, in each case this transformation shows an exclusive regioselectivity towards the anti-Markovnikov products.

## Abstract 2

A hydrophosphination reaction that is free of base, acid and catalyst, using only 2-methyltetrahydrofuran as additive has been performed.

## Graphical abstract

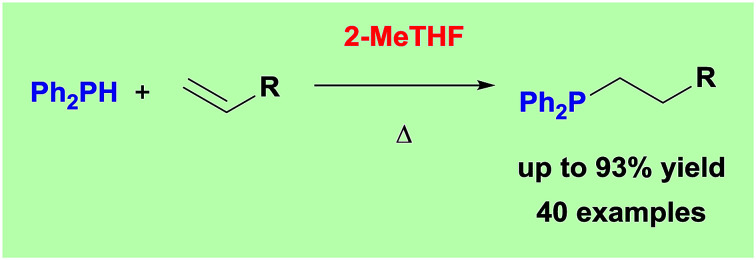

## Introduction

Phosphorus chemistry is prominent for its essential roles in the metabolism of phosphates in animals and plants. Phosphates are key components of genetic information.^1,2^ Though less known, phosphorus is a crucial element in organic compounds in everyday life. Phosphorus is used in a wide range of applications, from biology (fertilizers, antibiotics, cancer therapies,^3^ pesticides, *etc.*) to the plastic industry (stabilizers, flame retardant,^4^ antioxidants, *etc.*). Nowadays, many substances that contain phosphorus are produced on an industrial scale for the agrochemical, pharmaceutical and chemical industries to improve quality of life. Organophosphorus compounds also have a central role in research laboratories, particularly as ligands for coordination chemistry and for the development efficient homogeneous or heterogeneous catalysts (including asymmetric catalysts).^5^ In addition the development of phosphorus-based organocatalysts has become very popular over the past years.^6^

Tertiary phosphines PR_3_ play a pivotal role in the growing use of organophosphorus compounds. Their synthetic accesses often require the use of metal phosphides or organometallic reagents thus generating quantities of unwanted wastes. Synthetic chemists, both in industry and academia, are increasingly concerned about the environmental problems that can be associated with such syntheses. Thus, synthetic pathways that involve the use of environmentally friendly solvents and do not produce by-products are desirable.

The direct hydrophosphination of unsaturated substrates such as alkenes or alkynes offers great benefits in terms of atom economy.^7,8^ It allows the direct formation of a P–C bond with no by-product formation.^9^ This reaction is currently receiving considerable attention and the recent literature shows an increasing number of metal catalysts^10^ (transition metals and lanthanides) for that transformation,^11^ the main purpose being to bring up regioselectivity and/or enantioselectivity or stereoselectivity.^12–16^ However, the hydrophosphination reaction does not essentially require the presence of a metal catalyst and can be conducted through the homolytic cleavage of the PH bond or through the heterolytic cleavage of the PH bond mediated by an acid or a base.^17^ Some recent reports have revealed that the hydrophosphination reaction could be conducted under neat^18^ conditions to afford selectively the anti-Markovnikov product.^19^ However, neat conditions may have drawbacks that need to be overcome.^20^ That is for example homogeneity or viscosity issues, or possible acceleration of side reactions. In addition, primary and secondary phosphines (RPH_2_ or R_2_PH) are often very sensitive to air and dilution in a proper solvent allows easy handling of the reagents.

**Scheme 1:**
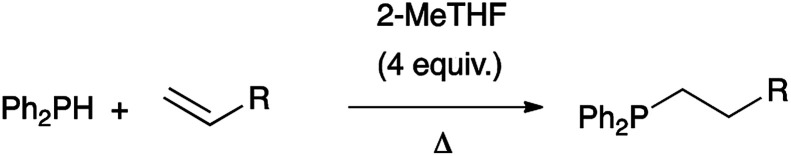
Hydrophosphination of alkene.

Herein we show that 2-methyltetrahydrofuran (Me-THF), a biomass-derived substitute of THF,^21^ is the solvent of choice for the catalyst-free hydrophosphination of alkenes (Scheme 1). It allows improved conversion of the reaction while keeping high regioselectivity. The manipulation of the compounds was also facilitated because Me-THF reduces the sensitivity of the products toward air and moisture. Overall, better yields under smoother conditions and shorter reaction time were achieved.

We demonstrated the versatility of this method on a wide range of substrates. For example, we used acrylic monomers as substrates to get access to a very large range of tertiary phosphines in a very straightforward manner. Mono-, di-, tri- and tetra-phosphines were obtained in good to excellent yields.

## Results and discussion

Owing to our current interest in the development of tertiary phosphines for coordination chemistry, we investigated the hydrophosphination of alkenes using diphenylphosphine as reagent and in absence of catalyst. We studied the addition of diphenylphosphine to methyl acrylate with various solvents under argon in a closed vessel at 80 °C. In dichloromethane, acetonitrile, toluene or ethanol, no formation (or only traces) of the product 1 was observed (entries 1–4, Table 1). However, using 2-methyltetrahydrofuran, the product was then formed with 5% conversion. 2-Methyltetrahydrofuran offers many advantages with regards to the principles of Green Chemistry.^21–23^ It can be produced from renewable resources and may be easy to degrade. Moreover, its higher boiling point compared to THF allows reactions to be carried out at higher temperatures. We thus decided to investigate the reaction at higher concentration and found that the reaction went to completion, giving the expected product 1 in quantitative yield with only 4 equivalents of 2-MeTHF with respect to the phosphine. As recently noticed by Alonso and collaborators, the reaction may be conducted under neat condition and indeed the product was also obtained in quantitative yield without solvent (entry 6).^18^ Nevertheless, the addition of 2-MeTHF to the reaction mixture was beneficial in term of activity but also practically, since the sensitivity of the reactants towards oxygen and moisture was reduced by the presence of 2-MeTHF.

**Table 1:** Addition of diphenylphosphine to methyl acrylate: solvent effect^a^

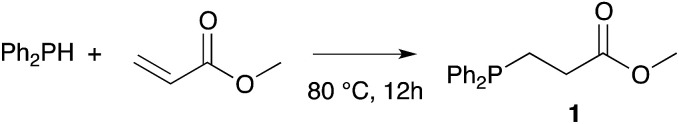		
Entry	Solvent	Conversion (%)
1	CH_2_Cl_2_	0
2	CH_3_CN	Trace
3	Toluene	Trace
4	EtOH	0
5	2-MeTHF	5
6	Neat	98
7^b^	2-MeTHF (4 equiv.)	>99

a Reaction condition: methyl acrylate (1.16 mmol), diphenylphosphine (1.16 mmol), solvent 2.5 mL.

b Solvent 0.45 mL of 2-MeTHF (4 equiv.).

Table 2 shows the advantageous of the use of 2-MeTHF in the hydrophosphination of methyl acrylate when compared with reported catalytic procedures. The table displays three examples from the literature using iron or zirconium-based catalysts at room temperature (entries 1–3).^24–26^ When the reaction was carried out without solvent, we could isolate product 1 with 75% yield. Interestingly, the presence of 2-MeTHF improved the yield and methyl 3-(diphenylphosphine)propanoate was then isolated in 83% yield. The same trend was observed with styrene as substrate (85% *vs.* 80%) or methyl vinyl ketone as substrate (88% *vs.* 70%).

**Table 2:** Addition of diphenylphosphine to methyl acrylate^a^

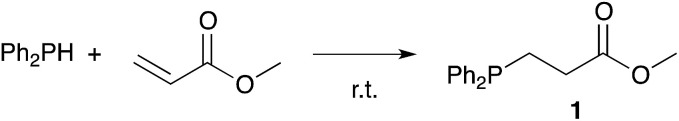					
Entry	Cat. (mol%)	Solvent	Time (h)	Yield (%)	Ref.
1	[CpFe(CO)_2_]_2_O (5)	Neat	6	96	23
2	[Salen-Fe]_2_O (1)	CH_3_CN	24	69	24
3^b^	LZrBn_3_ (10)	Neat	12	85	25
4	None	Neat	12	75	This work
5^c^	None	2-MeTHF	12	83	This work

a Reaction condition: methyl acrylate (1 equiv.), diphenylphosphine (1 equiv.).

b L = amine-bridged bis(phenolate) ligand.

c 2-MeTHF (4 equiv.).

**Fig. 1:**
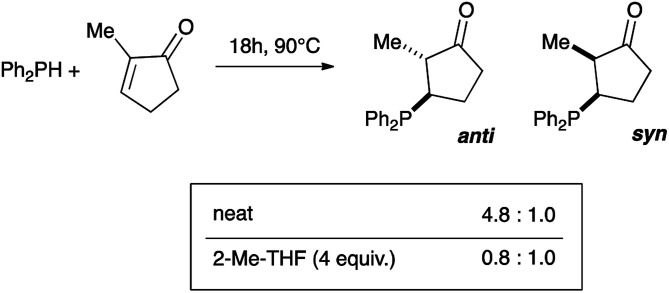
Catalyst-free hydrophosphination of 2-methyl-2-cyclopentenone and the corresponding *anti*/*syn* ratio product under neat conditions or in the presence of 2-MeTHF.

The mechanism of the hydrophosphination reaction has been studied, in particular by Koenig and collaborators and more recently by Alonso and collaborators.^18,27^ These investigations are in line with a duality of a radical mechanism and ionic mechanism and the experimental conditions are decisive for favouring one mechanism over the other. Finally, we conducted the hydrophosphination of 2-methyl-2-cyclopentenone using our protocol (Fig. 1). The reaction with diphenylphosphine at 90 °C for 18 hours gave the expected product with high conversion both under neat condition or in the presence of 4 equiv. of 2-MeTHF (>90%). Analysis of the crude by NMR and GC-MS established that the *anti*/*syn* ratio was 4.8 to 1.0 under neat condition whereas a ratio of 0.8 to 1.0 was observed when 2-MeTHF was used.^28^ Therefore under neat conditions, the *anti* product is mostly obtained *via* a *syn*-addition mechanism, in which the P–H bond is most likely added through a 4-membered transition state. In the presence of 2-MeTHF, both stereoisomers are obtained in the same proportion. This suggests that an ionic pathway is involved with an enol as intermediate, as in the case of Michael addition reactions.^29^ This experiment clearly demonstrates that the mechanism of the catalyst-free hydrophosphination is strongly dependent on the experimental conditions.

We then extended the procedure to a variety of alkene substrates, in particular styrene substrates. Table 3 shows results with several styrene-based compounds along with some other substrates. As expected, presence of 2-MeTHF led to higher yields under milder conditions. For example, the hydrophosphination of the styrene gave product 2 in 85% yield. 4-Bromostyrene and 4-vinylanisole were also converted into the expected product 3 and 4 with good yields (81% and 91%, respectively). The reaction with the methyl vinyl ketone gave the tertiary phosphine 7 with 88% yield after only one hour at room temperature. As example, the hydrophosphination of methyl vinyl ketone under neat condition gave the product in only 70% yield after 7 hours reaction at room temperature.^18^ Lower yields were obtained for the reaction with vinyloxy benzene or 2-vinyl phenol (5 and 8). Hydrophosphination of divinyl substrates is also possible: the reaction of divinylbenzene or 1,3-divinyltetramethyldisiloxane gave the corresponding diphosphines 6 and 9 with yields at respectively 85% and 78%.

**Table 3:** Catalyst-free hydrophosphination of alkenes in the presence of 4 equiv. of 2-MeTHF^a^

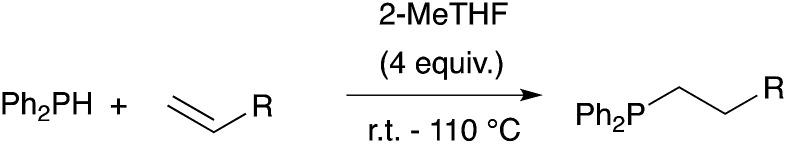
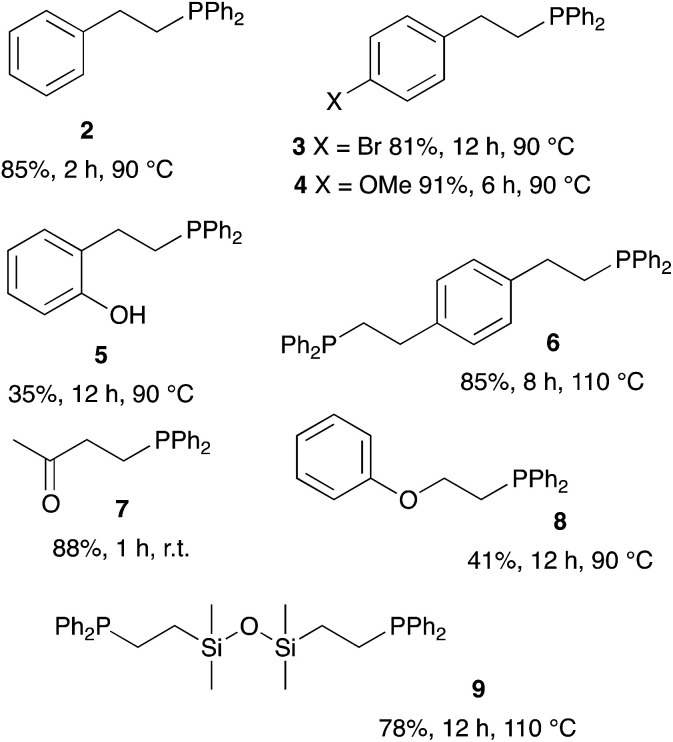

a Reaction condition: alkene (1 equiv.), diphenylphosphine (1.2 equiv.), 2-MeTHF (4 equiv.) under argon.

Acrylic resins exhibit many valuable properties (rigidity or flexibility, ionic or nonionic, hydrophobic or hydrophilic, durability and weatherability, *etc.*) that make them ideal for a large number of applications (coatings and adhesives, automotive finishes, paper/fiber processing, medical devices, *etc.*).^30,31^ Thus, global demand of acrylic based materials encouraged research laboratories and industries to develop new acrylic-containing monomers. Consequently, the range of cheap and readily accessible monomers continues to grow. We took advantage of this diversity to get access of a large range of tertiary phosphines. Diphenylphosphine readily react with commercially available monoacrylic ester such as ethyl, *n*-butyl, *t*-butyl or 2-ethylhexyl acrylate giving the corresponding tertiary phosphine in 81–95% yield, as shown on Table 4 (compounds 10–13). The hydrophosphination of PEG-acrylate gave the phosphine 15 which was found soluble in water and may be useful for application in homogeneous catalysis in aqueous solvent.^32^ Interestingly, it is also possible to react diphenylphosphine with acrylates that have been functionalized with a terminal alkyne or an unactivated alkene: the two products 14 and 16 were obtained with yields at 93% and 92% respectively. We could also introduce interesting moieties such as thiophene (17) or trialkoxy chains for further development of materials with liquid crystal properties (18).

**Table 4:** Catalyst-free hydrophosphination of monoacrylate in the presence of 4 equiv. of 2-MeTHF^a^

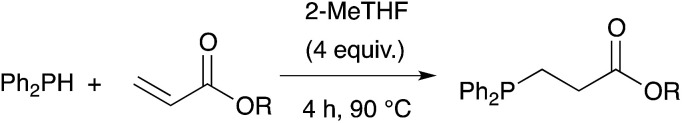
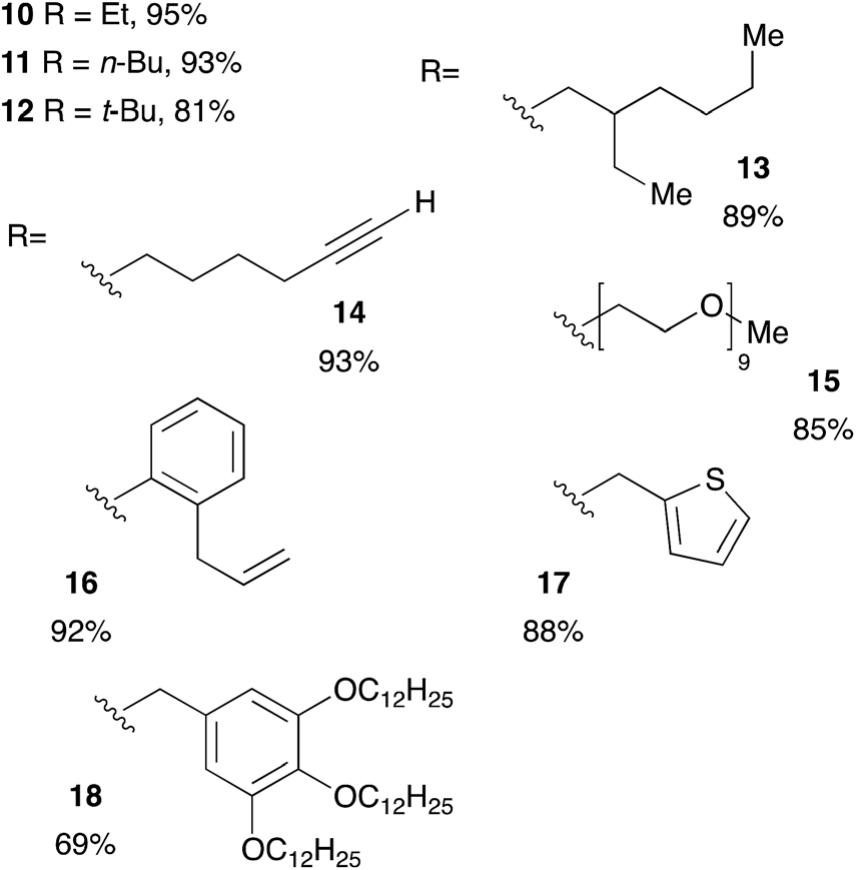

a Reaction condition: alkene (1 equiv.), diphenylphosphine (1.2 equiv.), 2-MeTHF (4 equiv.) under argon.

Methacrylate derivatives are interesting substrates that are readily accessible and cheap. We investigated the hydrophosphination reaction with various methacrylates as shown on Table 5. Good results were obtained with commercially available substrates such as 19, 20, 21 (73%, 76% and 56% yield, respectively) but this is somewhat lower than the corresponding acrylate counterparts 10, 11, 12 (95%, 93% and 81% yield, respectively). The methyl group located in α position of the alkenes may explain the lower reactivity of this family of substrates. Nevertheless, compound 22 that also contains a secondary amine function could be isolated in 89% yield. We also tested the itaconic acid methyl ester and obtained the expected product 25 in 81% yield.

**Table 5:** Catalyst-free hydrophosphination of monomethacrylate derivatives and the methyl ester itaconique acid in the presence of 4 equiv. of 2-MeTHF^a^

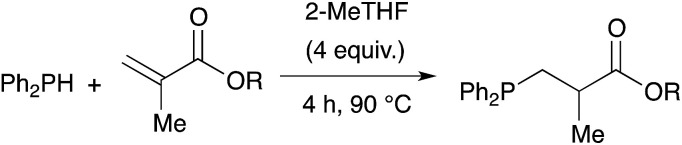
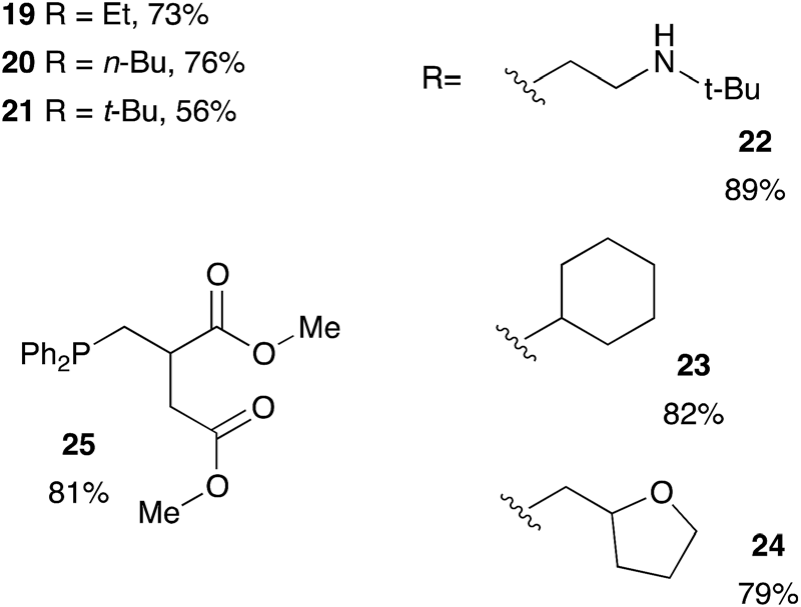

a Reaction condition: alkene (1 equiv.), diphenylphosphine (1.2 equiv.), 2-MeTHF (4 equiv.) under argon.

**Fig. 2:**
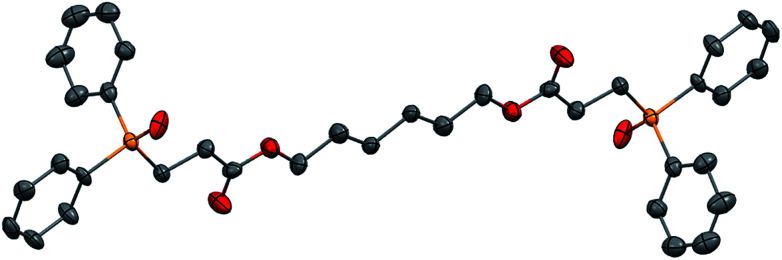
ORTEP representation of phosphine oxide of 28, with thermal ellipsoids drawn at the 50% probability level.

We further extended our investigations with polyfunctional acrylics which are widely available on the market. These acrylates are typically used as curing resins, coating material or in general for the development of new materials for a wide range of applications. Table 6 displays an overview of our results using diacrylates. Reaction of the diacrylate with 2.2 equivalents of diphenylphosphine and 8 equivalents of 2-MeTHF gave the expected diphosphine with yields in the 73% to 91% range (6 examples) after 8 hours of reaction at 90 °C. Crystals suitable for X-ray diffraction studies of the oxidized diphosphine 28 were obtained from CH_2_Cl_2_/*n*-hexane. The molecular structure is shown in Fig. 2.

**Table 6:** Catalyst-free hydrophosphination of diacrylate derivatives in the presence of 4 equiv. of 2-MeTHF (based on alkene function)^a^

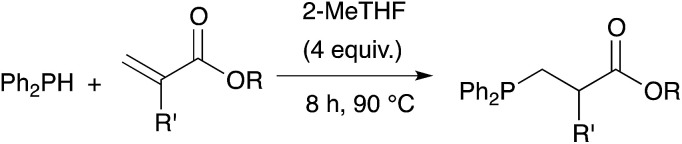
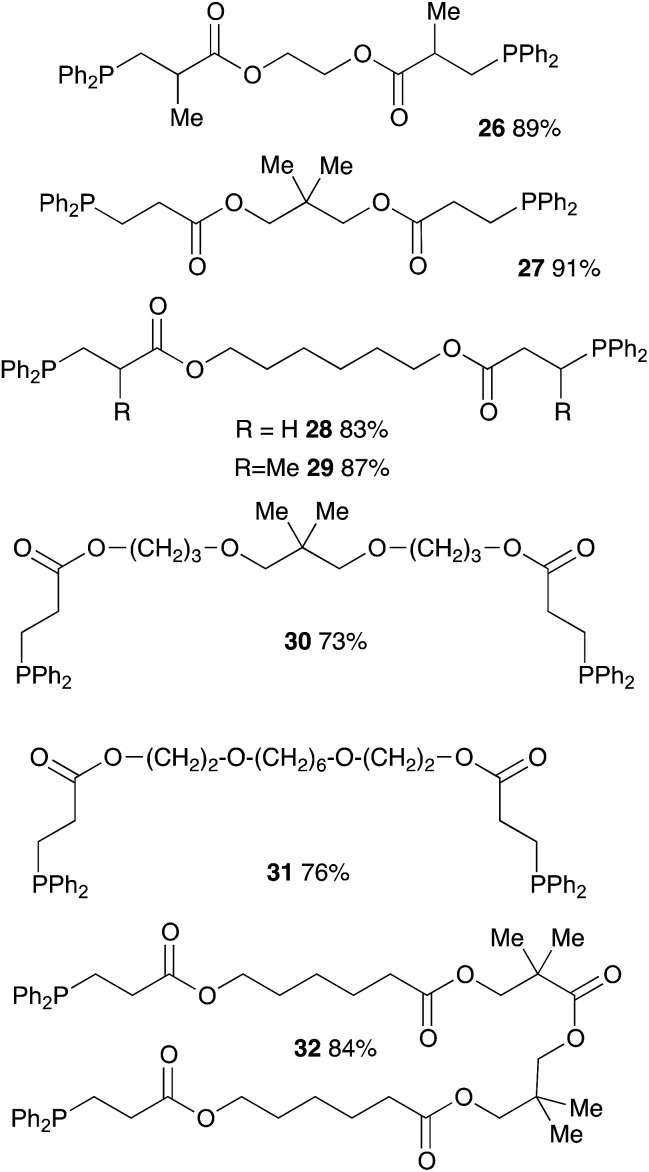

a Reaction condition: dialkene (1 equiv.), diphenylphosphine (2.2 equiv.), 2-MeTHF (8 equiv.) under argon.

Table 7 displays results with tris- and tetraacrylates which are also widely available. Again the compounds were obtained in good yields albeit with a longer reaction time (12 h). Isolated yields ranged from 57% (compound 35) to 91% (compound 34).

**Table 7:** Catalyst-free hydrophosphination of tris- and tetra-acrylate derivatives in the presence of 4 equiv. of 2-MeTHF^a^

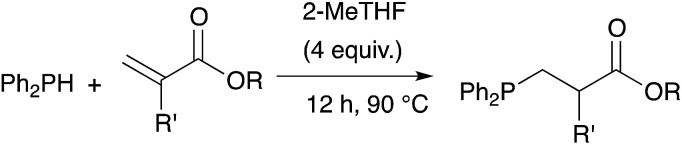
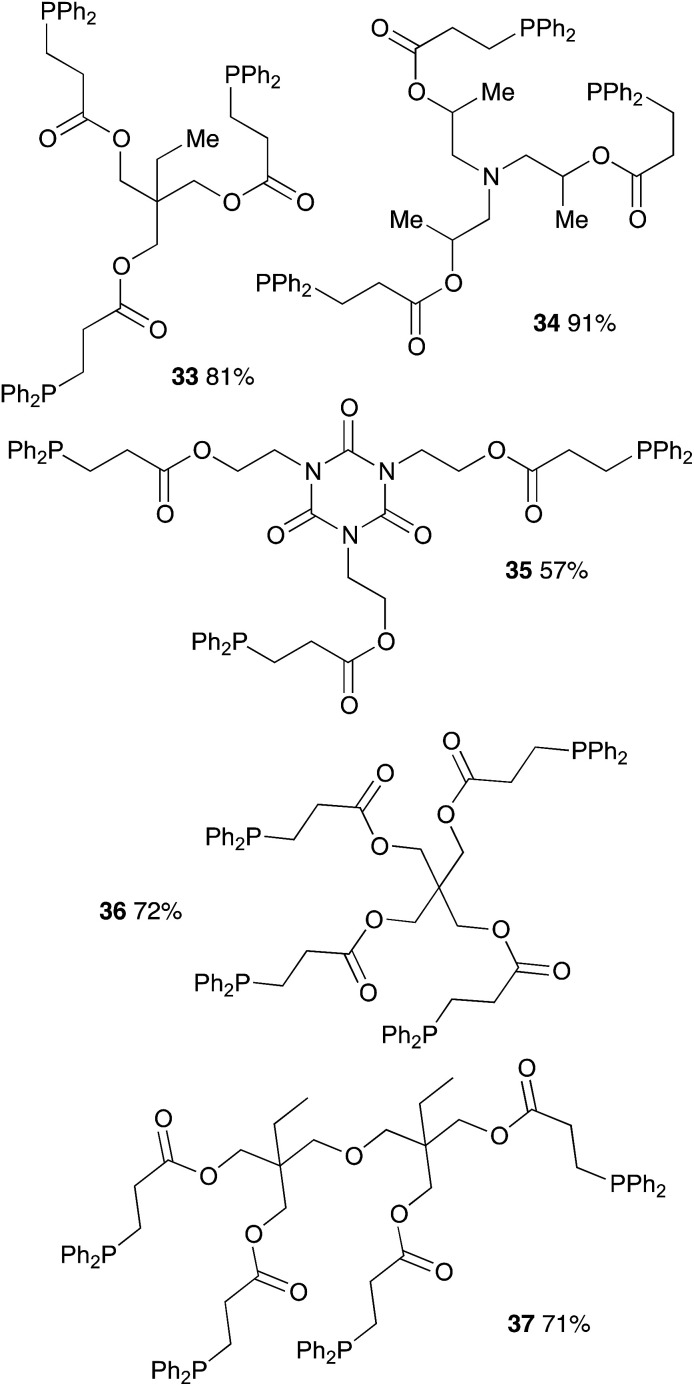

a Reaction condition: substrate (1 equiv.), diphenylphosphine (3.2 or 4.2 equiv.), 2-MeTHF (12 or 16 equiv.) under argon.

**Fig. 3:**
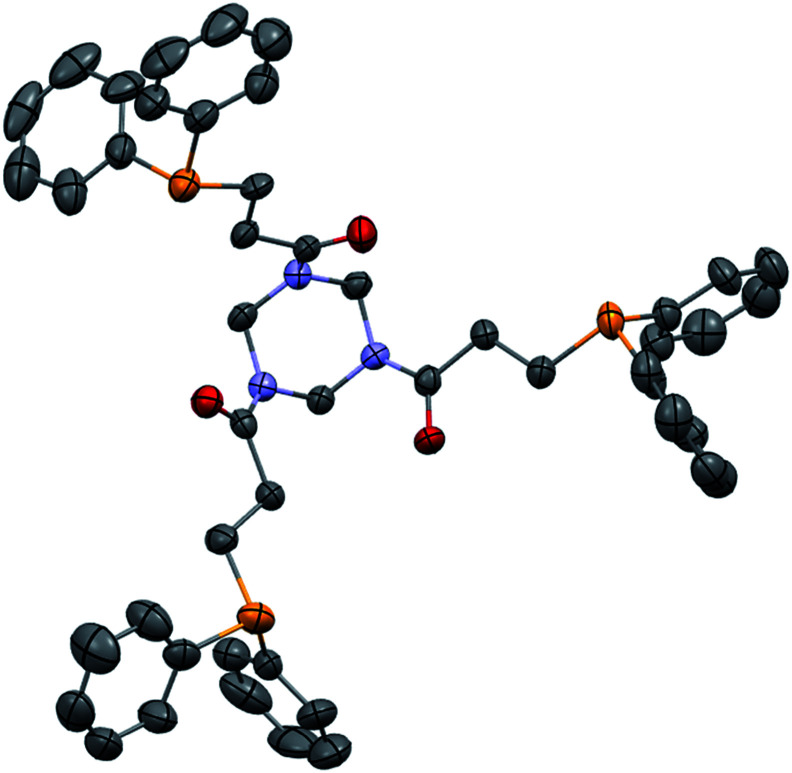
ORTEP representation of triphosphine 40, with thermal ellipsoids drawn at the 50% probability level.

Finally, polyacrylamides constitute a very important family of macromolecules that are usually hydrophilic biocompatible polymers and find various applications in biology (molecular biology), in medicine or in pharmaceutical science (drug delivery, contact lenses, *etc.*).^33,34^ In addition, they are used in wastewater treatment processes, paper processing, and mining and mineral processing.^35^ The interesting properties of these polymers have prompted industrial groups to constantly develop new acrylamide monomers. This therefore offers a range of interesting substrates for us that should give original and novel phosphines. Table 8 displays some examples of such phosphine-based acrylamide derivatives (reaction conditions: 90 °C for 12 h). Overall, yields with acrylamides are lower than classical acrylates. For example, the hydrophosphination of *N*,*N*-dimethylacrylamide gave the expected product 38 with 78% yield. Methacrylamides are also reactive toward hydrophosphination, such as compound 39, which was obtained with 63% yield. The molecular structure of triphos 40, which could be an interesting metal-complexing ligand, has been determined by X-ray diffraction studies (Fig. 3).

**Table 8:** Catalyst-free hydrophosphination of acrylamide derivatives in the presence of 4 equiv. of 2-MeTHF^a^

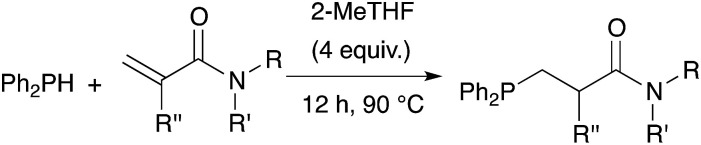
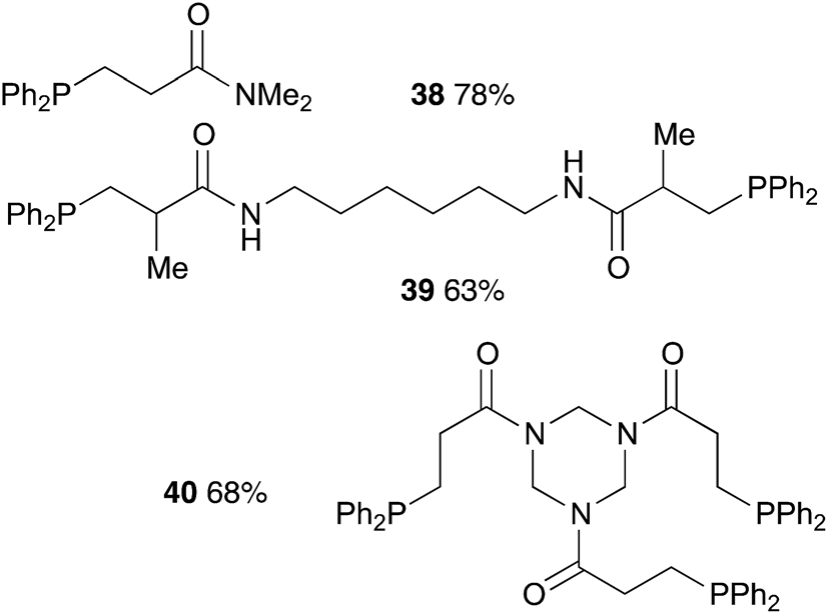

a Reaction condition: substrate (1 equiv.), diphenylphosphine (1.2, 2.2 or 3.2 equiv.), 2-MeTHF (4, 8, or 12 equiv.) under argon.

## Conclusions

We have shown that a large range of tertiary diphenylphosphine derivatives can be readily obtained in high yields by direct reaction of diphenylphosphine and alkene in presence of few equivalents of bio-mass derived 2-Me-THF. The presence of 2-MeTHF into the reaction media improves both conversion and time of the reaction and reduces the sensitivity of the reactants over oxidation. This procedure is applicable to styrene derivatives, α,β-unsaturated esters and amides and the corresponding products were all formed with anti-Markovnikov regioselectivity. Moreover, the easy availability of acrylic monomers allowed us to synthesize a wide range of polyphosphines that may of interest to the industry. It allowed us access to di-, tri- and tetra-phosphines with good to excellent isolated yields in all cases. This simple, straightforward and atom-economy method follows the principles of Green Chemistry.

## General experimental procedure

### General hydrophosphination procedure

All phosphines were prepared in 0.5 g to 10 g scale by addition of the corresponding alkene compounds on diphenylphosphine (PPh_2_H) in presence of 2-MeTHF (4 equiv.) under argon in a closed vessel. The mixture was degassed under vacuum three times then the stirred during 4 to 12 h at 90 °C under argon in a closed vessel. Hydrophosphination products were then purified by flash chromatography on silica gel (gradient from cyclohexane to cyclohexane/ethyl acetate 8 : 2) (for more details see ESI†).

## Conflicts of interest

The authors declare that they have filed a provisional patent application.

## Supplementary Material

RA-009-C9RA04896K-s001

RA-009-C9RA04896K-s002
