# Supplementary material for: Catalyst-free hydrophosphination of alkenes in presence of 2-methyltetrahydrofuran: a green and easy access to a wide range of tertiary phosphines
Source: RSC Adv. 2019 Aug 30;9(47):27250–6. doi: 10.1039/c9ra04896k (PMC9070578; doi:10.1039/c9ra04896k)

Electronic Supplementary Material (ESI) for RSC Advances.
This journal is © The Royal Society of Chemistry 2019

Catalyst-free hydrophosphination of alkenes in presence of 2-methyltetrahydrofuran : a green and easy access to a wide range of tertiary phosphines.

Damien Bissessar, Julien Egly, Thierry Achard, Pascal Steffanut and Stéphane Bellemin-Laponnaz*

Electronic Supporting Information

1 GENERAL REMARKS S2
2 GENERAL PROCEDURE FOR THE SYNTHESIS OF MONO-, DI AND TRI-ACRYLATES S3
3 GENERAL HYDROPHOSPHINATION PROCEDURE S3
4 ^{1}H,^{13}C and ^{31}P NMR SPECTRA OF ALL COMPOUNDS S14
4.1 Monoacrylate derivatives S14
4.2 Monomethacrylate derivatives S33
4.3 Tris- and tetra-acrylate derivatives S61

1 GENERAL REMARKS

All manipulations were carried out under an inert atmosphere of argon using standard Schlenk techniques unless stated otherwise. Reagents were purchased from commercial chemical suppliers (mainly Acros, Aldrich, Alfa Aesar, TCI Europe and Strem) and used without further purification. Solvents were dried and degassed according to standard procedures. $HPPh_2$ was purchased from commercial resource from STREM, ALDRICH or SOLVAY. ^{1}H, ^{13}C {^{1}H}, ^{19}F and ^{31}P nuclear magnetic resonance (NMR) spectra were recorded on a Bruker Avance 300 spectrometer and a Bruker Avance III HD - 500 MHz. ^{13}C assignments were confirmed when necessary with the use of DEPT 135 experiments. ^{1}H and ^{13}C-NMR spectra were referenced using the residual solvent peak ($CDCl_3$: δ H = 7.26 ppm; δ C = 77.16 ppm) at 295K. Chemical shifts δ are given in ppm whereas coupling constants *J* are stated in Hertz (Hz). The following abbreviations are used to classify the multiplicity of the observed signals: s = singlet, d = doublet, t = triplet, q = quartet, quint = quintuplet, dd = doublet from doublet, dt = doublet from triplet, m = complex multiplet or broad signal. Positive mode electrospray ionization mass spectra (ESI-MS) were recorded on microTOF, Bruker Daltonics. X-Ray diffraction studies were carried out by Corinne Bailly and Dr Lydia Karmazin at Institut de Chimie X-ray Facility of the University of Strasbourg. Crystal data were collected at 173 K using a MoKα graphite monochromated (λ = 0.71073 Å) radiation on a Nonius KappaCCD diffractometer. The structures were solved using direct methods with SHELXS97552 and refined against F2 using the SHELXL97 software. Non-hydrogen atoms were refined anisotropically. Hydrogen atoms were generated according to stereochemistry and refined using a riding model in SHELXL97. These data can be obtained free of charge from The Cambridge Crystallographic Data Centre via www.ccdc.cam.ac.uk/data_request/cif
All manipulations were conducted under an argon atmosphere unless otherwise stated.

2 GENERAL PROCEDURE FOR THE SYNTHESIS OF MONO-, DI AND TRI-ACRYLATES

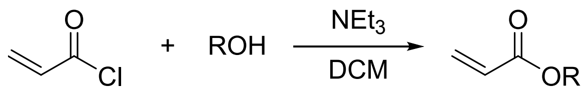

All acrylates were obtained using standard synthetic procedures in 1 g to 4 g scale by addition of acryloyl chloride to the desired alcohol in dichloromethane (or diethyl ether) during 4h in presence of trimethylamine at 25 °C. The acryloylchloride was added dropwise at 0 °C and then the resulting solution was stirred to room temperature for 30 minutes. A white precipitate appeared instantaneously during the slow addition (TEA^+Cl^- salt). At the end of the reaction, the mixture was filtered on celite to eliminate the salt and the crude product was purified through a silica column using dichloromethane as a eluent. The solution was collected, concentrated, and vacuum dried to afford the expected product. It's also possible to eliminate the salt by washing with saturated potassium carbonate solution.

3 GENERAL HYDROPHOSPHINATION PROCEDURE

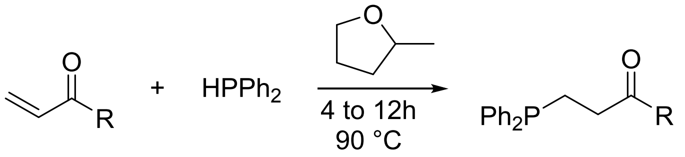

All phosphine (mono and di) and phosphine-ester (mono, di, tri and tetra) are prepared in 0.5 g to 20 g scale by addition of the corresponding alkene compounds on diphenylphosphine (PPh_2H) in MeTHF (4 equiv.) under argon. Diphenylphosphine was degassed under vacuum three times than the mixture was stirred during 4 to 12h at 90 °C under argon. Hydrophosphination products were then purified by flash silica gel chromatography (gradient from cyclohexane to cyclohexane/ethyl acetate 8:2).

Compound 5.

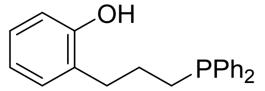

12h, 90 °C, 35% yield. ^{1}H NMR (300 MHz, $CDCl_3$, 20 °C): δ 1.78 (m, 2H, CH_2P), 2.12 (m, 2H, $\mathit{CH_2}CH_2P$), 2.27 (t, ^1J = 7.7 Hz, 2H, CH_2O), 6.88 (m, 2H), 7.28 (m, 2H), 7.32 (m, 6H), 7.48 (m, 4H) ppm; ^{31}P NMR (161 MHz, $CDCl_3$, 20 °C) δ: -17.1 ppm. MS (positive ESI) 321.13 m/z (%): $[M + H]^+$.

Compound 6.

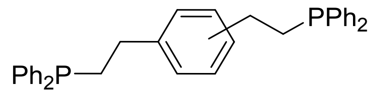

Compound is obtained as a mixture of several isomers. 8h, 110 °C, 85% yield. ^{1}H NMR (500 MHz, $CDCl_3$, 20 °C): δ 2.20-2.32 (m, 4H, CH_2P), 2.55-2.67 (m, 4H, CH_2CH_2P), 6.84-6.94 (m, 2H), 6.96-7.02 (m, 1H), 7.05-7.13 (m, 1H), 7.18-7.29 (m, 12H), 7.30-7.43 (m, 8H), ppm; ^{31}P NMR (161 MHz, $CDCl_3$, 20 °C) δ: -15.9 ppm.^{13}C NMR ($CDCl_3$, 202 MHz, 20 °C): δ 29.45-29.11 (m, CH_2-Ph), 30.84 (d, *J* = 17.8 Hz, CH_2-P), 31.18- 30.68 (m, CH_2-P), 124.57 (d, *J* = 16.6 Hz), 124.94, 127.39-126.60 (m), 127.56 (d, *J* = 6.6 Hz), 131.85 (d, *J* = 18.5 Hz), 137.72-137.51 (m), 139.37 (d, *J* = 13.2 Hz), 141.69 (d, *J* = 13.4 Hz), 141.85 (d, *J* = 13.3 Hz), 143.50 ppm; ^{31}P NMR (202 MHz, $CDCl_3$, 20 °C) δ: -15.91, -15.60, -15.56 ppm. MS (positive ESI) 519.34 m/z (%): $[M + H]^+$; FTIR: ν = 3059, 2958, 2896, 1953, 1586, 1479, 1431, 1093, 1068, 1024, 728, 692 cm^{-1}; m.p. 63.0-68.0 °C.

Compound 7.

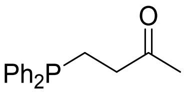

1h, rt, 88% yield. ^{1}H NMR (500 MHz, $CDCl_3$, 20 °C): δ): 2,10 (s, 3H, CH_3), 2,30 (m, 2H, CH_2P), 2,50 (m, 2H, CH_2O), 7,37-7,29 (m, 6H), 7,47-7,37 (m, 4H) ppm. ^{31}P NMR (202 MHz, $CDCl_3$, 20 °C):): δ -15,69 ppm

Analysis data correspond to the data reported in the literature. Ref : F.Alonso, Y. Moglie, G. Radivoy, M. Yusa *Green Chem.* **2012,** 14, 2699

Compound 8.

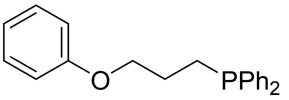

12h, 90 °C, 41% yield. ^{1}H NMR (300 MHz, $CDCl_3$, 20 °C): δ 1.95 (m, 2H, CH_2P), 2.25 (m, 2H, *CH_2*CH_2P), 4.03 (t, *J* = 6.1 Hz, 2H, CH_2O), 6.97 (m, 3H), 7.28 (m, 2H), 7.34 (m, 6H), 7.47 (m, 4H) ppm; ^{31}P NMR (161 MHz, $CDCl_3$, 20 °C) δ: -16.5 ppm. MS (positive ESI) 321.14 m/z (%): $[M + H]^+$.

Compound 7.

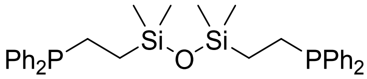

12h, 110 °C, 78% yield. ^{1}H NMR (500 MHz, $CDCl_3$, 20 °C): δ): 2,10 (s, 3H, CH_3), 2,30 (m, 2H, CH_2P), 2,50 (m, 2H, CH_2O), 7,37-7,29 (m, 6H), 7,47-7,37 (m, 4H) ppm. ^{31}P NMR (202 MHz, $CDCl_3$, 20 °C):): δ -15,69 ppm

Analysis data correspond to the data reported in the literature. Ref :

Compound 10.

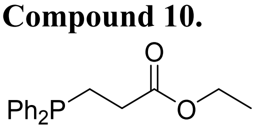

4h, 90 °C, 95% yield. ^{1}H NMR (400 MHz, $CDCl_3$, 20 °C): δ 1.26 (t, *J* = 7.1 Hz, 3H, CH_3), 2.28 (m, 4H, CH_2CH_2P), 4.01 (q, *J* = 7.1 Hz, 2H, CH_2O), 7.33-7.43 (m, 6H, CH_{arom}), 7.43-7.50 (m, 4H, CH_{arom}) ppm; ^{13}C NMR ($CDCl_3$, 101 MHz, 20 °C): δ 14.25, 23.04 (d, *J* = 12 Hz), 31.87 (d, *J* = 19 Hz), 60.64, 128.54 (d, *J* = 6.5Hz), 128.84, 132.86 (d, *J* = 19 Hz), 137.84 (d, *J* = 12.5 Hz), 173.23 (d, *J* = 15 Hz) ppm; ^{31}P NMR (162 MHz, $CDCl_3$, 20 °C) δ: -

15.64 ppm. MS (positive ESI) 287.12 m/z (%): [M +H]$^+$; FTIR: ν = 3072, 2980, 2872, 1731 (CO), 1480, 1431, 1371, 1348, 1216, 1165, 1026, 732, 691 cm^{-1}

Compound 11.

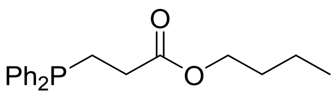

4h, 90 °C, 93% yield. ^{1}H NMR (400 MHz, $CDCl_3$, 20 °C): δ 0.83 (t, *J* = 7.4 Hz,3H), 1.22-1.31 (m, 2H), 1.43-1.54 (m, 2H), 2.23-2.35 (m, 4H), 3.96 (t, *J* = 6.7 Hz,2H), 7.20-7.28 (m, 6H), 7.35-7.38 (m, 4H) ppm; ^{13}C NMR ($CDCl_3$, 101 MHz, 20 °C): δ 13.74, 19.15, 20.02 (d, *J* = 12 Hz), 30.66, 30.78 (d, *J* = 20 Hz), 64.58, 128.52 (d, *J* = 6.7 Hz), 128.73, 132.84 (d, *J* = 18.6 Hz), 137.78 (d, , *J* = 12 Hz), 173.34 (d, *J* = 15.1 Hz) ppm; ^{31}P NMR (162 MHz, $CDCl_3$, 20 °C) δ: -15.71 ppm. MS (positive ESI) 315.17 m/z (%): [M +H]$^+$; FTIR: ν = 3057, 2993, 2871, 1728 (CO), 1459, 1431, 1155, 1026, 734, 696 cm^{-1}.

Compound 12.

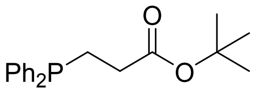

4h, 90 °C, 92% yield. ^{1}H NMR (500 MHz, $CDCl_3$, 20 °C): δ 1.44 (s, 9H), 2.31 (m, 4H), 7.33 (m, 6H), 7.44 (m, 4H) ppm; ^{31}P NMR (202 MHz, $CDCl_3$, 20 °C): δ -15.71 ppm. MS (positive ESI) 315.15 m/z (%): [M +H]$^+$; FTIR: ν = 3056, 2973, 2870, 1731 (CO), 1460, 1431, 1157, 1021, 737, 695 cm^{-1}.
Analysis data correspond to the data reported in the literature. Ref : F.Alonso, Y. Moglie, G. Radivoy, M. Yusa *Green Chem*. **2012,** 14, 2699

Compound 13.

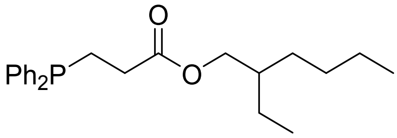

4h, 90 °C, 89% yield. ^{1}H NMR (400 MHz, $CDCl_3$, 20 °C): δ 0.77-0.81 (m, 6H), 1.10-1.31 (m, 8H), 1.42-1.49 (m, 1H), 2.21-2.37 (m, 4H), 3.88 (dd, J = 5.9, 1.5 Hz, 2H), 7.17-7.29 (m, 6H), 7.29-7.39 (m, 4H) ppm; ^{13}C NMR ($CDCl_3$, 101 MHz, 20 °C): δ 11.04, 14.09, 18.10, 23.01 (d, *J* = 12 Hz), 23.83, 28.96, 30.45, 30.92 (d, *J* = 19 Hz), 38.76, 67.18, 128.59 (d, *J* = 6.7 Hz), 128.81, 132.84 (d, *J* = 18.7 Hz), 137.91 (d, *J* = 12.7 Hz), 173.43 (d, *J* = 15.1 Hz) ppm; ^{31}P NMR (162 MHz, $CDCl_3$, 20 °C) δ: -15.64 ppm. MS (positive ESI) 371.23 m/z (%): [M +H]$^+$; FTIR: ν = 3054, 2959, 2864, 1734 (CO), 1462, 1435, 1379, 1343, 1219, 1158, 1022, 737, 689 cm^{-1}.

Compound 14.

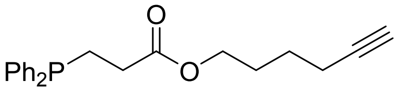

4h, 90 °C, 93% yield. ^{1}H NMR (400 MHz, $CDCl_3$, 20 °C): δ 1.43-1.54 (m, 2H), 1.59-1.70 (m, 2H), 1.86 (t, *J* = 2.7 Hz, 1H), 2.13 (td, *J* = 7.0, 2.7 Hz, 2H), 2.23-2.36 (m, 4H), 3.99 (t, , *J* = 6.5 Hz 2H), 7.22-7.29 (m, 6H), 7.31-7.38 (m, 4H) ppm; ^{13}C NMR ($CDCl_3$, 101 MHz, 20 °C): δ 18.10, 23.04 (d, *J* = 12 Hz), 24.94, 27.65, 30.86 (d, *J* = 19 Hz), 64.16, 68.81, 83.85, 128.54 (d, , *J* = 6.7 Hz), 128.83, 132.89 (d), 137.12 (d, *J* = 12.6 Hz), 173.26 (d, *J* = 15.0 Hz) ppm; ^{31}P NMR (162 MHz, $CDCl_3$, 20 °C) δ: -15.71 ppm. MS (positive ESI) 339.12 m/z (%): [M +H]$^+$; FTIR: ν = 3290, 3056, 2949, 1729 (CO), 1479, 1435, 1345, 1219, 1156, 1058, 735, 696 cm^{-1}.

Compound 15.

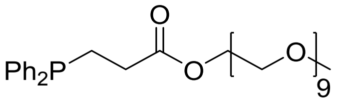

4h, 90 °C, 85% yield. ^{1}H NMR (500 MHz, $CDCl_3$, 20 °C): δ 2.30-2.37 (m, 2H), 2.38-2.46 (m, 2H), 3.32 (s, 3H), 3.49-3.55 (m, 2H), 3.58-3.68 (m, 28H), 4.15 (m, 2H), 7.29-7.35 (m, 6H), 7.37-7.44 (m, 4H) ppm; ^{13}C NMR ($CDCl_3$, 125 MHz, 20 °C): δ 22.90 (d, J = 12 Hz), 30.69 (d, J = 19 Hz), 59.04, 63.79, 69.08, 70.58, 70.63 and 70.67 (CH_2-O) , 71.95, 128.55 (d, J = 6.8 Hz), 128.84, 132.73 (d, J = 18.5 Hz), 137.63 (d, J = 12.2 Hz), 173.13 (d, J = 15.4 Hz) ppm; ^{31}P NMR (202 MHz, $CDCl_3$, 20 °C) δ: -15.63 ppm. MS (positive ESI) 669.28 m/z (%): $[M+H]^+$; FTIR: ν = 3056, 2874, 1729 (C_{CO}), 1433, 1343, 1270, 1219, 1097, 951, 849, 730, 698 cm^{-1}.

Compound 16.

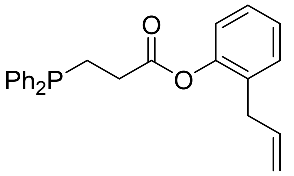

4h, 90 °C, 92% yield. ^{1}H NMR (400 MHz, $CDCl_3$, 20 °C): δ 2.37-2.41. (m, 2H), 2.50-2.65 (m, 2H), 3.18 (d, J = 6.5 Hz, 2H), 4.88-4.97 (m, 2H), 5.78 (ddt, J = 16.7, 10.2, 6.5 Hz, 1H), 6.91-6.93 (m, 1H), 7.05-7.17 (m, 3H), 7.22-7.32 (m, 6H), 7.34-7.42 (m, 4H) ppm; ^{13}C NMR ($CDCl_3$, 101 MHz, 20 °C): δ 23.12 (d, J = 12.5 Hz), 30.97(d, J = 19.8 Hz), 34.74, 116.23, 122.36, 126.26, 127.46, 128.71 (d, J = 6.7 Hz), 128.98, 130.45, 131.86, 132.89 (d, J = 18.7 Hz), 135.94, 137.24 (d, J = 12.6 Hz), 148.99, 171.66 (d, J = 15.4 Hz) ppm ; ^{31}P NMR (162 MHz, $CDCl_3$, 20 °C) δ: -15.77 ppm. MS (positive ESI) 375.12 m/z (%): $[M+H]^+$; FTIR: ν = 3056, 2911, 1751(CO), 1637,1579, 1484, 1435, 1352, 1207, 1165, 1117, 997, 912, 733, 694 cm^{-1}.

Compound 17.

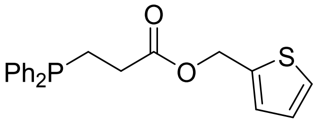

4h, 90 °C, 88% yield. ^{1}H NMR (500 MHz, $CDCl_3$, 20 °C): δ 2.29-2.34 (m, 2H), 2.34-2.42 (m, 2H), 5.18 (s, 2H), 6.93 (dd, J = 5.1, 3.5 Hz, 1H), 7.00-7.04 (m, 1H), 7.25 (dd, J = 5.1, 1.2 Hz, 1H), 7.26-7.32 (m, 6H), 7.34-7.41 (m, 4H) ppm; ^{13}C NMR ($CDCl_3$, 125 MHz, 20 °C): δ 22.86 (d, J = 12Hz), 30.72 (d, J = 19Hz), 60.73, 126.89 (d, J = 6.1 Hz), 128.55 (d, J = 6.7 Hz), 128.88, 132.77 (d, J = 18.5 Hz), 137.62 (d, J = 12.2 Hz), 137.82, 172.8 (d, J = 15.7 Hz) ppm; ^{31}P NMR (202 MHz, $CDCl_3$, 20 °C) δ: -15.59 ppm. MS (positive ESI) 355.08 m/z (%): $[M+H]^+$; FTIR: ν = 3064, 2937, 1729(CO), 1481, 1435, 1335, 1263, 1219, 1158, 944, 852, 735 cm^{-1}.

Compound 18.

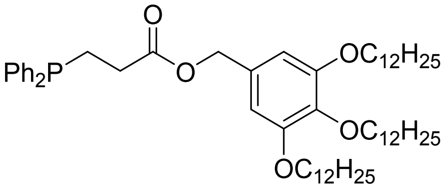

4h, 90 °C, 69% yield. ^{1}H NMR (400 MHz, $CDCl_3$, 20 °C): δ 0.88 (t, *J* = 6.8 Hz, 9H, CH_3), 1.26 (s, 48H, CH_2 alkyl), 1.45 (br, 6H, CH_2), 1.68-1.83 (m, 6H, CH_2), 2.34.2.50 (m, 4H), 3.93 (q, *J* = 6.4Hz, 6H), 4.98 (s, 2H), 6,51 (s, 2H, CH_{arom}), 7,30-7.36 (m, 6H, $CH_{Ph.}$), 7,37-7.44 (m, 4H, CH_{Ph}) ppm; ^{13}C NMR ($CDCl_3$, 101 MHz, 20 °C): δ 14.17, 22.77, 22.97 (d, *J* = 12Hz), 26.92, 29.42, 29.68, 29.74, 29.77, 30.53, 30.8 (d, *J* = 20Hz), 31.95, 66.90, 69.16, 73.44, 106.95, 128.55 (d, *J* = 7Hz), 128.84, 130.70, 132.73 (d, *J* = 18.5Hz), 137.66 (d, *J* = 12Hz), 138.19, 153.20, 173.01 (d, *J* = 15Hz) ppm; ^{31}P NMR (162 MHz, $CDCl_3$, 20 °C) δ: -15.74 ppm. MS (positive ESI) 901.70 m/z (%): $[M+H]^+$.

Compound 19.

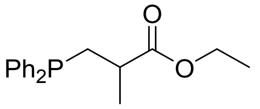

4h, 90 °C, 73% yield. ^{1}H NMR (400 MHz, $CDCl_3$, 20 °C): δ 1.13 (t, *J* = 7.2 Hz, 3H), 1.20 (d, *J* = 6.8 Hz, 3H), 1.99-2.09 (m, 1H), 2.36-2.49 (m, 2H), 3.92-4.03 (m, 2H), 7.19-7.28 (m, 6H), 7.29-7.41 (m, 4H) ppm; ^{13}C NMR ($CDCl_3$, 101 MHz, 20 °C): δ 14.22, 18.74 (d, *J* = 10 .2 Hz), 32.70 (d, *J* = 13.4 Hz), 37.27 (d, *J* = 17 Hz), 60.60, 128.55 (d, *J* = 6.8 Hz), 128.58 (d, *J* = 6.9 Hz), 128.75, 128.88, 132.79 (d, *J* = 16.3 Hz), 132.98 (d, *J* = 16.5 Hz), 137.93 (d, *J* = 12.4 Hz), 138.27 (dd *J* = 12.4 Hz), 138.63 (d, *J* = 13.4 Hz), 176.17 (d, *J* = 7 Hz) ppm; ^{31}P NMR (162 MHz, $CDCl_3$, 20 °C) δ: -19.62 ppm. MS (positive ESI) 301.13 m/z (%): $[M+H]^+$; FTIR: ν = 3069, 2978, 2932, 1731(CO), 1454, 1434, 1376, 1178, 1150, 1097, 1018, 732, 694 cm^{-1}.

Compound 20.

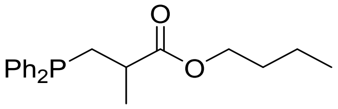

4h, 90 °C, 76% yield. ^{1}H NMR (400 MHz, $CDCl_3$, 20 °C): δ 0.82 (t, *J* = 7.4 Hz, 3H), 1.19 (d, *J* = 6.9 Hz, 3H), 1.21-1.32 (m, 2H), 1.43-1.52 (m, 2H), 1.98-2.07 (m, 1H), 2.34-2.48 (m, 2H), 3.85-3.98 (m, 2H), 7.15-7.26 (m, 6H), 7.27-7.39 (m, 4H). ppm; ^{13}C NMR ($CDCl_3$, 101 MHz, 20 °C): δ 13.78, 18.73 (d, *J* = 10.2 Hz), 32.72 (d, *J* = 13.6 Hz), 37.26 (d, *J* = 17.1 Hz), 64.45, 128.52 (d, *J* = 6.7 Hz), 128.56 (d, *J* = 6.9 Hz), 132.76 (d, *J* = 14.5 Hz), 132.95 (d, *J* = 14.6 Hz), 137.97 (d, *J* = 12.7 Hz), 138.64 (d, *J* = 12.5 Hz), 176.19 (d, *J* = 7.0 Hz) ppm; ^{31}P NMR (162 MHz, $CDCl_3$, 20 °C) δ: -19.65 ppm. MS (positive ESI) 329.45 m/z (%): $[M+H]^+$; FTIR: ν = 3064, 2955, 2874, 1731(CO), 1480, 1434, 1345, 1216, 1165, 1066, 737, 686 cm^{-1}.

Compound 21.

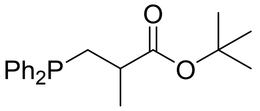

4h, 90 °C, 56% yield. ^{1}H NMR (400 MHz, $CDCl_3$, 20 °C): δ 1.16 (d, *J* = 6.9 Hz, 3H), 1.16 (d, *J* = 6.9 Hz, 3H), 1.35 (s, 9H), 1.98 (dd, *J* = 13.5, 7.8 Hz, 1H), 2.18-2.36 (m, 1H), 2.22-2.34 (m, 1H), 2.41 (ddd, *J* = 13.4, 6.6, 0.8 Hz, 1H), 7.18-7.28 (m, 6H), 7.29-7.41 (m, 4H) ppm; ^{13}C NMR ($CDCl_3$, 101 MHz, 20 °C): δ 18.69 (d, *J* = 10.1 Hz), 28.15, 32.79 (d, *J* = 13.4 Hz), 38.03 (d, *J* = 16.6 Hz), 80.41, 128.77, 128.71, 128.52 (d, *J* = 6.7 Hz), 128.57 (d, *J* = 6.7 Hz), 138.19 (d, *J* = 12.6 Hz), 138.82 (d, *J* = 12.7 Hz), 175.54 (d, *J* = 7.5 Hz), ppm; ^{31}P NMR (121 MHz, $CDCl_3$, 20 °C) δ: -19.49 ppm. MS (positive ESI) 329.20 m/z (%): $[M+H]^+$; FTIR: ν = 3054, 2973, 1726, 1462, 1431, 1363, 1343, 1140, 1023, 846, 737, 696 cm^{-1}.

Compound 22.

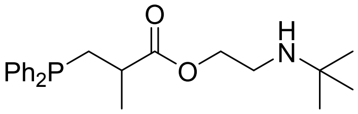

4h, 90 °C, 89% yield. ^{1}H NMR (400 MHz, $CDCl_3$, 20 °C): δ ^{1}H NMR (400 MHz, $CDCl_3$) δ 1.01 (s, 9H), 1.21 (d, *J* = 6.8 Hz, 3H), 2.05 (q, *J* = 10.0 Hz, 1H), 2.35-2.49 (m, 2H), 2.67-2.74 (m, 2H), 4.07 (t, *J* = 5.6 Hz, 2H), 7.21-7.29 (m, 6H), 7.29-7.41 (m, 4H) ppm; ^{13}C NMR ($CDCl_3$, 101 MHz, 20 °C): δ 18.84 (d, *J* = 10.1 Hz), 27.02, 29.13, 32.88 (d, *J* = 13.7 Hz), 37.17 (d, *J* = 17.0 Hz), 41.38, 50.31, 65.35, 128.58 (d, *J* = 1.9 Hz), 128.65 (d, *J* = 2.0 Hz), 128.84, 128.88, 132.88 (dd, *J* = 18.9, 0.7 Hz), 138.04 (d, *J* = 12.7 Hz), 138.51 (d, *J* = 12.7 Hz), 176.10 (d, *J* = 6.7 Hz) ppm; ^{31}P NMR (162 MHz, $CDCl_3$, 20 °C) δ: -20.03 ppm; MS (positive ESI) 372.11m/z (%): $[M+H]^+$; FTIR: ν = 3059, 2959, 1729 (CO), 1481, 1457, 1433, 1357, 1185, 1204, 1156, 1105, 1022, 737, 691 cm^{-1}.

Compound 23.

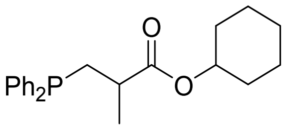

4h, 90 °C, 82% yield. ^{1}H NMR (400 MHz, $CDCl_3$, 20 °C): δ 1.32 (d, *J* = 7.0 Hz, 3H), 1.37-1.46 (m, 4H), 1.51-1.57 (m, 2H), 1.68-1.79 (m, 2H), 1.80-1.90 (m, 2H), 2.10-2.18 (m, 1H), 2.54-2.61 (m, 2H), 4.75-4.80 (m, 1H), 7.33-7.40 (m, 6 H), 7.43-7.51 (m, 4 H) ppm; ^{13}C NMR ($CDCl_3$, 101 MHz, 20 °C): δ 18.75 (d, *J* = 10.1 Hz). 23.70, 23.72, 25.51, 27.00, 31.57, 31.61, 32.78 (d, *J* = 13.7 Hz), 37.42 (d, *J* = 17.0 Hz), 72.62 (s), 128.53 (d, *J* = 6.8 Hz), 128.56 (d, *J* = 6.8 Hz), 128.71, 128.77, 132.78 (d, *J* = 4.0 Hz), 132.96 (d, *J* = 4.0 Hz), 138.17 (d, *J* = 13.0 Hz), 138.76 (d, *J* = 12.9 Hz), 175.60 (d, *J* = 7.2 Hz) ppm; ^{31}P NMR (162 MHz, $CDCl_3$, 20 °C) δ: -19.67 ppm; MS (positive ESI) 355.11 m/z (%): $[M+H]^+$; FTIR : ν = 3069, 2928, 2855, 1722(CO), 1450, 1430, 1194, 1153, 1012, 912, 740, 695 cm^{-1}.

Compound 24.

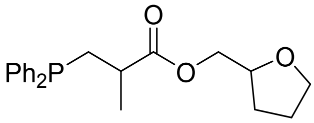

4h, 90 °C, 79% yield as a 1:1 mixture of two diastereomers. ^{1}H NMR (400 MHz, $CDCl_3$, 20 °C): δ 1.23 (d, *J* = 6.9 Hz, 3H), 1.44-1.58 (m, 1H), 1.73-1.94 (m, 3H), 2.00-2.11 (m, 1H), 2.42-2.53 (m, 2H), 3.65-3.81 (m, 2H), 3.84-4.07 (m, 3H), 7.19-7.28 (m, 6H), 7.29-7.39 (m, 4H) ppm ; ^{13}C NMR ($CDCl_3$, 101 MHz, 20 °C): δ 18.70 (d, *J* = 10.3 Hz), 25.73, 25.76, 26.97, 28.07, 32.62 (d, *J* = 13.8 Hz), 32.71 (d, *J* = 13.8 Hz), 37.12 (d, *J* = 17.2 Hz), 37.14 (d, *J* = 17.3 Hz), 66.58, 66.58, 68.48, 68.50, 128.52 (d, *J* = 6.8 Hz), 128.56 (d, *J* = 6.7 Hz), 128.70, 128.84, 132.67, 132.69, 132.86, 132.88, 133.03, 133.04, 137.92 (d, *J* = 12.8 Hz), 137.94 (d, *J* = 12.9 Hz), 138.54 (d, *J* = 12.5 Hz), 138.57 (d, *J* = 12.6 Hz), 176.11 (d, J = 7.4 Hz) ppm; ^{31}P NMR (162 MHz, $CDCl_3$, 20 °C) δ: -19.96 ppm; MS (positive ESI) 357.08 m/z (%): $[M+H]^+$; FTIR: ν = 3054, 2976, 2867, 1732(CO), 1481, 1450, 1430, 1185, 1151, 1080, 1024, 735, 695 cm^{-1}.

Compound 25.

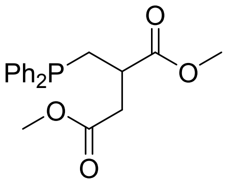

4h, 90 °C, 81% yield. ^{1}H NMR (300 MHz, $CDCl_3$, 20 °C): δ 2.24 (dd, *J* = 14.0, 8.2 Hz, 1H), 2.50-2.60 (m, 1H), 2.70-3.00 (m, 3H), 3.61 (s, 3H), 3.63 (s, 3H), 7.28-7.51 (m, 10H) ppm; ^{13}C NMR ($CDCl_3$, 75 MHz, 20 °C): δ 30.69 (d, *J* = 15.1 Hz), 36.45 (d, *J* = 10.7 Hz), 38.77 (d, *J* = 16.9 Hz), 51.76, 51.86, 128.59 (t, *J* = 7.1 Hz), 128.84, 129.01, 132.64 (d, *J* = 11.3 Hz), 132.90 (d, *J* = 11.6 Hz), 137.27 (d, *J* = 12.4 Hz), 137.95 (d, *J* = 12.2 Hz), 171.98, 174.68 (d, *J* = 6.9 Hz) ppm; ^{31}P NMR (121 MHz, $CDCl_3$, 20 °C) δ: -20.8 ppm; HRMS (positive ESI): m/z calcd. for $C_{19}H_{21}O_4P$ 344.1200, found 344.1215 ; FTIR: ν = 3059, 2996, 2952, 1729(CO), 1430, 1360, 1207, 1165, 1040, 740, 691 cm^{-1}.

Compound 26.

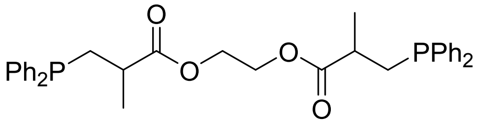

8h, 90 °C, 89% yield. ^{1}H NMR (500 MHz, $CDCl_3$, 20 °C): δ 1.18 (d, *J* = 6.2 Hz, 6H), 1.97-2.06 (m, 2H), 2.36-2.47 (m, 4H), 4.01-4.17 (m, 4H), 7.17-7.26 (m, 12H), 7.27-7.38 (m, 8H) ppm; ^{13}C NMR ($CDCl_3$, 125 MHz, 20 °C): δ 18.64 (dd, *J* = 10.3, 3.3 Hz), 32.59 (d, *J* = 13.9 Hz), 37.12 (dd, *J* = 17.2, 2.7 Hz), 62.25 (d, *J* = 2.2 Hz), 128.57 (dd, *J* = 6.6, 5.9 Hz), 128.76 (s), 128.90 (s), 132.85 (t, *J* = 19.8 Hz), 137.81 (dd, *J* = 12.8, 1.5 Hz), 138.51 (d, *J* = 12.6 Hz), 175.81 (d, *J* = 7.1 Hz) ppm; ^{31}P NMR (202 MHz, $CDCl_3$, 20 °C) δ: -19.88 ppm ; HRMS (positive ESI): m/z calcd. for $C_{34}H_{36}O_4P_2$ 570.2101, found 570.2113 ; FTIR: ν = 3052, 2974, 1731 (CO), 1452, 1433, 1382, 1147, 1091, 738, 694 cm^{-1}.

Compound 27.

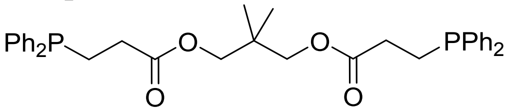

8h, 90 °C, 91% yield.^{1}H NMR (400 MHz, $CDCl_3$, 20 °C): δ 0.85 (s, 6H), 2.23-2.37 (m, 8H), 3.77 (s, 4H), 7.22-7.29 (m, 12H), 7.30-7.38 (m, 8H) ppm; ^{13}C NMR ($CDCl_3$, 101 MHz, 20 °C): δ 21.90. 23.04 (d, *J* = 12.1 Hz), 30.76 (d, *J* = 19.4 Hz), 34.78, 69.45, 128.69 (d, *J* = 6.7 Hz), 128.96, 132.84 (d, *J* = 18.7 Hz), 137.81 (d, *J* = 12.5 Hz), 173.15 (d, *J* = 15.0 Hz) ppm ; ^{31}P NMR (162 MHz, $CDCl_3$, 20 °C) δ: -15.83 ppm HRMS (positive ESI): m/z calcd. for $C_{35}H_{38}O_4P_2$ 584.2200, found 584.2207; FTIR: ν = 3052, 2967, 2860, 1731 (CO), 1476, 1433, 1373, 1214, 1148, 1047, 735, 693 cm^{-1} ; m.p. 76.0–82.0 °C.

Compound 28.

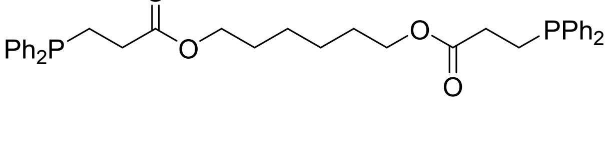

8h, 90 °C, 83% yield. ^{1}H NMR (500 MHz, $CDCl_3$, 20 °C): δ 1.16-1.28 (m, 4H), 1.40-1.56 (m, 4H), 2.19-2.34 (m, 8H), 3.92 (t, J = 6.7 Hz, 4H), 7.15-7.25 (m, 12H), 7.27-7.40 (m, 8H) ppm ; ^{13}C NMR ($CDCl_3$, 125 MHz, 20 °C): δ 21.96 (d, J = 12.1 Hz). 24.55, 27.45, 29.73 (d, J = 19.4 Hz), 63.54, 127.52 (d, J = 6.7 Hz), 127.78, 131.70 (d, J = 18.7 Hz), 136.79 (d, J = 12.7 Hz), 172.14 (d, J = 14.9 Hz) ppm; ^{31}P NMR (202 MHz, $CDCl_3$, 20 °C) δ: -15.71 ppm. HRMS (EI): m/z calcd. for $C_{36}H_{40}O_4P_2$ 598.2400, found 598.2408 ; FTIR : ν = 3052, 2923, 2860, 1731 (CO), 1476, 1433, 1358, 1224, 1166, 1067, 733, 694 cm^{-1}; m.p. 91.0–98.0 °C.

Compound 29.

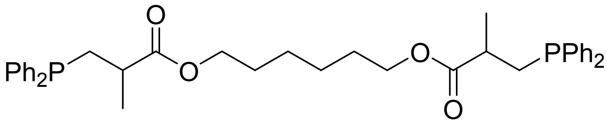

8h, 90 °C, 87% yield. ^{1}H NMR (400 MHz, $CDCl_3$, 20 °C): δ 1.34 (d, J = 6.8 Hz, 3H). 1.37-1.45 (m, 4H), 1.61-1.67 (m, 4H), 2.12-2.22 (m, 2H), 2.48-2.63 (m, 4H), 3.98-4.13 (m, 4H), 7.33-7.41 (m, 12H), 7.44-7.53 (m, 8H) ppm; ^{13}C NMR ($CDCl_3$, 101 MHz, 20 °C): δ 18.77 (d, J = 10.3 Hz) 25.65, 26.96, 28.54, 32.71 (d, J = 13.7 Hz), 37.25 (d, J = 17.2 Hz), 64.48, 128.52 (d, J = 6.7 Hz), 128.54 (d, J = 6.8 Hz), 128.72, 128.82, 132.73 (d, J = 11.8 Hz), 132.92 (d, J = 11.9 Hz), 137.97 (d, J = 12.8 Hz), 138.59 (d, J = 12.7 Hz), 176.12 (d, J = 6.9 Hz)ppm ; ^{31}P NMR (162 MHz, $CDCl_3$, 20 °C) δ: -19.69 ppm. HRMS (positive ESI): m/z calcd. for $C_{38}H_{44}O_4P_2$ 626.2706, found 626.2712; FTIR : ν = 3052, 2930, 2860, 1726 (CO), 1452, 1433, 1155, 1116, 1067, 739, 694 cm^{-1}.

Compound 30.

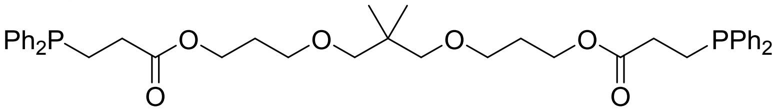

8h, 90 °C, 73% yield. ^{31}P NMR (202 MHz, $CDCl_3$, 20 °C) δ: -15.70, -15.75, -15.82 ppm; HRMS (positive ESI): m/z calcd. for $C_{47}H_{58}O_8P_2$ 812.3607, found 812.3612; FTIR: ν = 3065, 2973, 2864, 1727 (CO), 1477, 1434, 1368, 1220, 1152, 1066, 732, 693 cm^{-1}. Due to isomeric mixture of starting acrylate, both ^{1}H and ^{13}C signals are hard to attribute.

Compound 31.

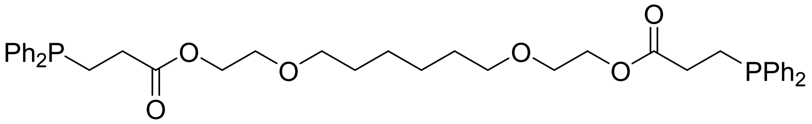

8h, 90 °C, 76% yield. ^{1}H NMR (400 MHz, $CDCl_3$, 20 °C): δ 1.24 (br, 4H) 1.40-1.55 (m, 4H), 2.20-2.40 (m, 8H), 3.33 (t, J = 6.7 Hz, 2H), 3.41-3.60 (m, 6H), 3.94 (t, J = 6.7 Hz, 2H), 4.05-4.14 (m, 2H), 7.17-7.28 (m, 12H), 7.28-7.38 (m, 8H). ppm; ^{13}C NMR ($CDCl_3$, 101 MHz, 20 °C): δ 22.86 (d, J = 12 Hz, CH$_2$-C(O)-O), 22.94 (d, J = 12 Hz, CH$_2$-C(O)-O), 25.54, 25.69, 25.86, 25.88 and 25.91(CH$_2$-CH$_2$), 28.44, 28.51, 29.42, 29.47, 29.48 and 29.54 (CH$_2$-CH$_2$), 30.57 (d, J = 12.0 Hz, CH$_2$-P), 30.76 (d, J = 11.9 Hz, CH$_2$-P), 63.37, 63.81, 64.53 and 64.66 (CH$_2$-O-C=O), 68.45 and 69.06 (CH$_2$.CH$_2$-O), 70.05, 70.56 and 71.37 (CH$_2$.CH$_2$-O), 128.51 (d, J = 6.7 Hz), 128.77 (s), 132.70 (d, J = 18.6 Hz), 137.69 (d, J = 4.8 Hz), 137.81 (d, J = 4.8 Hz), 173.08 (d, J = 15.2 Hz), 173.15 (d, J = 15.0 Hz).ppm; ^{31}P NMR (162 MHz, $CDCl_3$, 20 °C) δ: -15.72 ppm ; HRMS (positive ESI): m/z calcd. for $C_{40}H_{48}O_6P_2$ 686.2900, found 686.2956 ; FTIR: ν = 3054, 2935, 2864, 1729 (C_{CO}), 1435, 1219, 1122, 735, 696 cm^{-1}.

Compound 32.

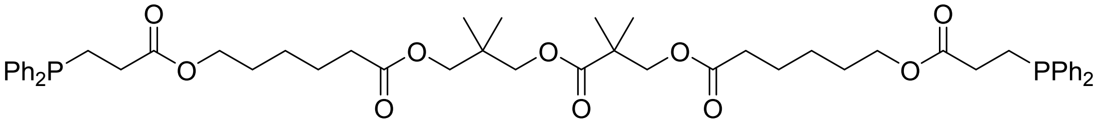

8h, 90 °C, 84% yield. ^{1}H NMR (400 MHz, $CDCl_3$, 20 °C): δ 0.87-1.00 (m, 6H). 1.17-1.24 (m, 6H), 1.30-1.43 (m, 4H), 1.55-1.69 (m, 6H), 2.26-2.44 (m, 12H), 3.82-3.92 (m, 4H), 3.99-4.07 (m, 4H), 4.08-4.13 (m, H), 7.29-7.37 (m, 12H), 7.37-7.46 (m, 8H) ppm. ^{13}C NMR ($CDCl_3$, 101 MHz, 20 °C): δ21.68, 21.76, 22.33 and 22.34 (*C*H_3), 22.90 (d, *J* = 12.1), 24.59, 24.61, 24.66 and 24.67 (*C*H_2-CH_2), 25.56, 25.60 and 25.63 (*C*H_2-CH_2), 28.38 and 25.45 (*C*H_2-CH_2) , 30.73 (d, *J* = 18.3 Hz, *C*H_2-P), 30.82 (d, *J* = 18.4 Hz, *C*H_2-P), 34.01, 34.12 and 34.21 (*C*H_2-C(O)-OCH_2), 34.99 (C), 42.88 (C), 64.20, 64.23, 64.53 and 64.56 (*C*H_2-CO-C(CH_3)$_2$), 69.04, 69.12, 69.41, 69.53, 69.99 and 70.29 (*C*H_2-CO-C(CH_3)$_2$), 128.63 (d, *J* = 6.7 Hz), 128.66 (d, *J* = 6.7 Hz), 128.90, 128.93, 137.79 (d, *J* = 12.6 Hz), 137.88 (d, *J* = 12.7 Hz), 172.90 (d, *J* = 15.4 Hz), 173.13, 173.27 (d, *J* = 15.4 Hz), 173.62, 175.29-175.35 (C=O). ^{31}P NMR (162 MHz, $CDCl_3$, 20 °C) δ: -15.75; -15.82 ppm; HRMS (positive ESI): m/z calcd. for $C_{52}H_{66}O_{10}P_2$ 912.4131, found 912.4167. FTIR: ν 3055, 2951, 1730 (CO), 1475, 1429, 1218, 1147, 1031, 730, 693 cm^{-1}.

Compound 33.

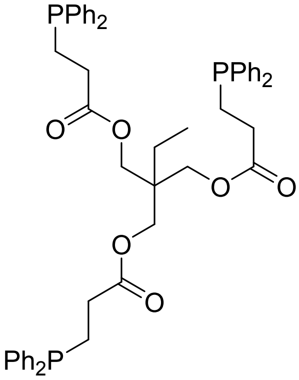

12h, 90 °C, 81% yield. ^{1}H NMR (500 MHz, $CDCl_3$, 20 °C): δ 0.84 (t, *J* = 7.6 Hz, 3H), 1.42 (q, *J* = 7.6 Hz, 2H), 2.23-2.46 (m, 12H), 3.97 (s, 6H), 7.29-7.36 (m, 18H), 7.37-7.44 (m, 12H) ppm; ^{13}C NMR ($CDCl_3$, 101 MHz, 20 °C): δ 7.52, 22.99 (d, *J* = 12.4 Hz), 30.70 (d, *J* = 19.5 Hz), 40.76, 64.07, 128.71 (d, *J* = 6.8 Hz), 128.98, 132.83 (d, *J* = 18.7 Hz), 137.74 (d, *J* = 12.6 Hz), 172.91 (d, *J* = 14.7 Hz) ppm; ^{31}P NMR (202 MHz, $CDCl_3$, 20 °C) δ: -15.94 ppm ; HRMS (positive ESI): m/z calcd. for $C_{51}H_{53}O_6P_3$ 824.3100, found 824.299 ; FTIR: ν = 3052, 2967, 2860, 1736 (CO), 1476, 1429, 1357, 1212, 1137, 1066, 733, 693 cm^{-1}; m.p. 100.0–104.0 °C.

Compound 34.

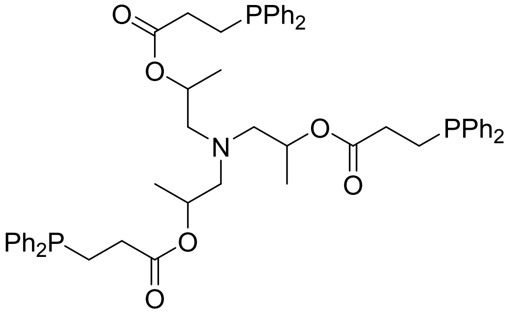

12h, 90 °C, 91% yield. ^{1}H NMR (500 MHz, $CDCl_3$, 20 °C): δ 1.19 (m, 9H), 2.29 (m, 12H), 2.70 (m, 6H), 5.02 (m, 3H), 7.26 (m, 18H, H_{arom}), 7.42 (m, 12H, H_{arom}) ppm; ^{13}C NMR ($CDCl_3$, 125 MHz, 20 °C): δ 18.21, 18.24 and 18.28 (*C*H_3), 23.0 (d, *J* = 12.0 Hz), 31.06 (d, *J* = 19.5 Hz), 60.07, 60.14 and 60.46 (*C*H_2-N), 69.15, 69.40 and 69.63 (*C*H), 128.61 (d, *J* = 6.7 Hz), 128.87, 132.79 (d, *J* = 18.7 Hz), 137.87 (d, *J* = 12.7 Hz),), 172.67 (d, *J* = 15.7 Hz); ^{31}P NMR (202 MHz, $CDCl_3$, 20 °C) δ: -15.73 ppm; MS (positive ESI) 912.32 m/z (%): $[M+H]^+$; FTIR: ν = 3058, 2976, 2825, 1720 (CO), 1434, 1363, 1220, 1167, 1057, 955, 732, 691 cm^{-1}.

Compound 35.

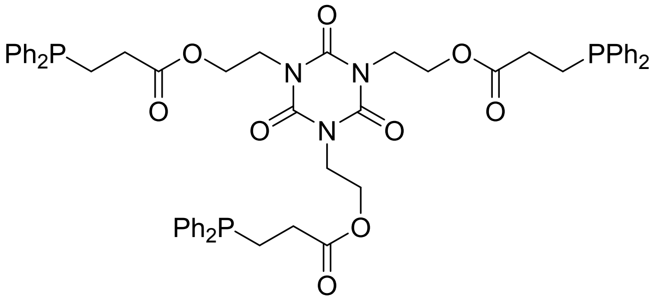

12h, 90 °C, 57% yield. ^{1}H NMR (400 MHz, $CDCl_3$, 20 °C): δ 2.21-2.43 (m, 12H). 4.03 (t, *J* = 5.1 Hz, 6H), 4.21 (t, *J* = 5.1 Hz, 6H), 7.29-7.34 (m, 18H), 7.37-7.43 (m, 12H) ppm; ^{13}C NMR ($CDCl_3$, 101 MHz, 20 °C): δ 22.85 (d, *J* = 12.0 Hz), 30.69 (d, *J* = 19.6 Hz), 42.06, 61.29, 128.67 (d, *J* = 6.7 Hz), 128.95, 132.84 (d, *J* = 18.7 Hz), 137.83 (d, *J* = 12.6 Hz), 149.01 (s), 173.07 (d, *J* = 15.6 Hz) ppm; ^{31}P NMR (162 MHz, $CDCl_3$, 20 °C) δ: -15.98 ppm; HRMS (positive ESI): m/z calcd. for $C_{54}H_{54}N_3O_9P_3$ 981.3100, found 981.3151; FTIR: ν = 3047, 2962, 2908, 1734 (CO), 1685, 1455, 1433, 1219, 1156, 908, 730, 696 cm^{-1}.

Compound 36.

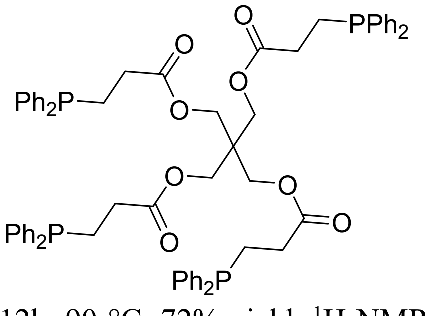

12h, 90 °C, 72% yield. ^{1}H NMR (500 MHz, $CDCl_3$, 20 °C): δ 2.26-2.32 (m, 8H), 2.32-2.40 (m, 8H), 4.03 (s, 8H), 7.28-7.34 (m, 24H), 7.36-7.41 (m, 16H); ^{13}C NMR ($CDCl_3$, 125 MHz, 20 °C): δ 22.95 (d, *J* = 12.7 Hz, CH_2P), 30.62 (d, *J* = 19.5 Hz, CH_2CH_2P), 42.00, 62.40 (CH_2O), 128.73 (d, *J* = 6.8 Hz, CHCHCP), 129.01, 132.84 (d, *J* = 18.7 Hz, CHCP), 137.68 (d, *J* = 12.6 Hz, CP), 172.65 (d, *J* = 15.8 Hz, C_{CO}) ppm; ^{31}P NMR (162 MHz, $CDCl_3$, 20 °C) δ: -16.03 ppm; HRMS (positive ESI): m/z calcd. for $C_{65}H_{64}O_8P_4$ 1096.3600, found 1096.3679; FTIR: ν = 3052, 2981, 2901, 1740 (CO), 1474, 1427, 1356, 1210, 1136, 1055, 734, 694 cm^{-1}; m.p. 170.0–175.0 °C.

Compound 37.

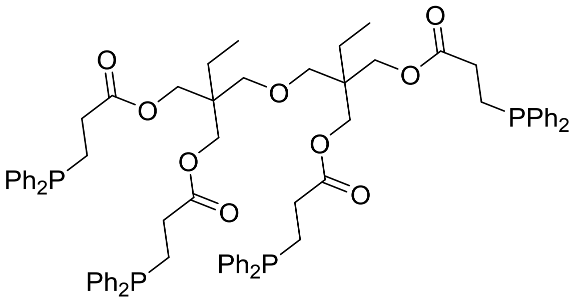

12h, 90 °C, 71% yield. ^{1}H NMR (400 MHz, $CDCl_3$, 20 °C): δ 0.78 (t, J = 7.5 Hz, 6H), 1.37 (q, J = 7.4 Hz, 4H), 2.18-2.46 (m, 16H), 3.18 (s, 4H), 3.94 (s, 8H), 7.29-7.36 (m, 24H), 7.39-7.44 (m, 16H), ppm; ^{13}C NMR ($CDCl_3$, 101 MHz, 20 °C): δ 7.60, 23.02 (d, J = 12.1 Hz), 30.72 (d, J = 19.5 Hz), 41.68, 64.62, 71.01, 128.70 (d, J = 6.8 Hz), 128.97, 132.84 (d, J = 18.7 Hz), 137.82 (d, J = 12.6 Hz), 172.95 (d, J = 14.9 Hz) ppm; ^{31}P NMR (162 MHz, $CDCl_3$, 20 °C) δ: -15.95 ppm; HRMS (positive ESI): m/z calcd. for $C_{72}H_{78}O_9P_4$ 1210.4600, found 1210.4608; FTIR: ν = 3059, 2967, 2860, 1732 (CO), 1474, 1432, 1348, 1214, 1154, 1116, 1067, 735, 693 cm^{-1}.

Compound 38.

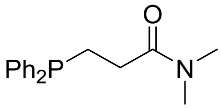

12h, 90 °C, 78% yield. ^{1}H NMR (500 MHz, $CDCl_3$, 20 °C): δ 2.36-2.43 (m, 4H), 2.86 (s, 3H), 2.91 (s, 3H), 7.31-7.35 (m, 6H), 7.43-7.47 (m, 4H), ppm; ^{13}C NMR ($CDCl_3$, 125 MHz, 20 °C): δ 23.16 (d, J = 9.8 Hz), 29.78 (d, J = 20.1 Hz), 35.65, 37.15, 128.61 (d, J = 6.8 Hz), 128.87 (s), 132.83 (d, J = 18.4 Hz), 138.08 (d, J = 11.7 Hz), 172.31 (d, J = 14.7 Hz) ppm; ^{31}P NMR (202 MHz, $CDCl_3$, 20 °C) δ: -15.20 ppm; MS (positive ESI) 292.15 m/z (%): $[M+Li]^+$; FTIR: ν = 2984, 2904, 1637 (CN), 1476, 1393, 1264, 1135, 1040, 735, 699 cm^{-1}.

Compound 39.

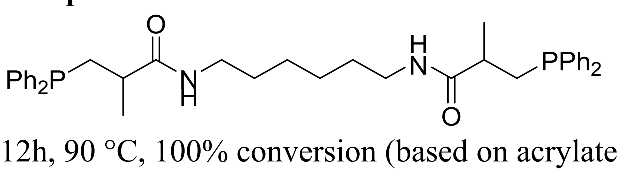

12h, 90 °C, 100% conversion (based on acrylate compound) . ^{1}H NMR (500 MHz, $CDCl_3$, 20 °C): δ 1.25 (d, J = 6.7 Hz), 1.31 (m, 4H), 1.45 (m, 4H), 2.16 (m, 4H), 2.49 (m, 2H), 3.16 (m, 4H), 5.48 (br, 2H), 7.33 (m, 12H, H_{arom}), 7.43 (m, 8H, H_{arom}) ppm; ^{13}C NMR ($CDCl_3$, 125 MHz, 20 °C): δ 19.59 (d, J = 11 Hz), 25.99, 29.42, 33.02 (d, J = 13 Hz), 38.94 (m), 128.52 (t), 128.74 (d), 132.78, 138.37, 175.58 (dd, J = 5.1 Hz) ppm; ^{31}P NMR (202 MHz, $CDCl_3$, 20 °C) δ: -19.29 ppm; MS (positive ESI) 625.31 m/z (%): $[M+H]^+$.

Compound 40.

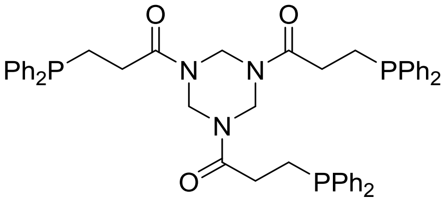

12h, 90 °C, 68% yield. ^{1}H NMR (500 MHz, $CDCl_3$, 20 °C): δ 2.32 (m, 6H, CH_2CH_2P), 2.59 (m, 6H, CH_2CH_2P), 5.10 (br, 6H, CH_2N), 7.33 (m, 18H, H_{arom}), 7.42 (m, 12H, H_{arom}) ppm; ^{13}C NMR ($CDCl_3$, 125 MHz, 20 °C): δ 22.80 (d, *J* = 11.1Hz, CH_2P), 29.31 (d, *J* = 20.5 Hz, CH_2CH_2P), 56.09 (CH_2N), 128.53 (d, *J* = 6.6 Hz, CHCHCP), 128.76, 132.77 (d, *J* = 18.7 Hz, CHCP), 137.98 (d, *J* = 12.9 Hz, CP), 153.68, 171.45 (d, *J* = 11.9 Hz, CO) ppm; ^{31}P NMR (202 MHz, $CDCl_3$, 20 °C) δ: -16.07 ppm; HRMS (positive ESI): m/z calcd. for $C_{48}H_{48}N_3O_6P_3$ 807.2900, found 807.2917; FTIR: ν = 3059, 2928, 1673 (CN), 1654, 1428, 1241, 1166, 1000, 942, 737, 694 cm^{-1}.

Compound 41 anti (crude).

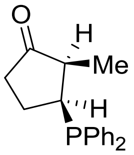

12h, 90 °C. ^{1}H NMR (500 MHz, $CDCl_3$, 20 °C): δ 1.11 (dd, 3H, CH_3, *J* = 7.7 & 2.2 Hz), 1.83 (m, 1H, CH_2), 1.92 (m, 1H, CH_2), 2.16 (m, 1H, CH_2), 2.50 (m, 1H, CH), 3.04 (m, 1H, P-CH), 7.32-7.36 (m, 6H), 7.48-7.56 (m, 4H) ppm; ^{13}C NMR ($CDCl_3$, 125 MHz, 20 °C): δ 14.62, 24.68 (d, *J* = 16.7Hz,), 37.52 (d, *J* = 5.8 Hz), 39.18 (d, *J* = 10.8 Hz), 46.24 (d, *J* = 12.6 Hz), 128.69 (d, *J* = 7.7 Hz), 129.36, 133.74 (d, *J* = 15 Hz), 133.84 (d, *J* = 15 Hz), 136.98 (d, *J* = 11.6 Hz), 137.15 (d, *J* = 12.9 Hz) ppm; ^{31}P NMR (202 MHz, $CDCl_3$, 20 °C) δ: -17.54 ppm

Compound 41 syn (crude).

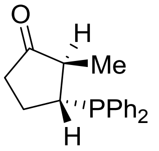

12h, 90 °C. ^{1}H NMR (500 MHz, $CDCl_3$, 20 °C): δ 0.77 (d, 3H, CH_3, *J* = 7.1 Hz), 1.65 (m, 1H, CH_2), 2.05 (m, 2H, CH_2), 2.16 (m, 1H, CH_2), 2.35 (m, 1H, CH), 2.52 (m, 1H, P-CH), 7.32-7.36 (m, 6H), 7.48-7.56 (m, 4H) ppm; ^{13}C NMR ($CDCl_3$, 125 MHz, 20 °C): δ 14.62, 24.84 (d, *J* = 13.7Hz,), 37.14 (d, *J* = 3.1 Hz), 40.79 (d, *J* = 12.4 Hz), 48.35 (d, *J* = 18.5 Hz), 128.69 (d, *J* = 7.7 Hz), 129.36, 133.74 (d, *J* = 15 Hz), 133.84 (d, *J* = 15 Hz), 136.31 (d, *J* = 13.8 Hz), 136.72 (d, *J* = 13.8 Hz), ppm; ^{31}P NMR (202 MHz, $CDCl_3$, 20 °C) δ: -4.68 ppm

4 ^{1}H,^{13}C and ^{31}P SPECTRA OF ALL COMPOUNDS

4.1 Monoacrylate derivatives

Compound 6

^{1}H NMR (500 MHz) $CDCl_3$

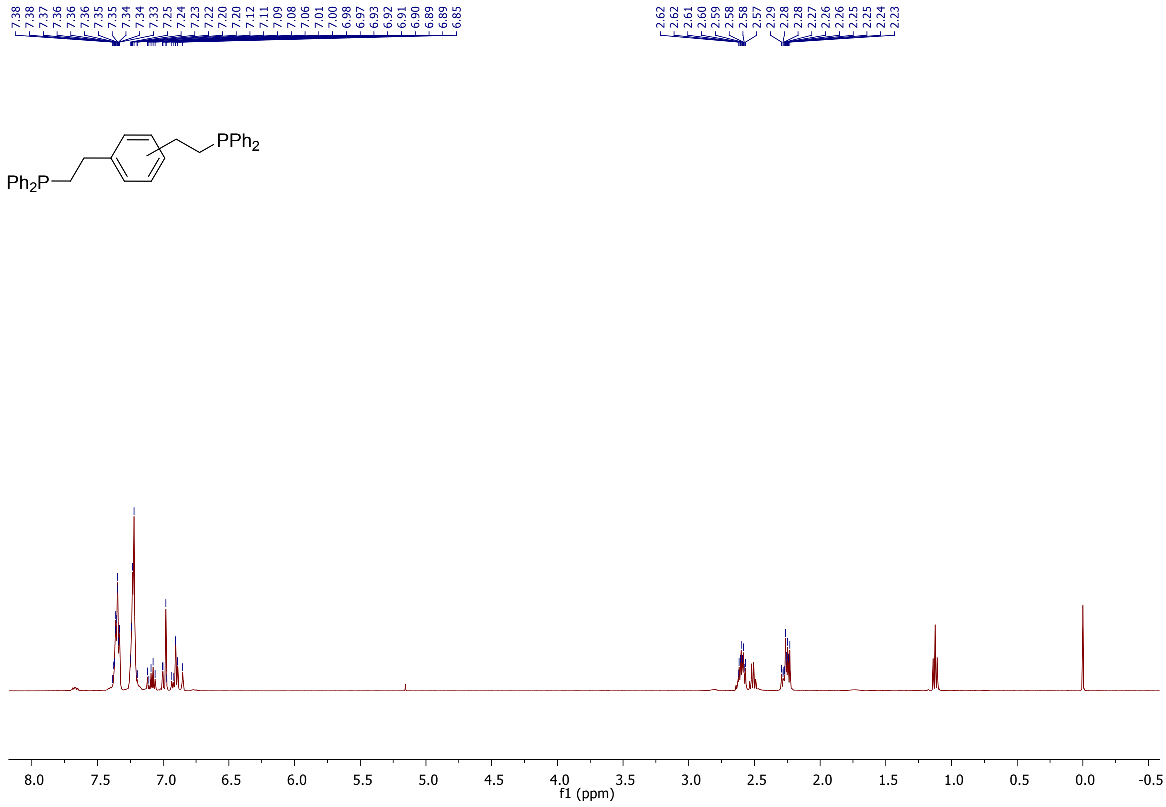

^{13}C NMR (125 MHz) $CDCl_3$

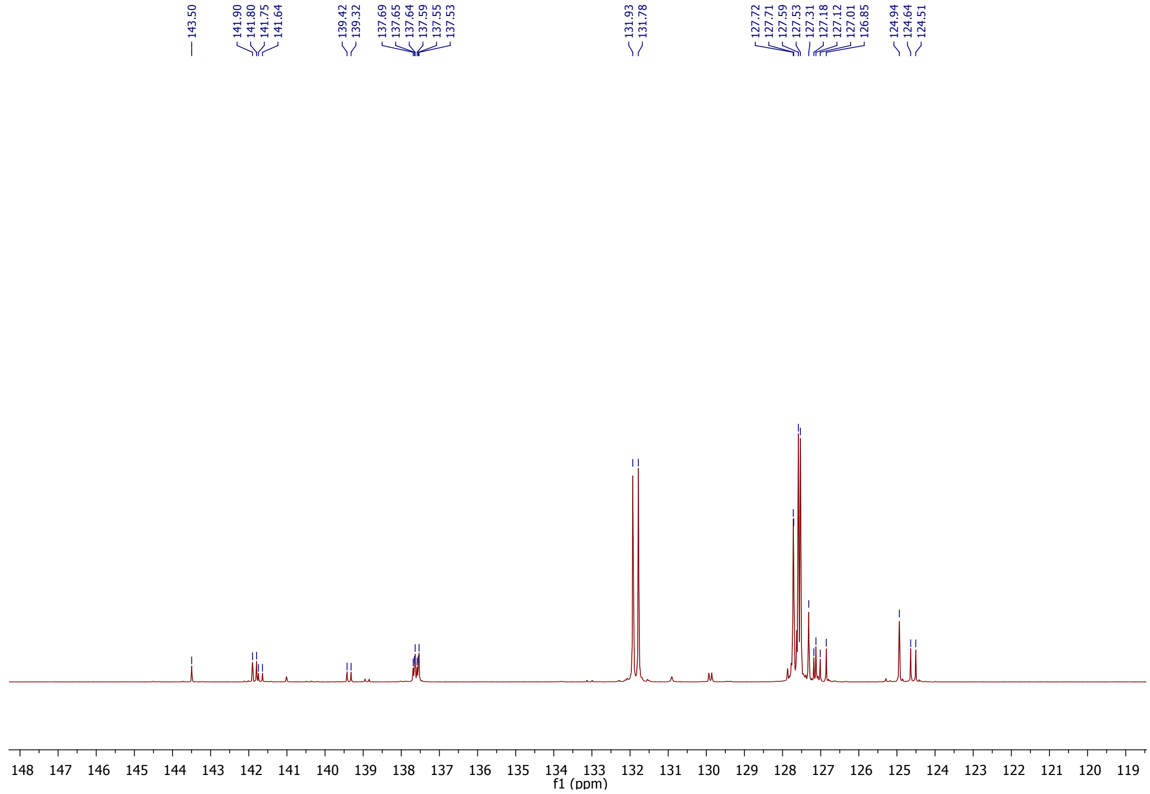

^{31}P NMR (202 MHz) $CDCl_3$

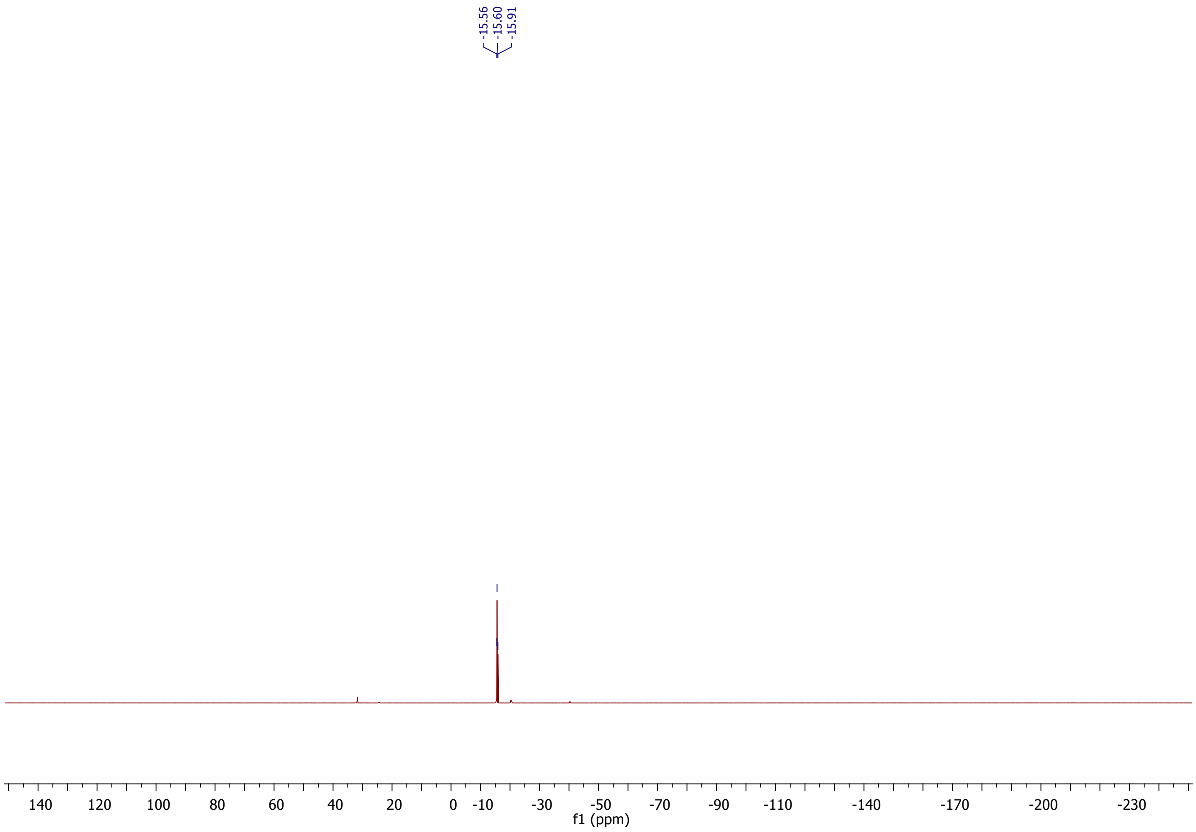

-15.56
-15.60
-15.91
140 120 100 80 60 40 20 0 -10 -30 -50 -70 -90 -110 -140 -170 -200 -230
f1 (ppm)

Compound 10

^{1}H NMR (400 MHz) $CDCl_3$

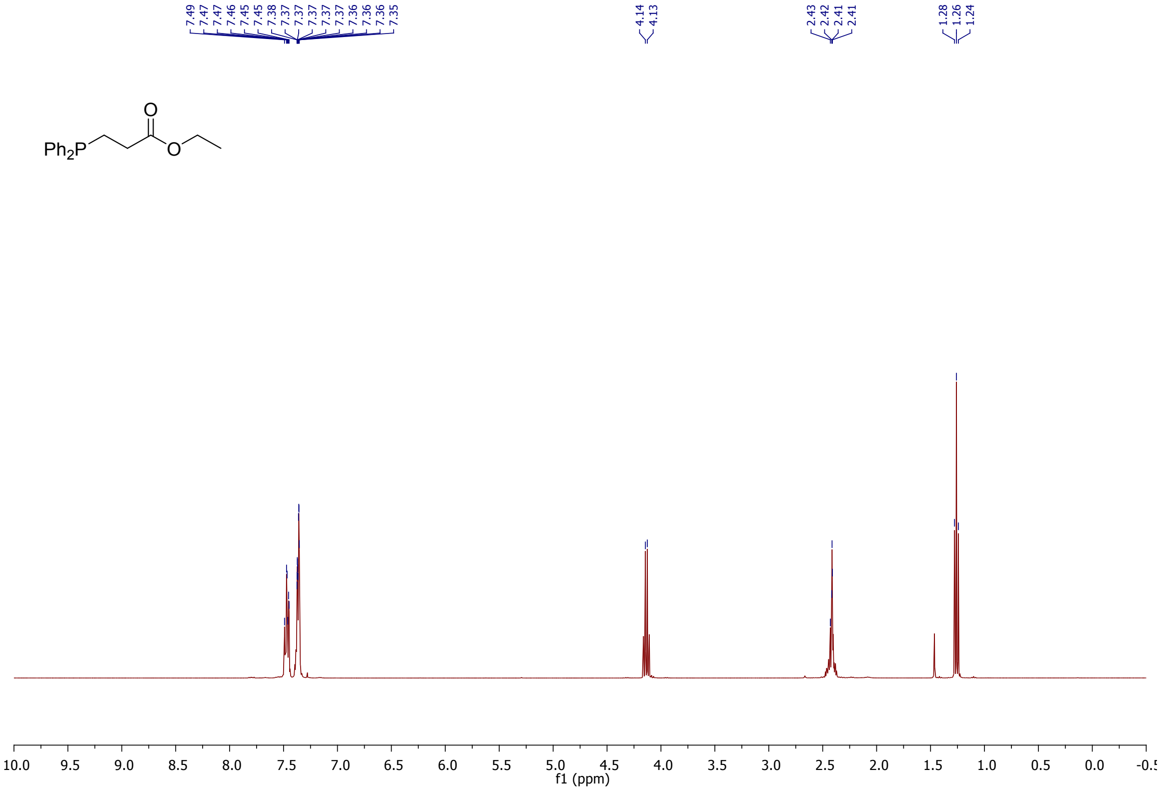

^{13}C NMR (101 MHz) $CDCl_3$

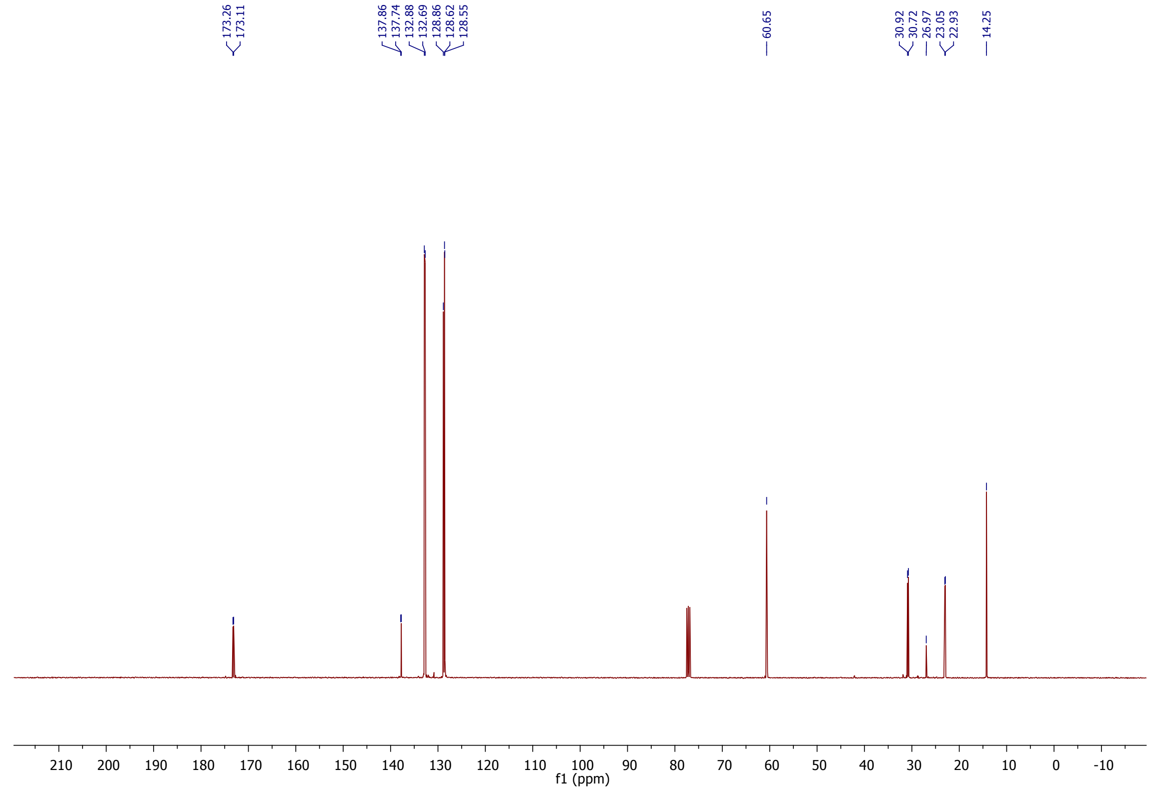

^{31}P NMR (162 MHz) $CDCl_3$

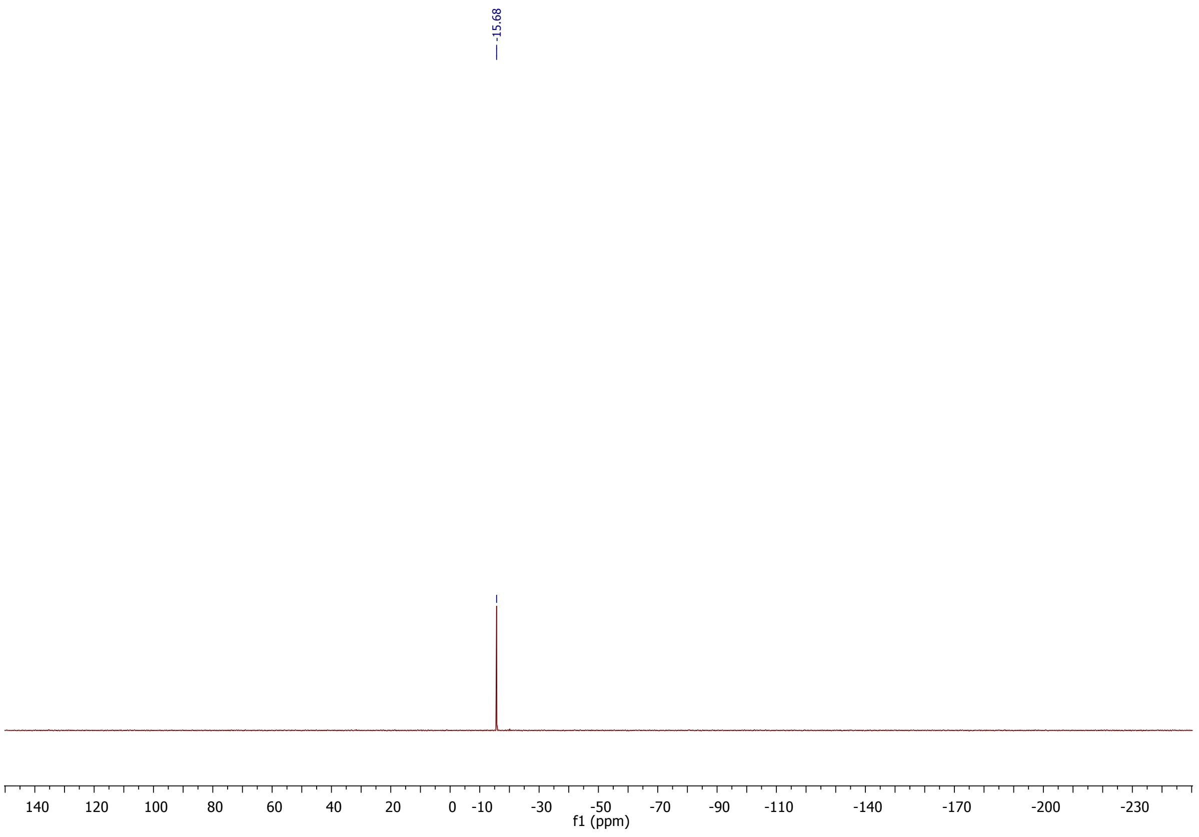

Compound 11

^{1}H NMR (400 MHz) $CDCl_3$

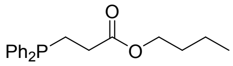

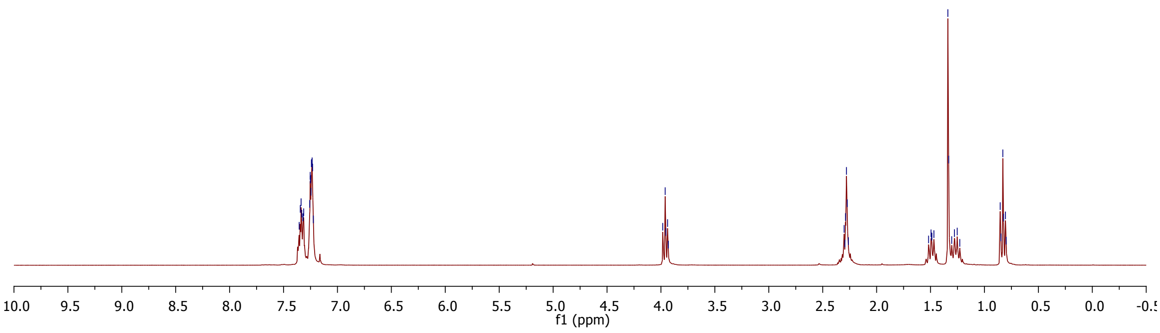

^{13}C NMR (101 MHz) $CDCl_3$

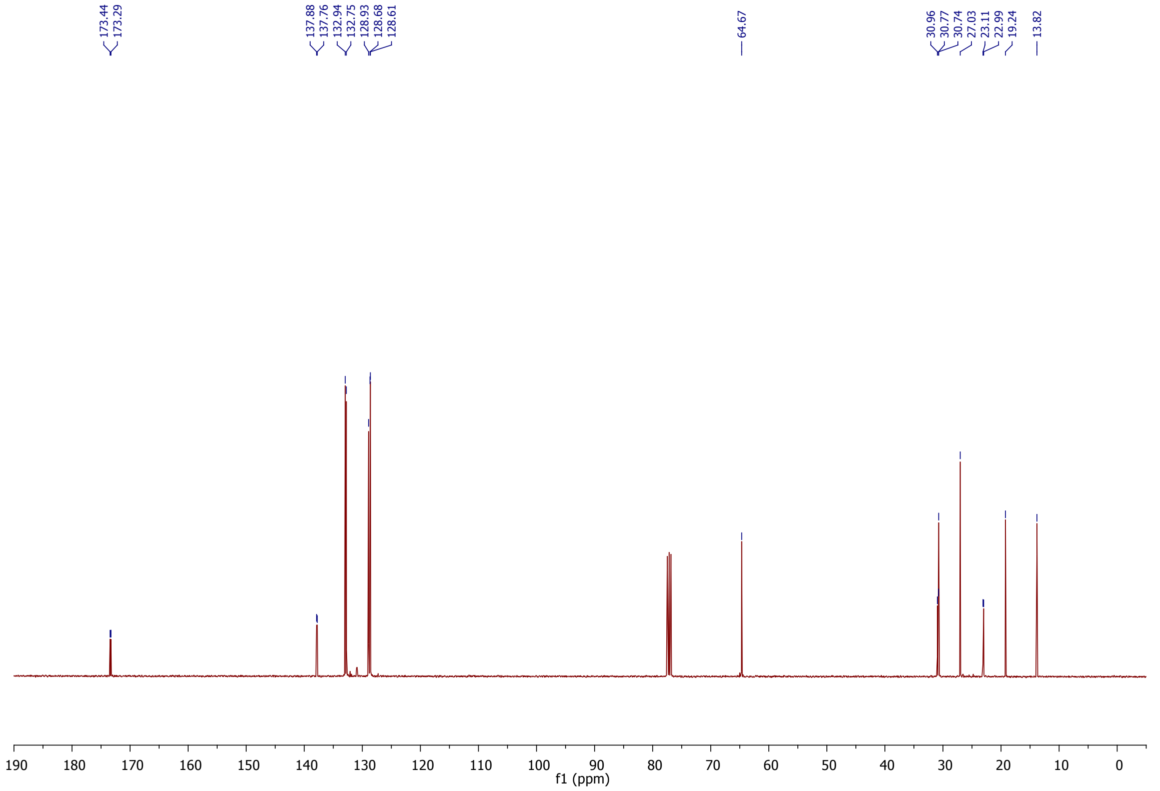

^{31}P NMR (162 MHz) $CDCl_3$

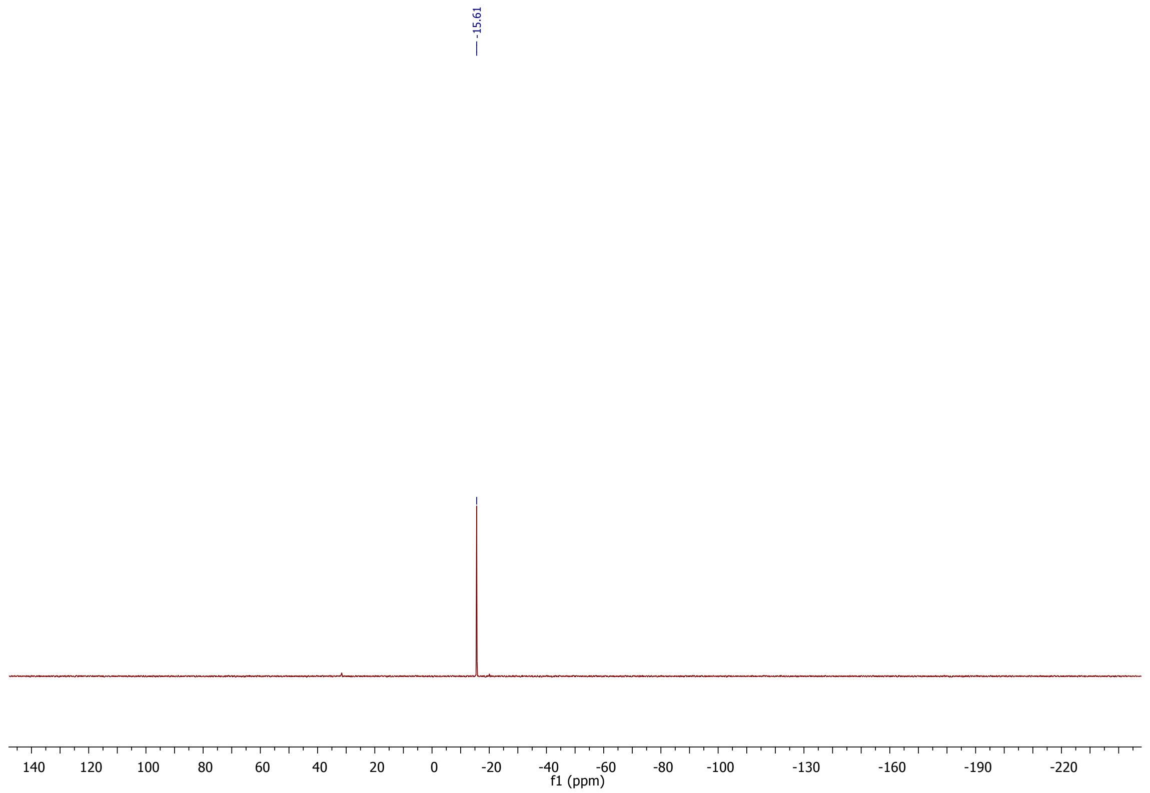

Compound 13

^{1}H NMR (500 MHz) $CDCl_3$

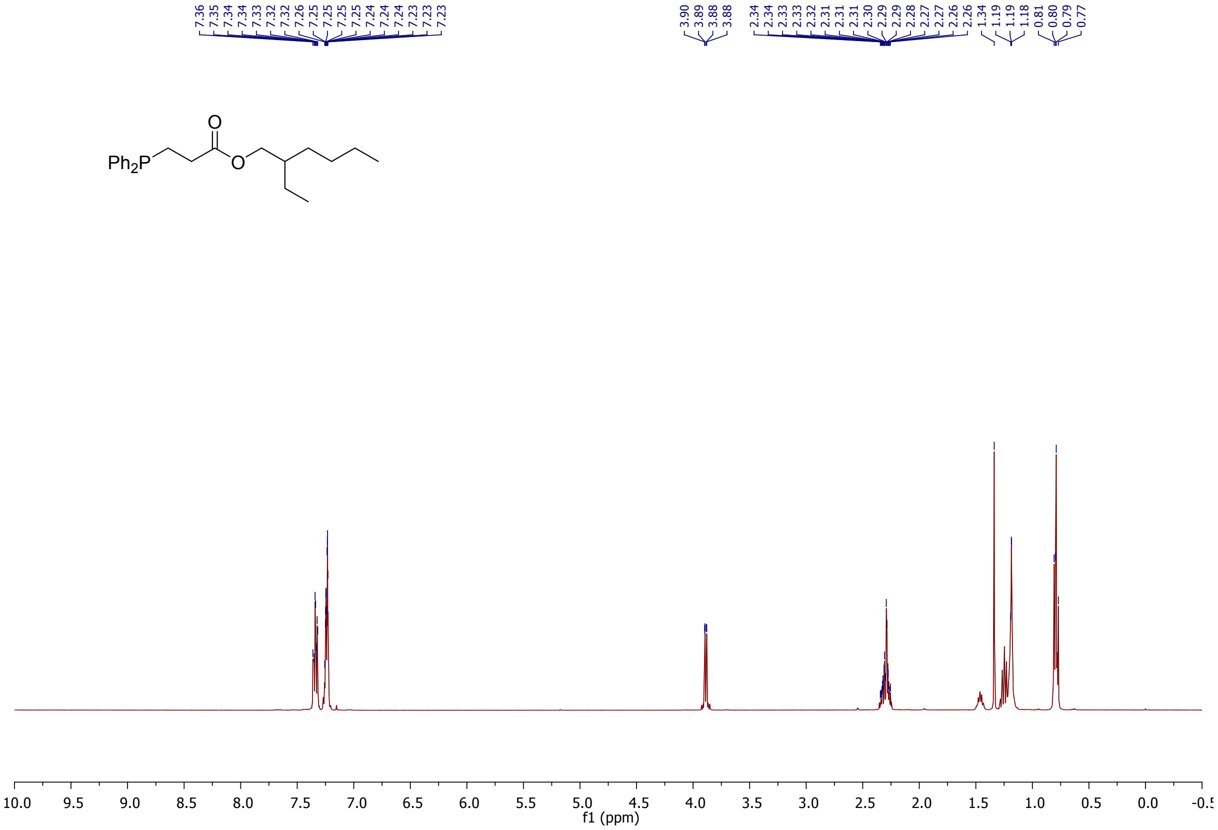

^{13}C NMR (125 MHz) $CDCl_3$

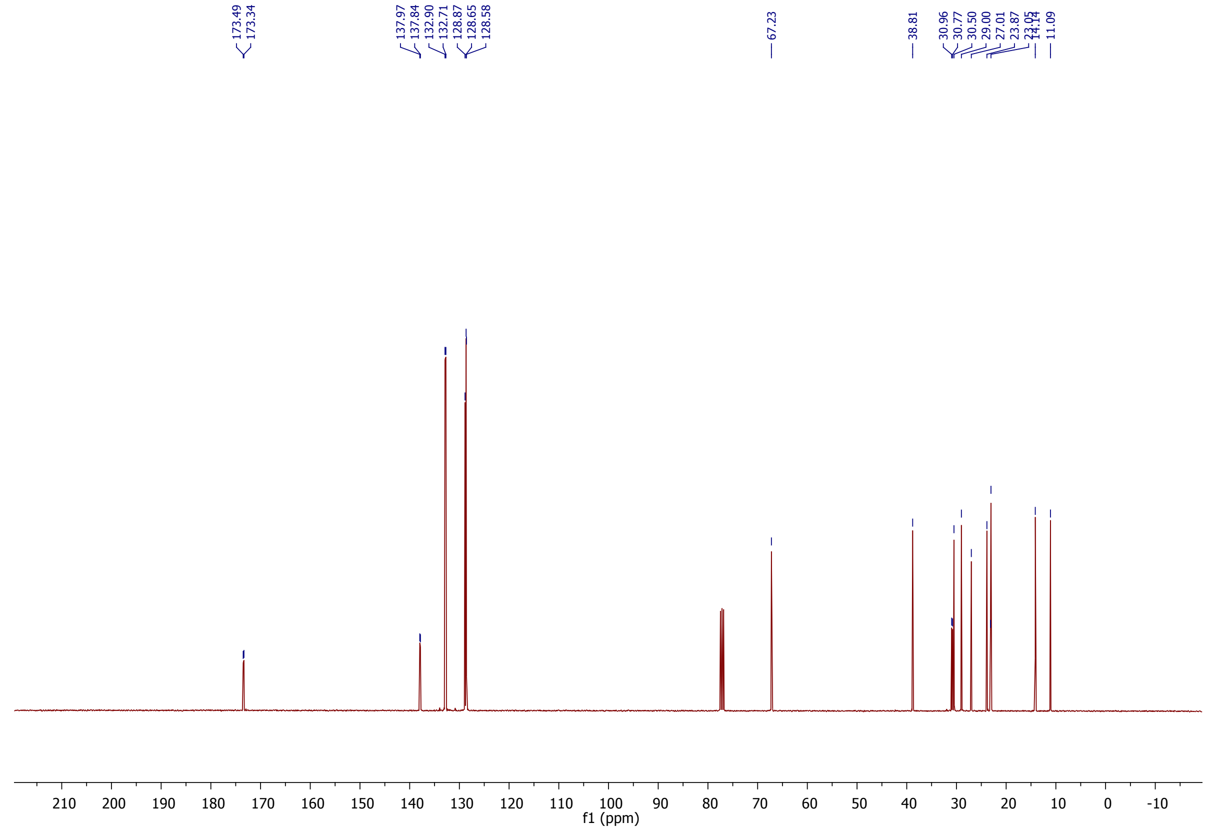

^{31}P NMR (202 MHz) $CDCl_3$

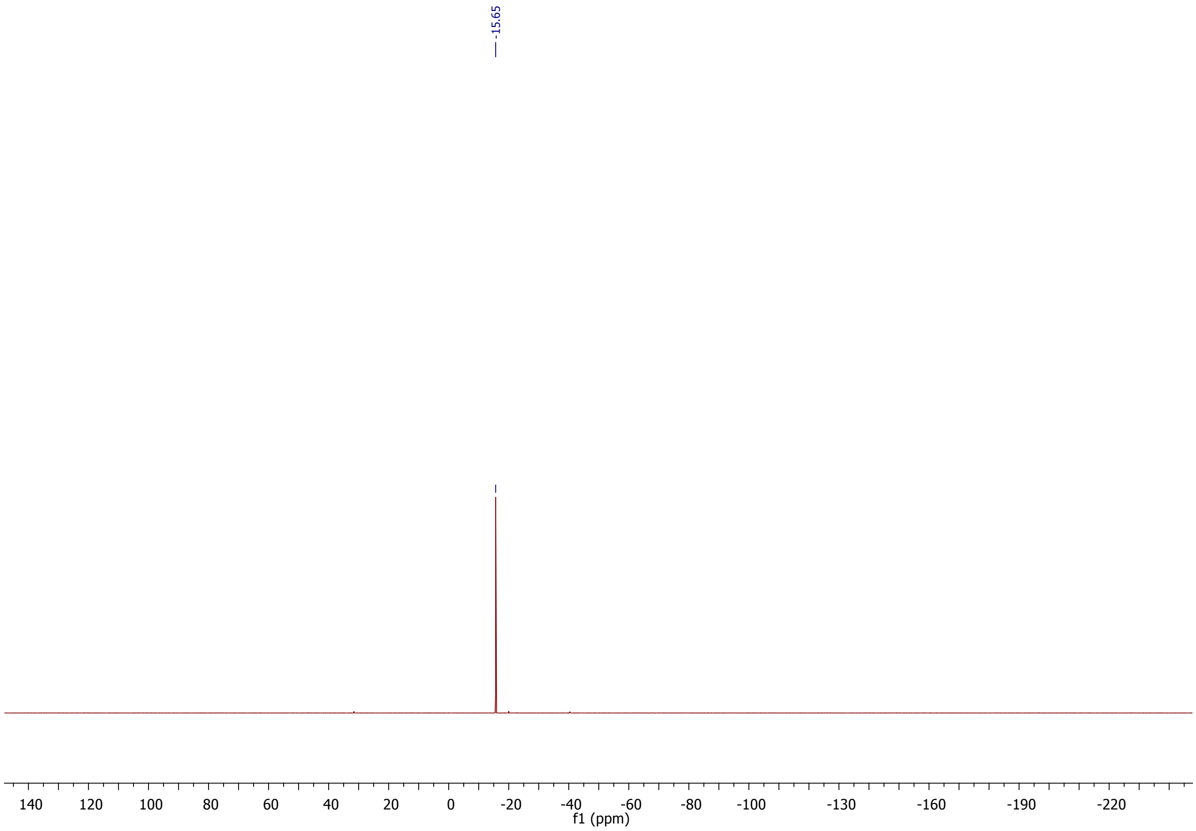

Compound 14

^{1}H NMR (500 MHz) $CDCl_3$

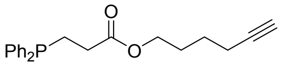

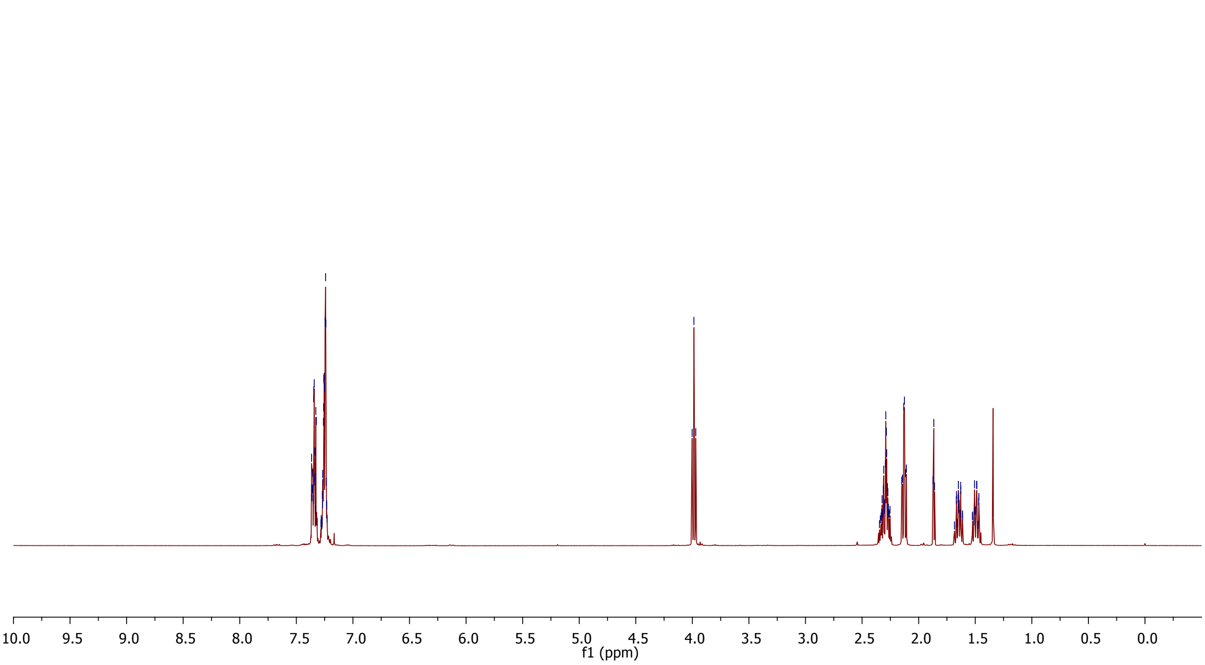

^{13}C NMR (125 MHz) $CDCl_3$

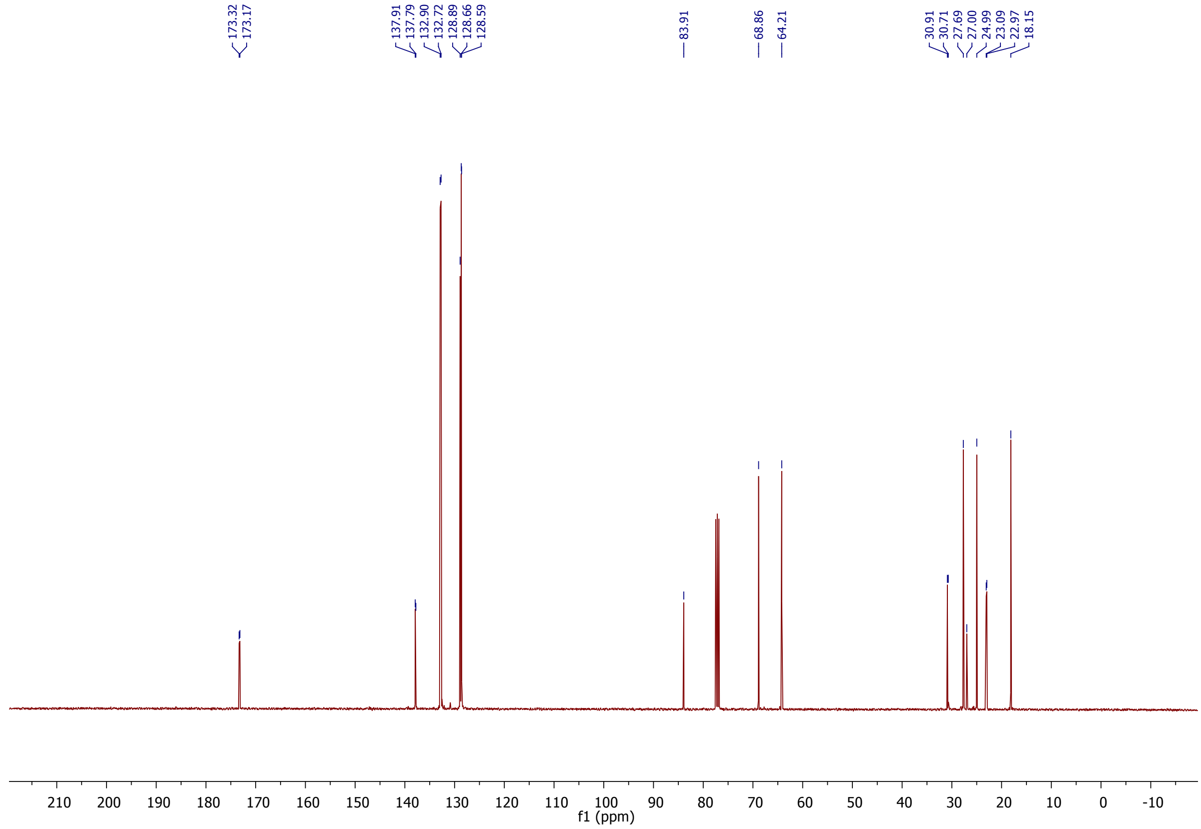

^{31}P NMR (202 MHz) $CDCl_3$

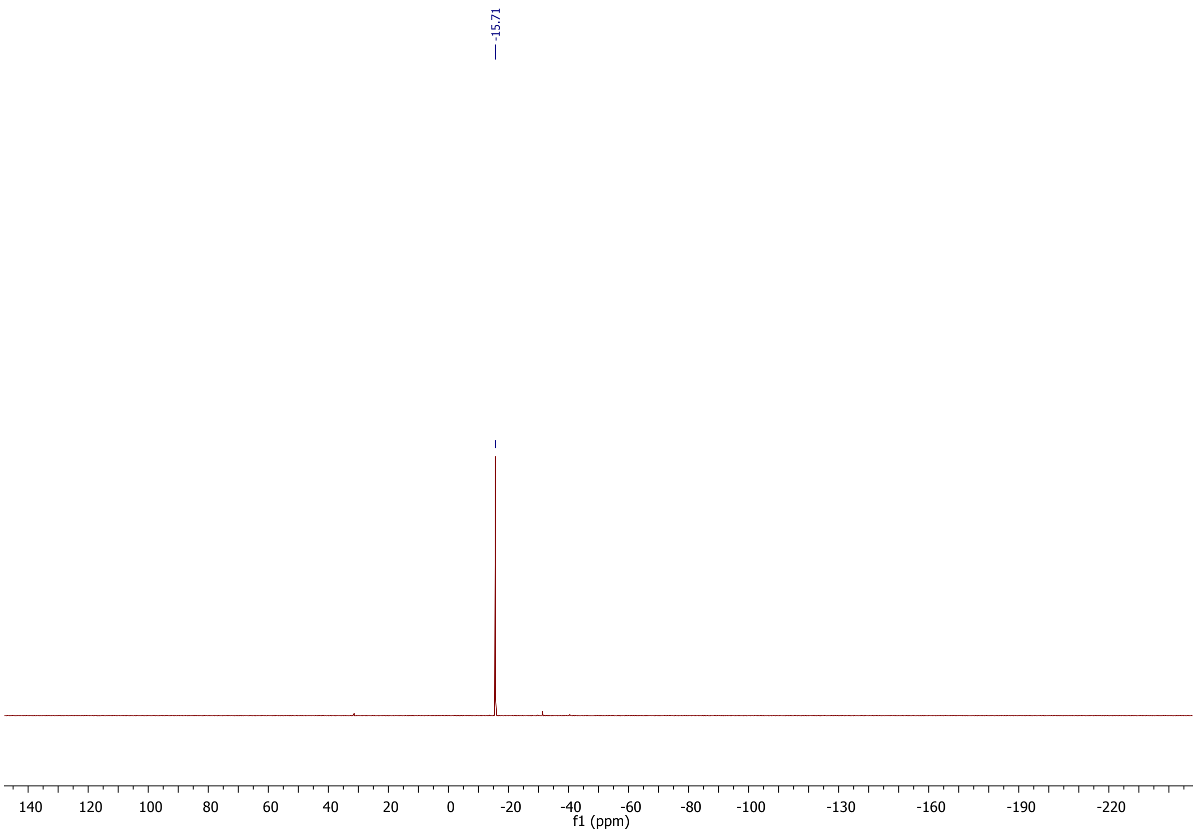

Compound 15

^{1}H NMR (500 MHz) $CDCl_3$

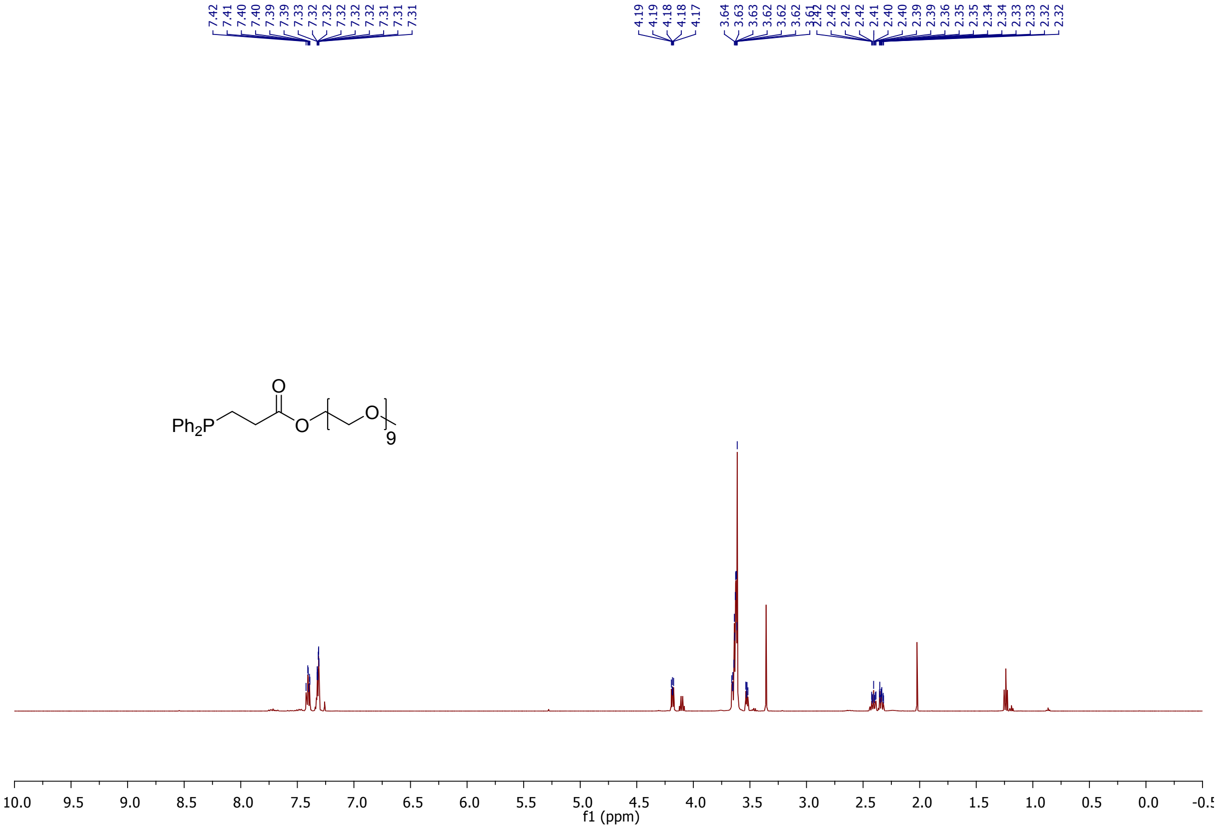

^{13}C NMR (125 MHz) $CDCl_3$

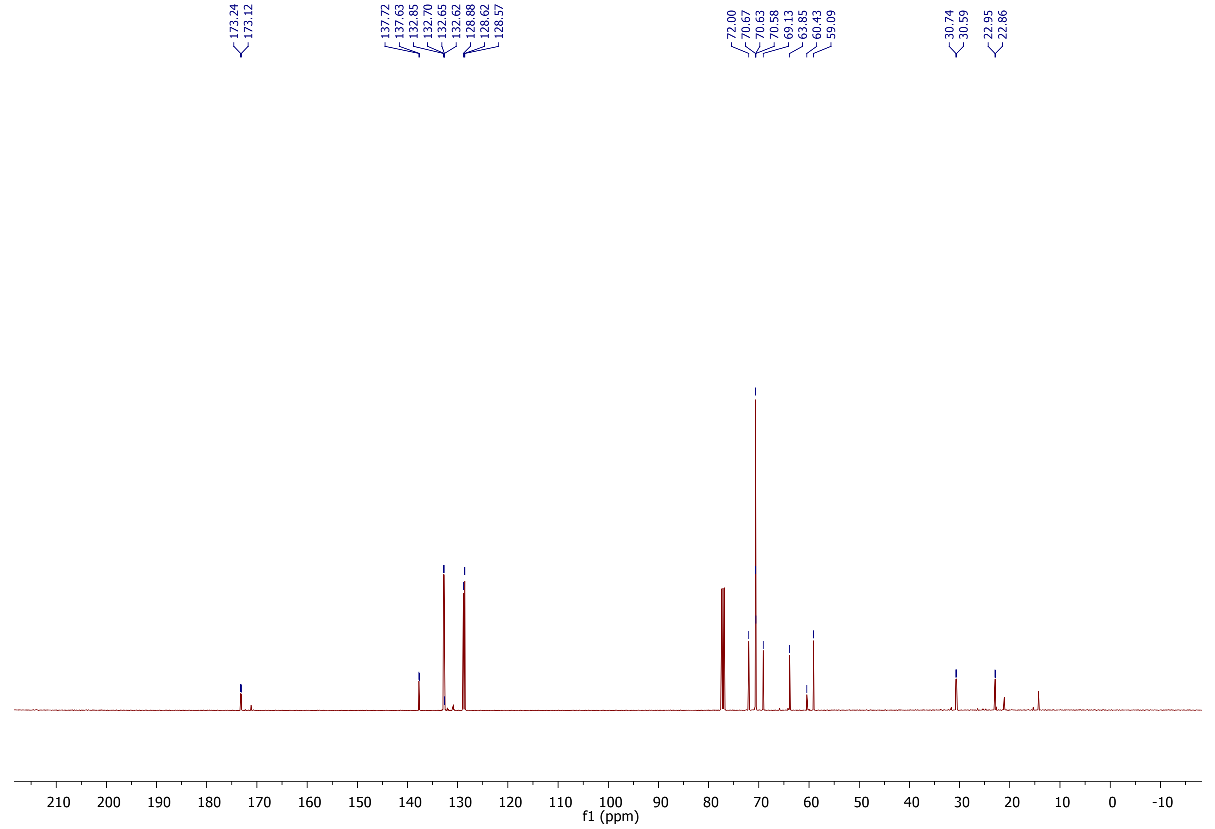

^{31}P NMR (202 MHz) $CDCl_3$

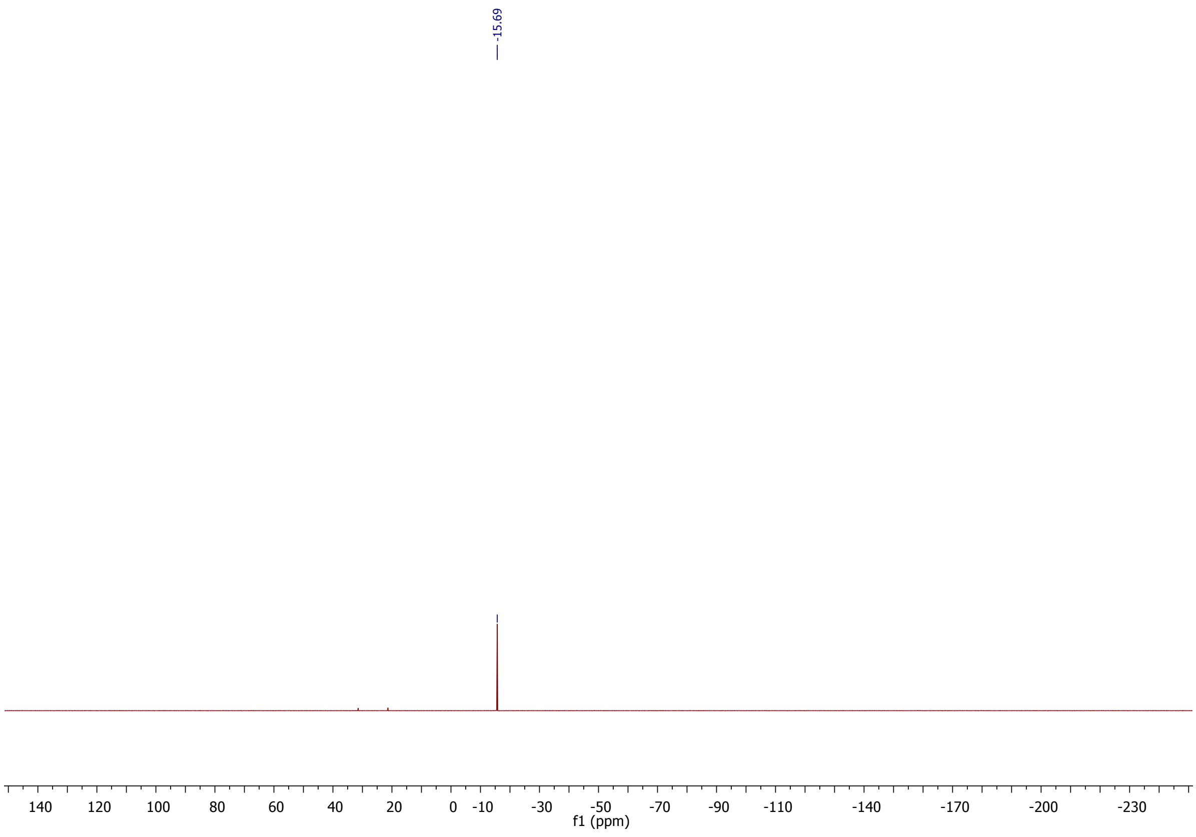

Compound 16

^{1}H NMR (400 MHz) $CDCl_3$

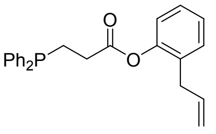

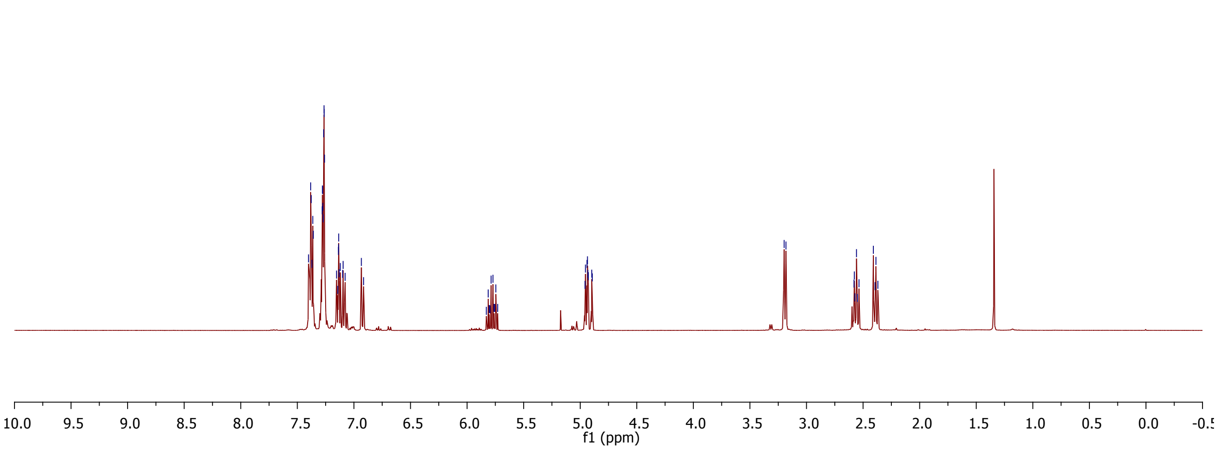

^{13}C NMR (101 MHz) $CDCl_3$

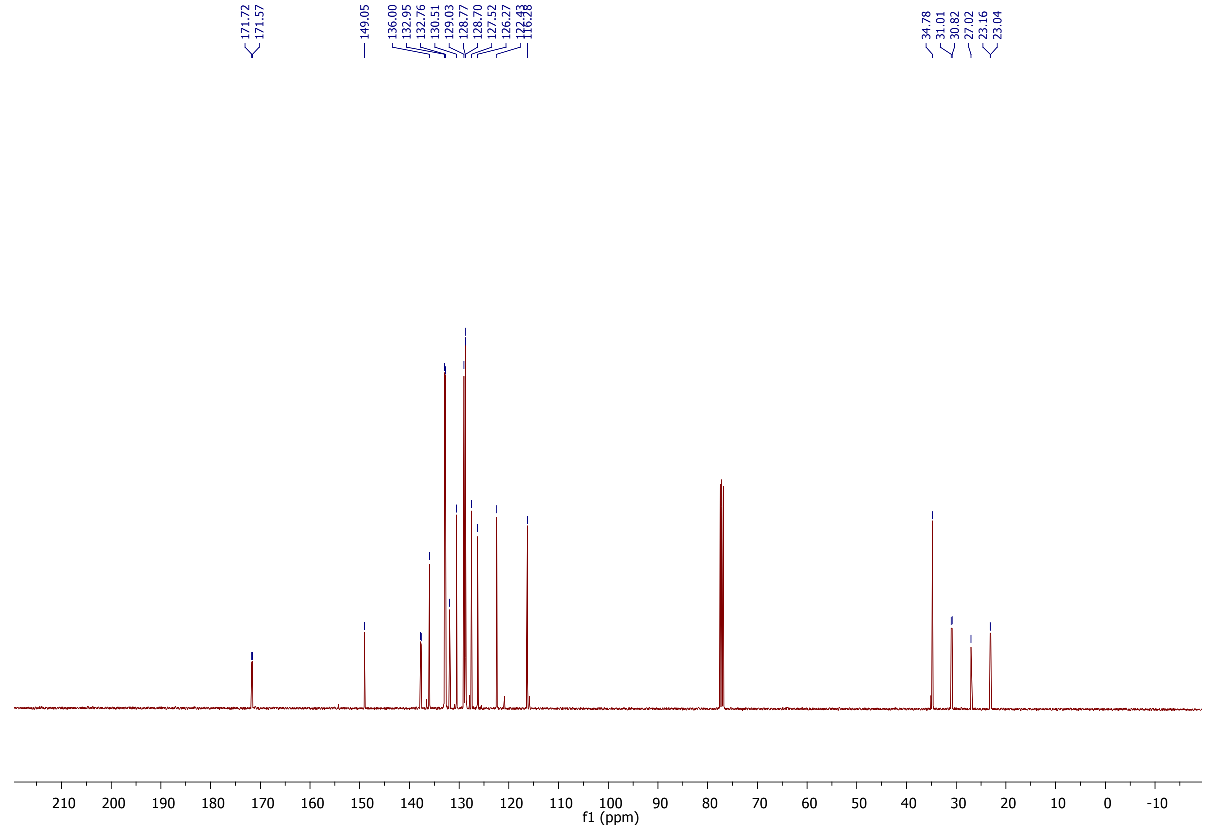

^{31}P NMR (162 MHz) $CDCl_3$

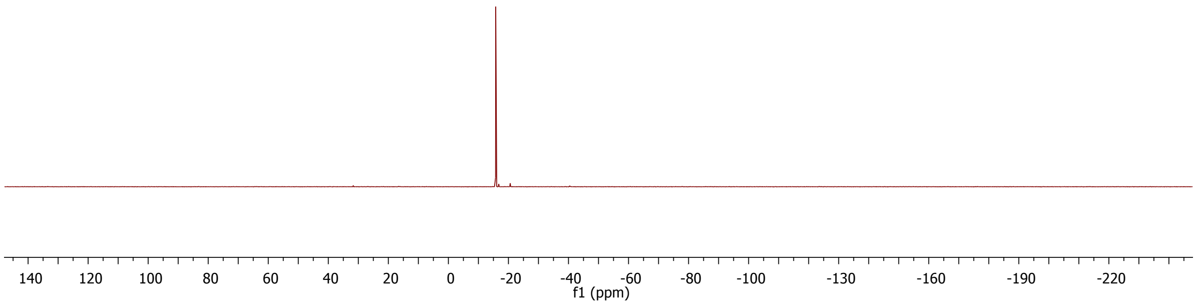

Compound 17

^{1}H NMR (500 MHz) $CDCl_3$

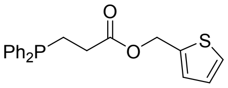

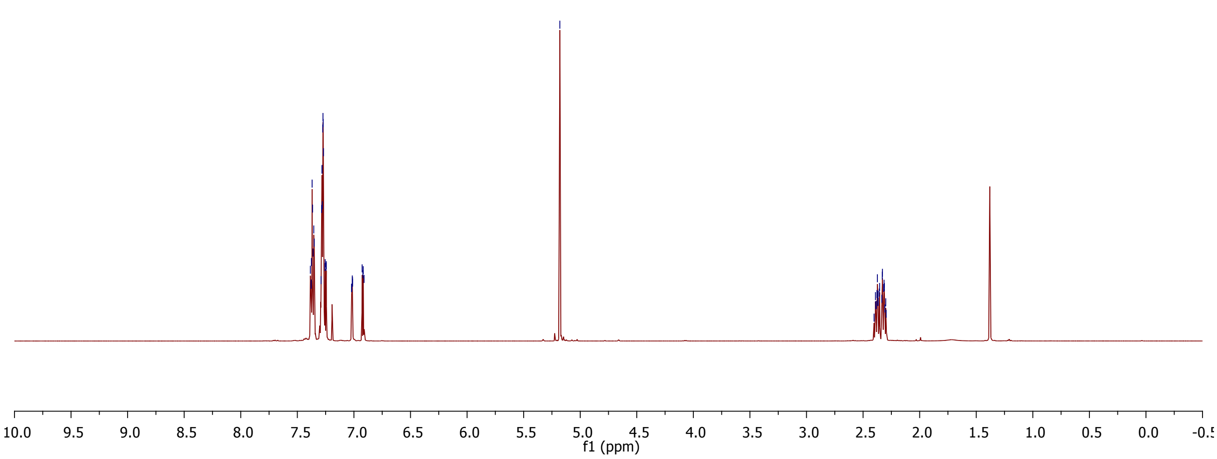

^{13}C NMR (125 MHz) $CDCl_3$

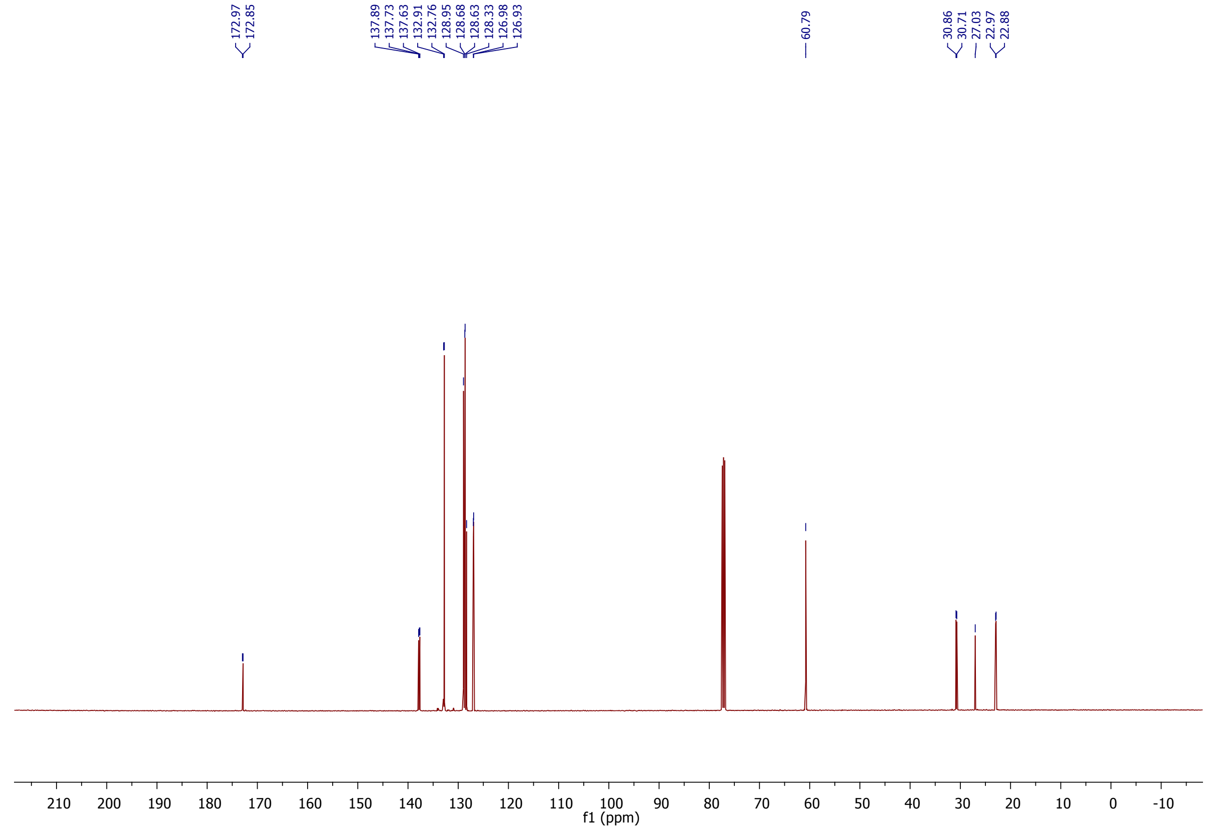

^{31}P NMR (202 MHz) $CDCl_3$

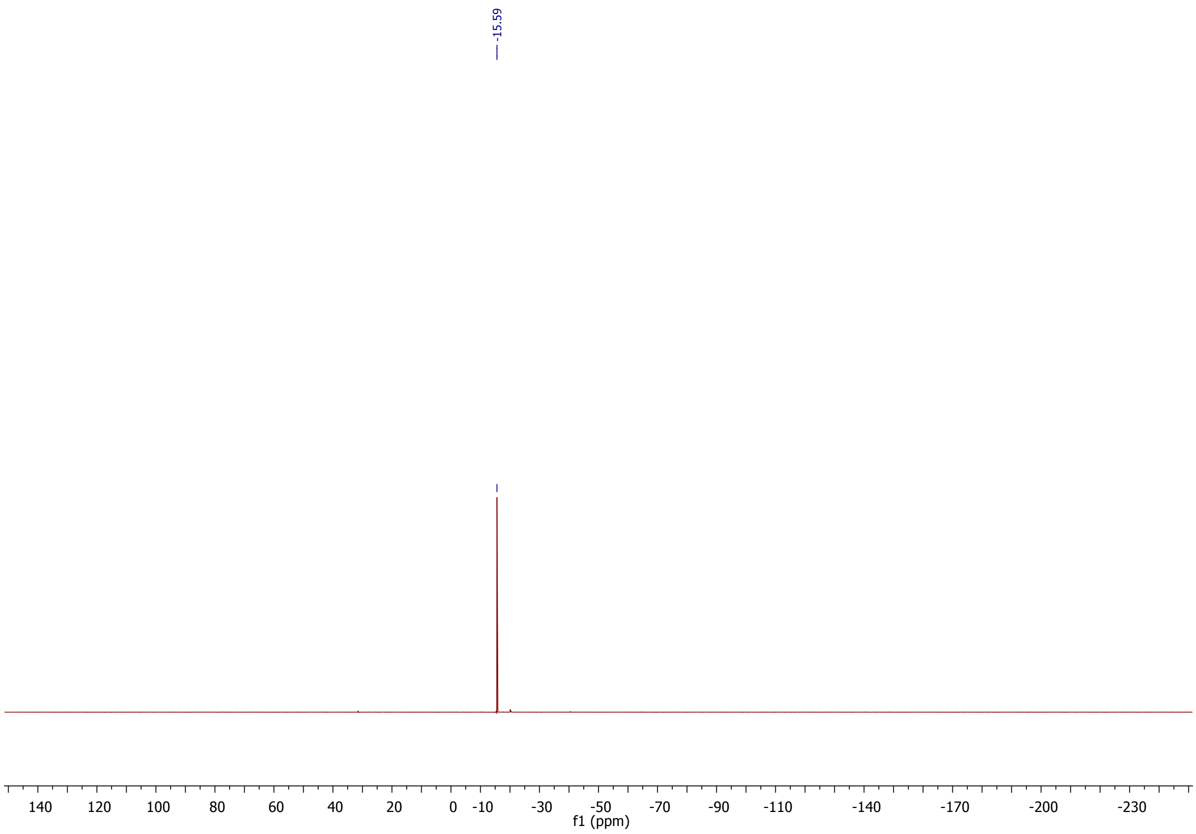

Compound 18

^{1}H NMR (400 MHz) $CDCl_3$

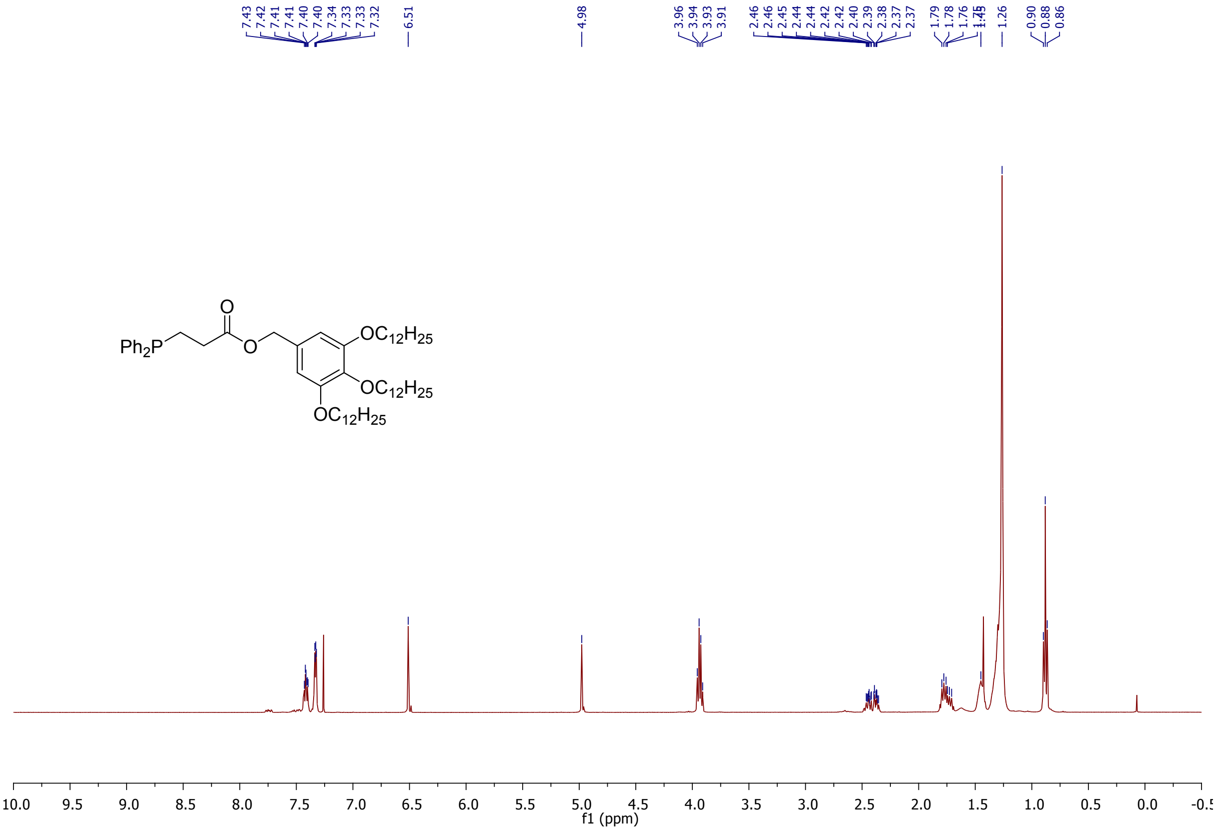

^{13}C NMR (101 MHz) $CDCl_3$

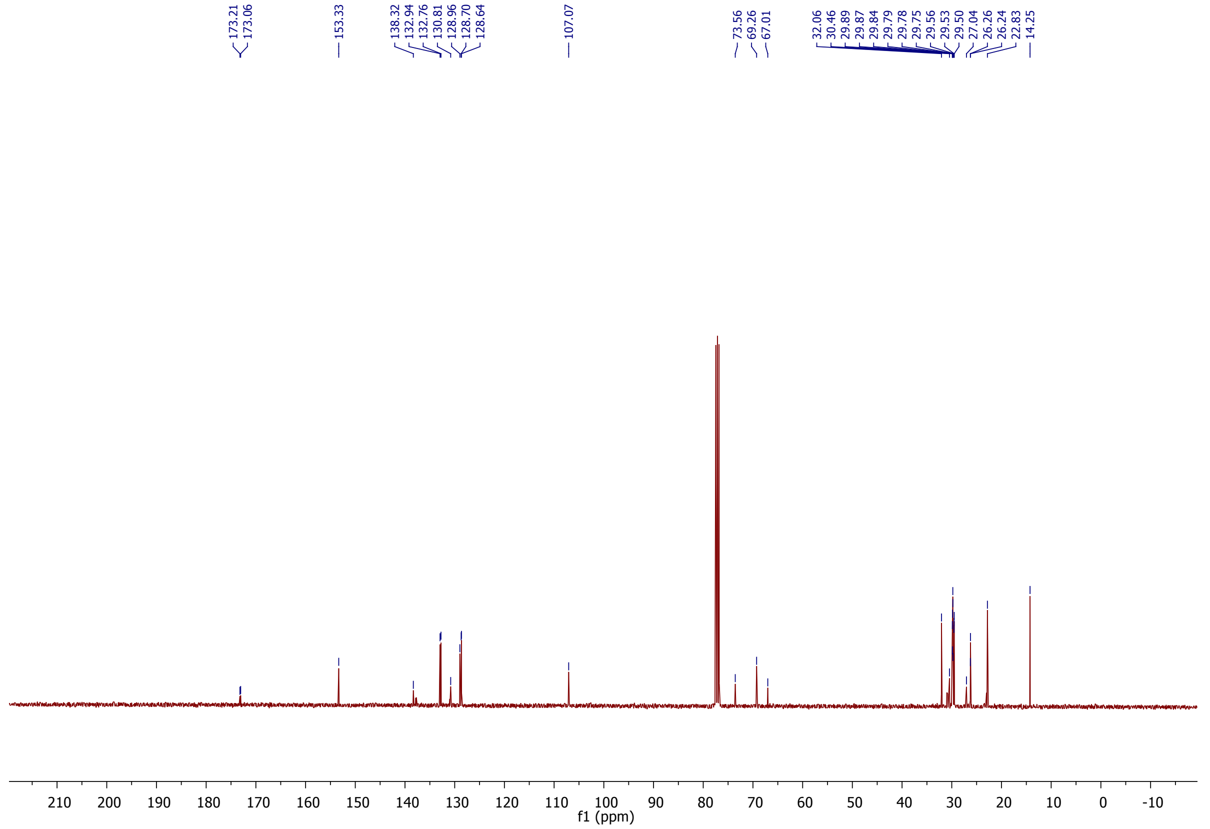

^{31}P NMR (162 MHz) $CDCl_3$

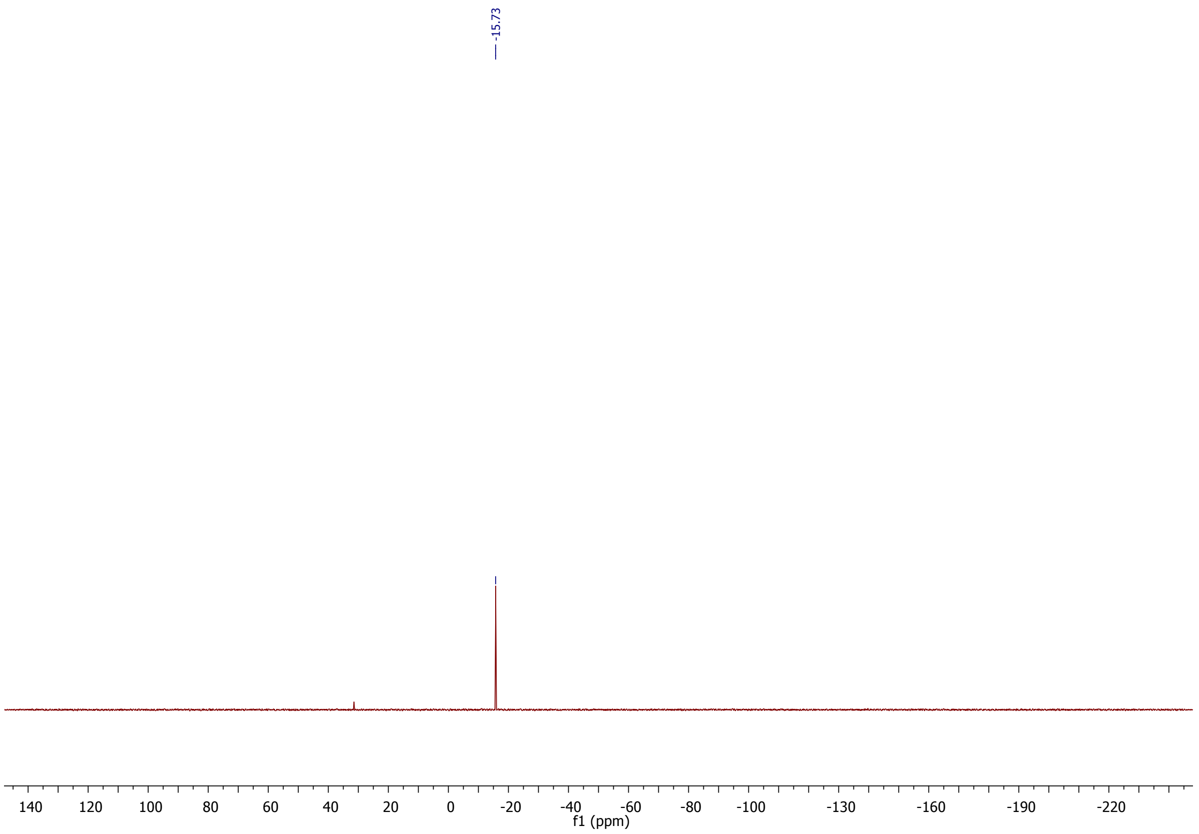

4.2 Monomethacrylate derivatives

Compound 19

^{1}H NMR (400 MHz) $CDCl_3$

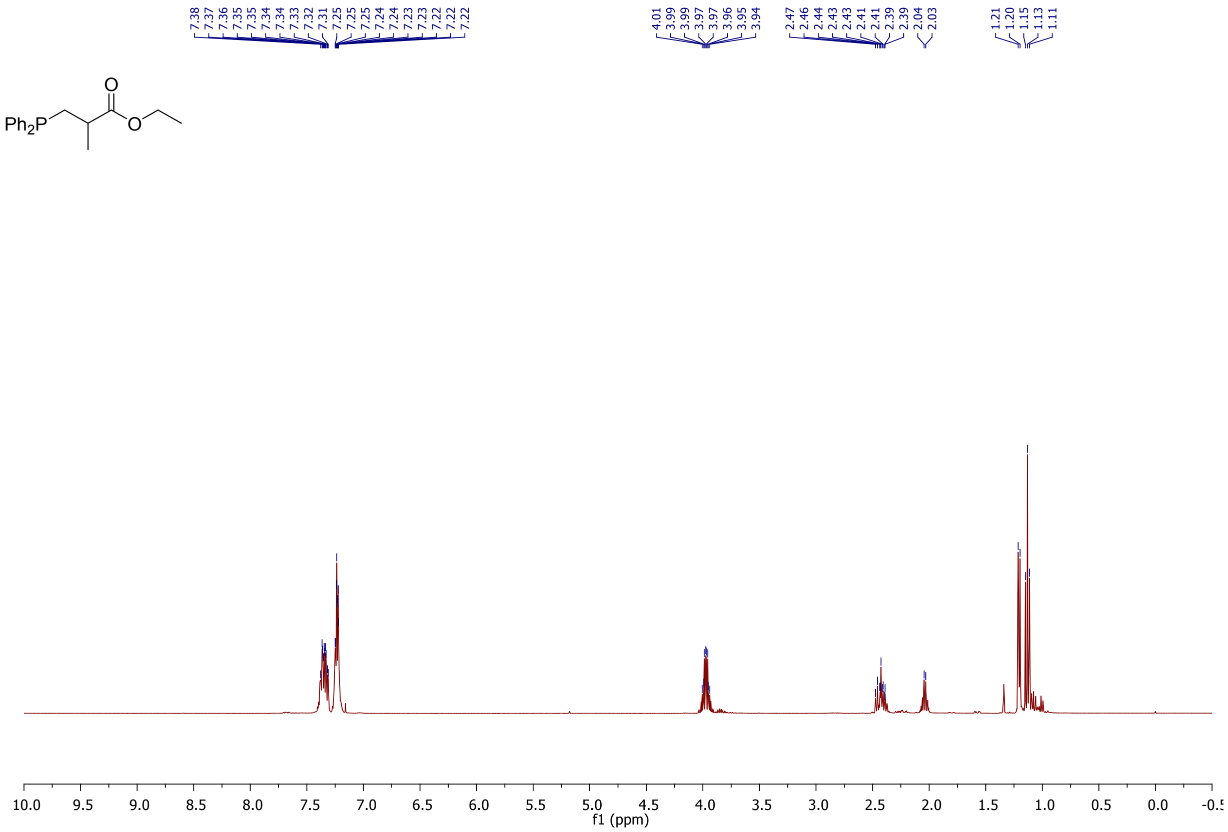

^{13}C NMR (101 MHz) $CDCl_3$

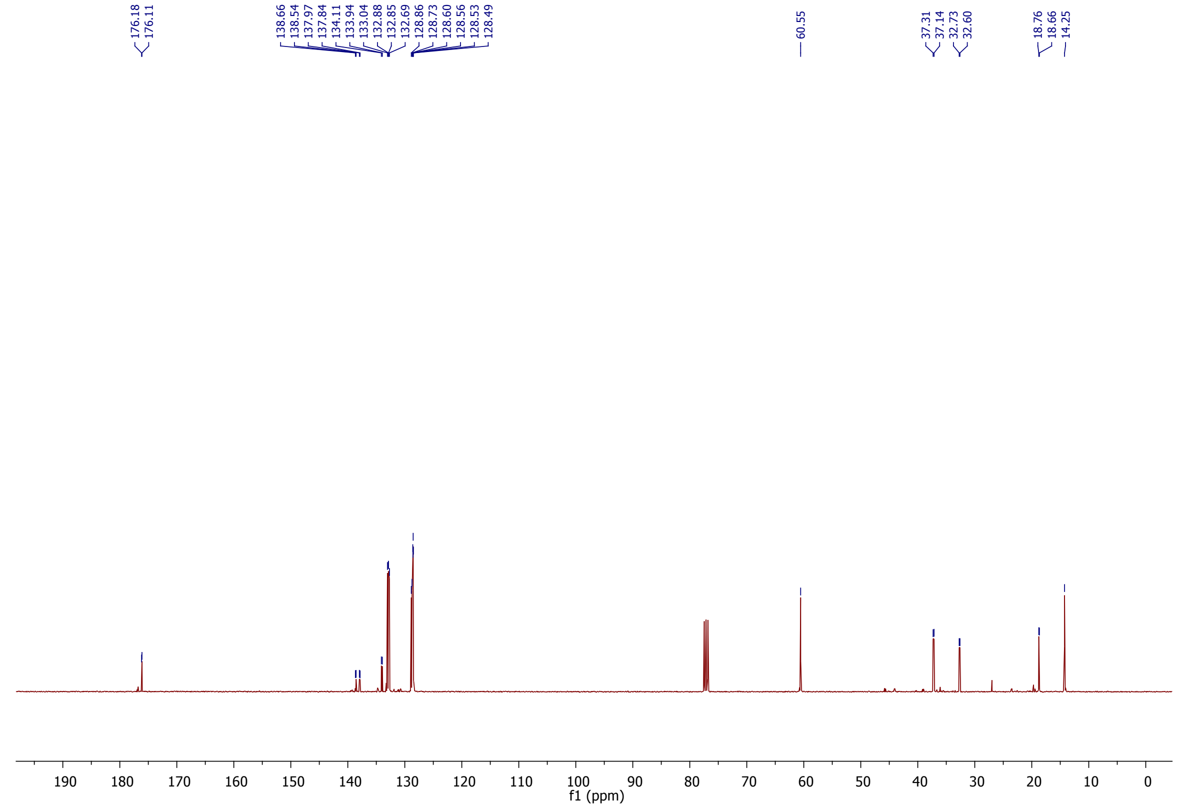

^{31}P NMR (162 MHz) $CDCl_3$

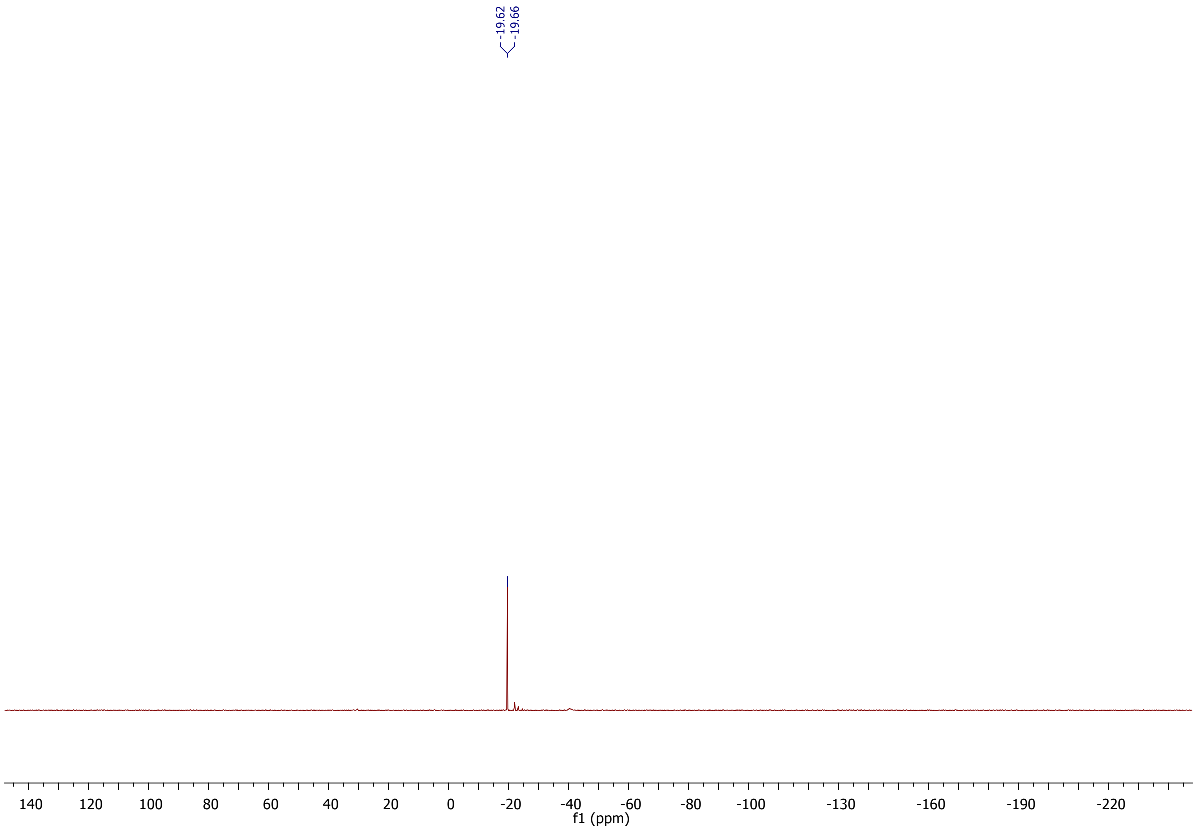

Compound 20

^{1}H NMR (400 MHz) $CDCl_3$

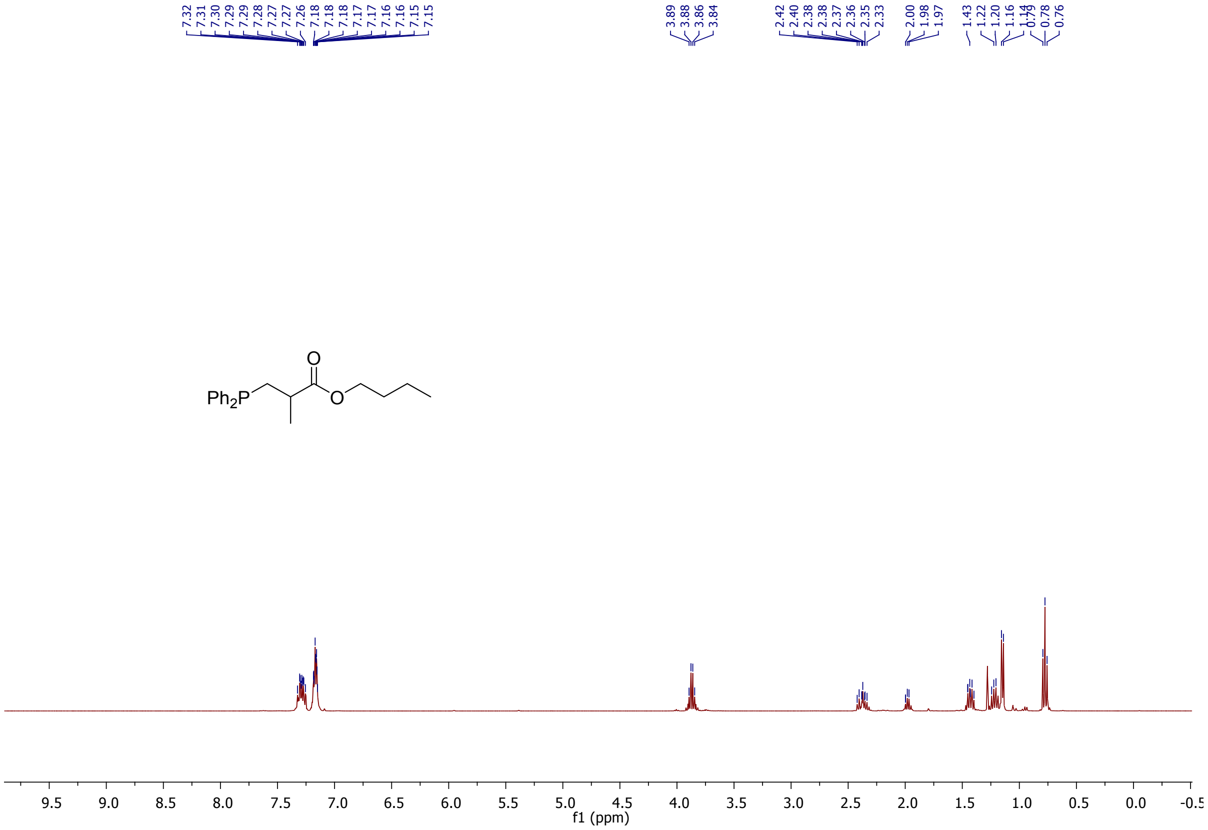

^{13}C NMR (101 MHz) $CDCl_3$

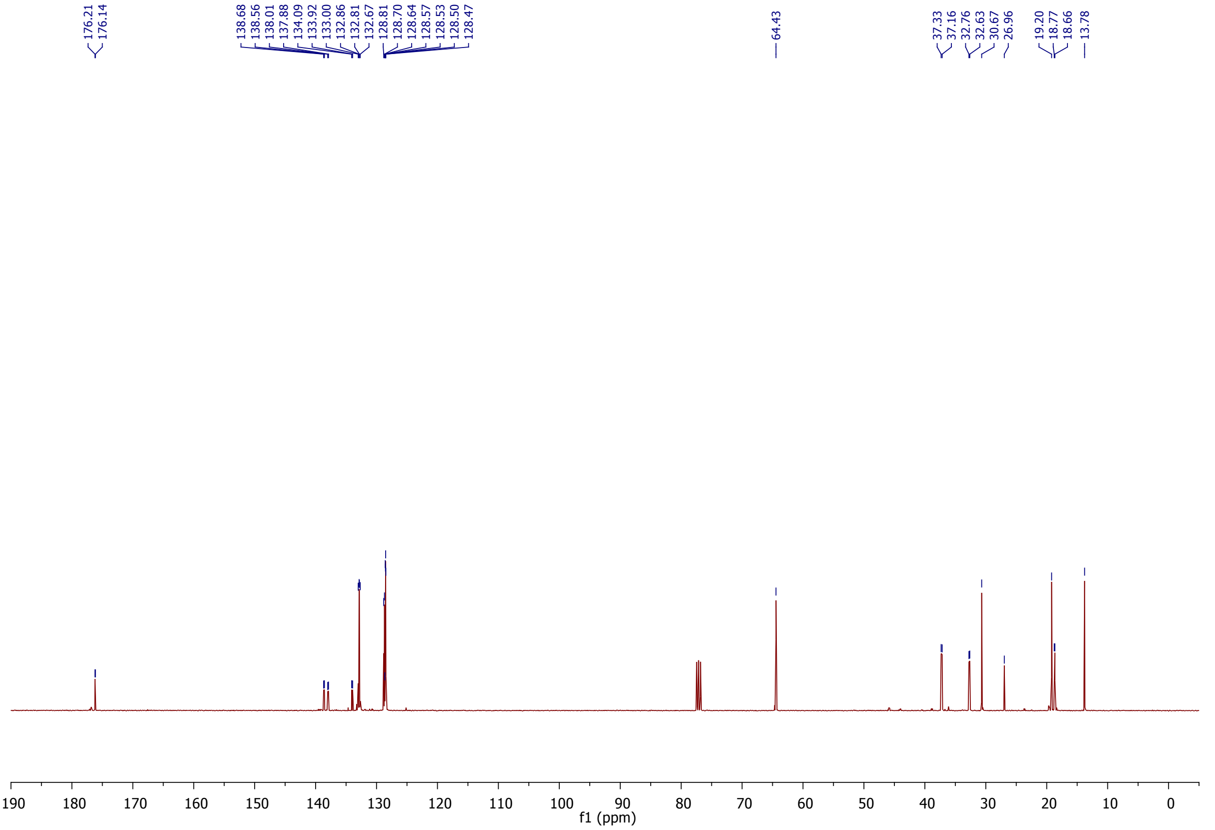

^{31}P NMR (162 MHz) $CDCl_3$

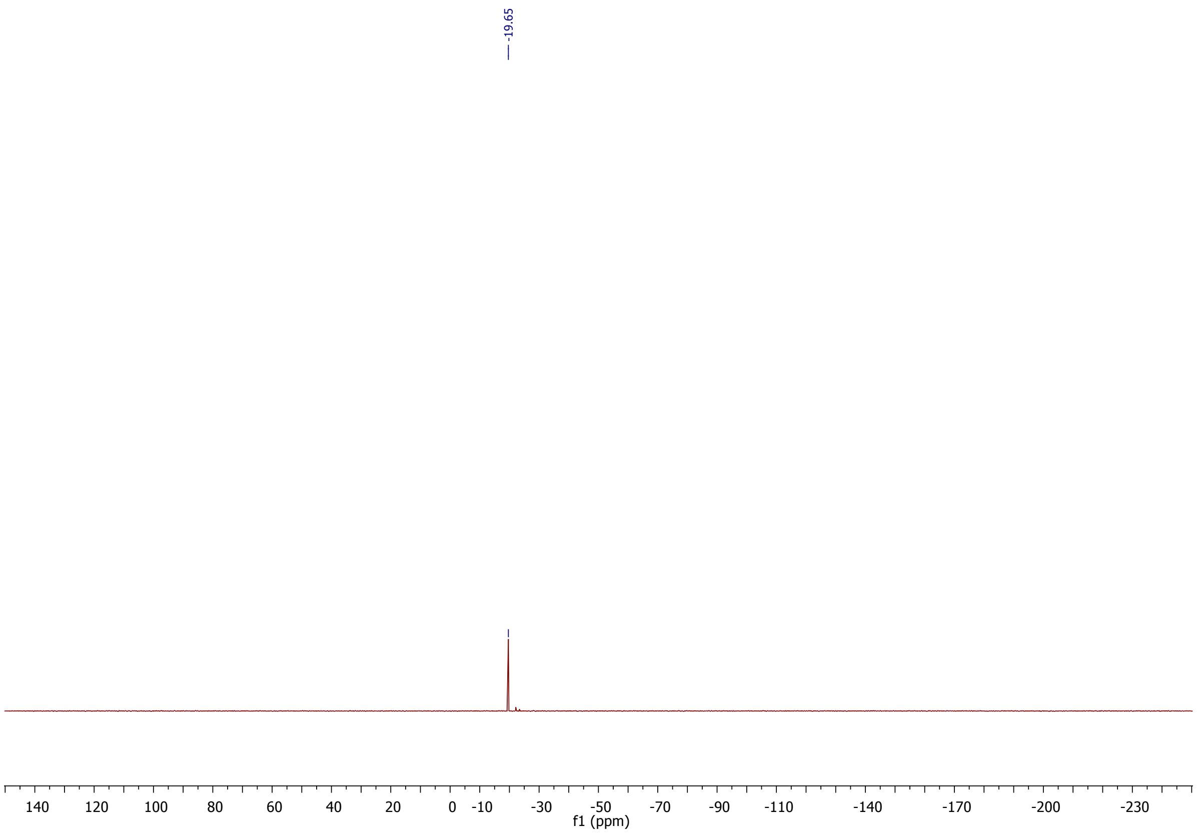

Compound 21

^{1}H NMR (400 MHz) $CDCl_3$

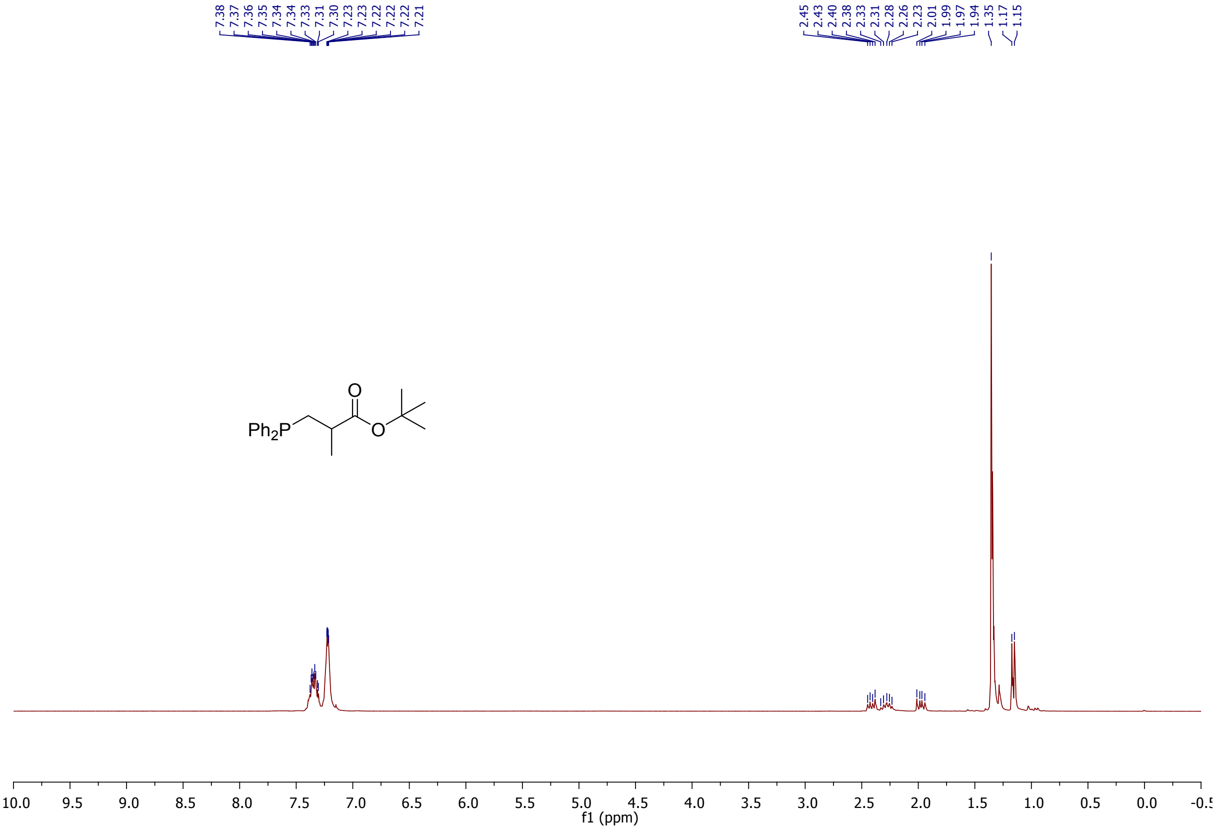

^{13}C NMR (101 MHz) $CDCl_3$

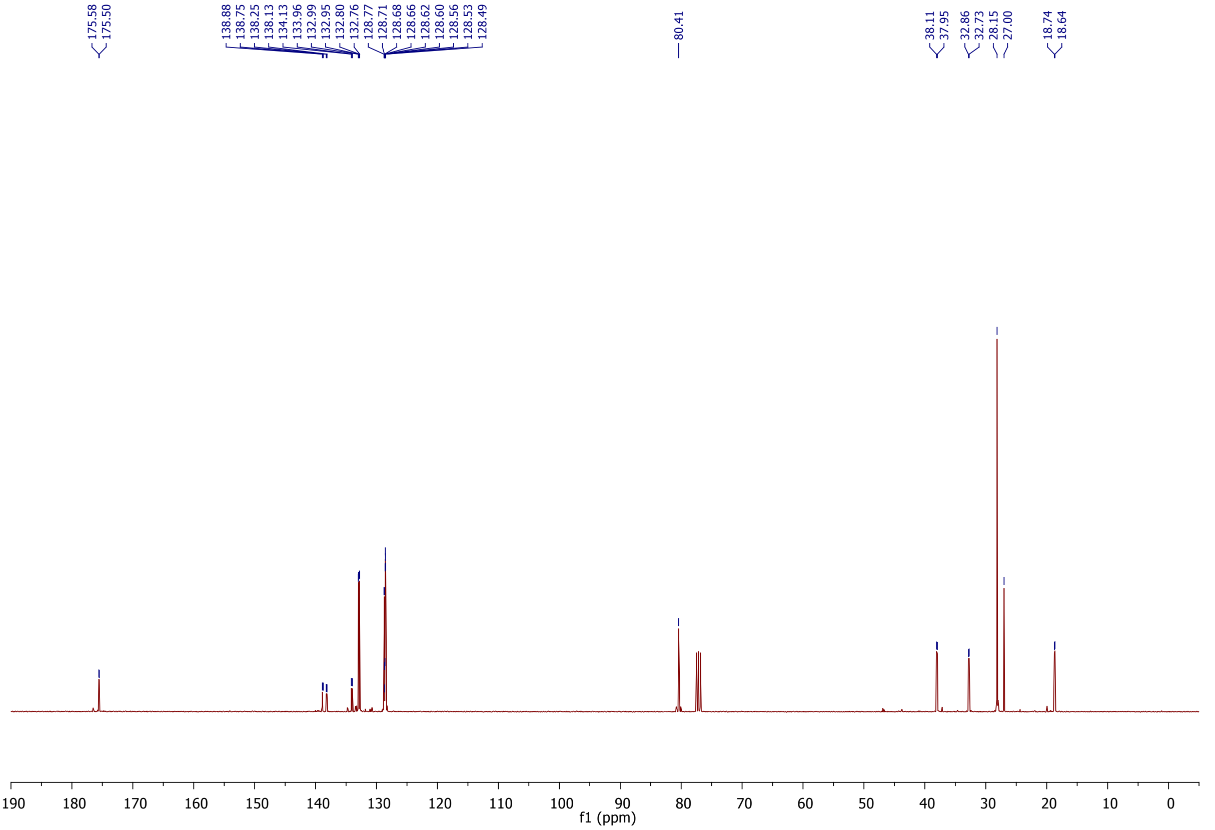

^{31}P NMR (162 MHz) $CDCl_3$

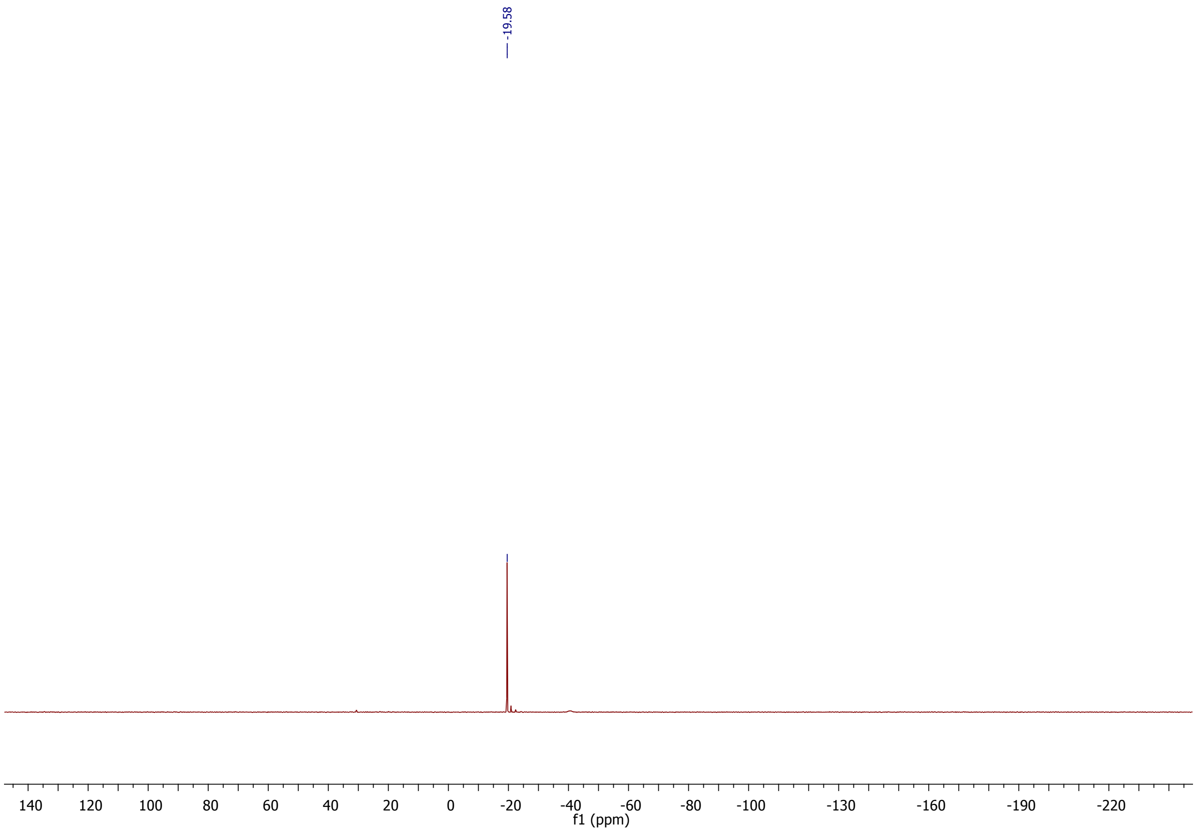
-19.58
140
120
100
80
60
40
20
0
-20
-40
-60
-80
-100
-130
-160
-190
-220
f1 (ppm)

Compound 22

^{1}H NMR (400 MHz) $CDCl_3$

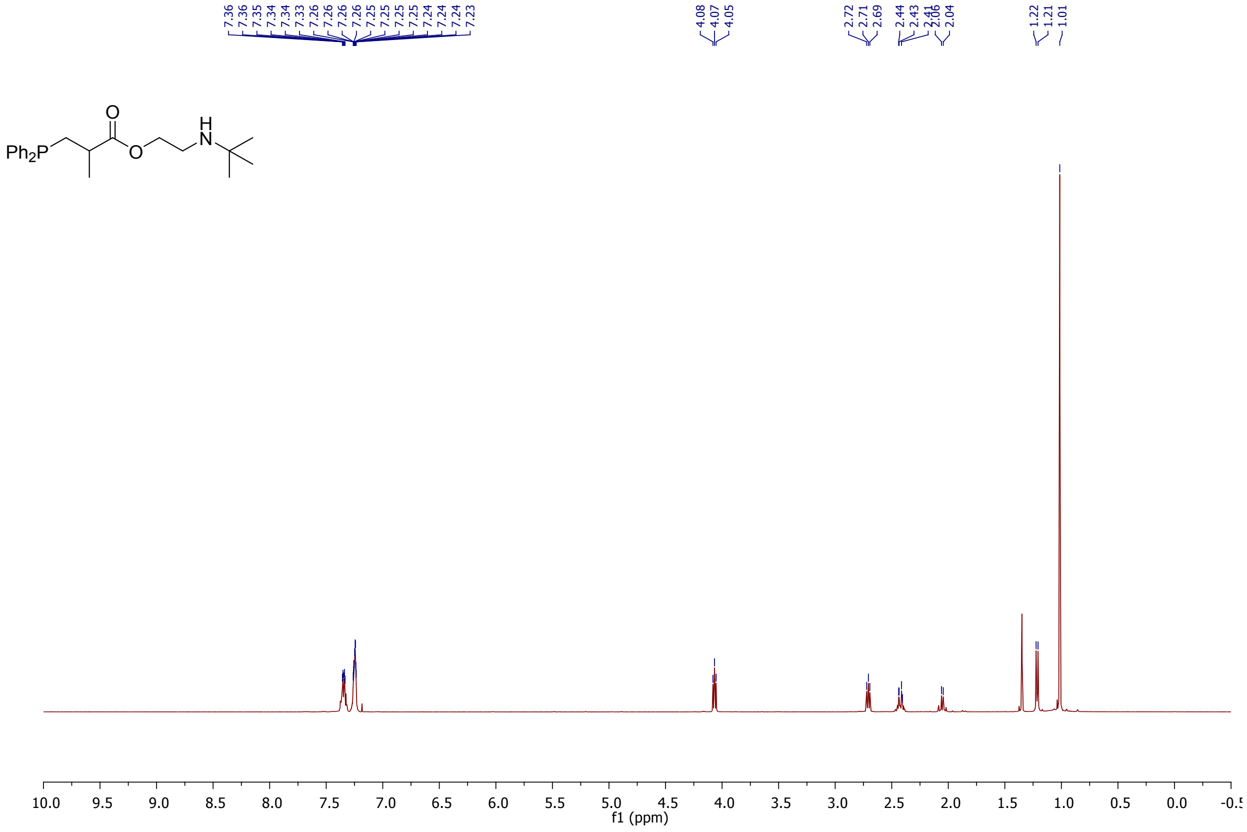

^{13}C NMR (101 MHz) $CDCl_3$

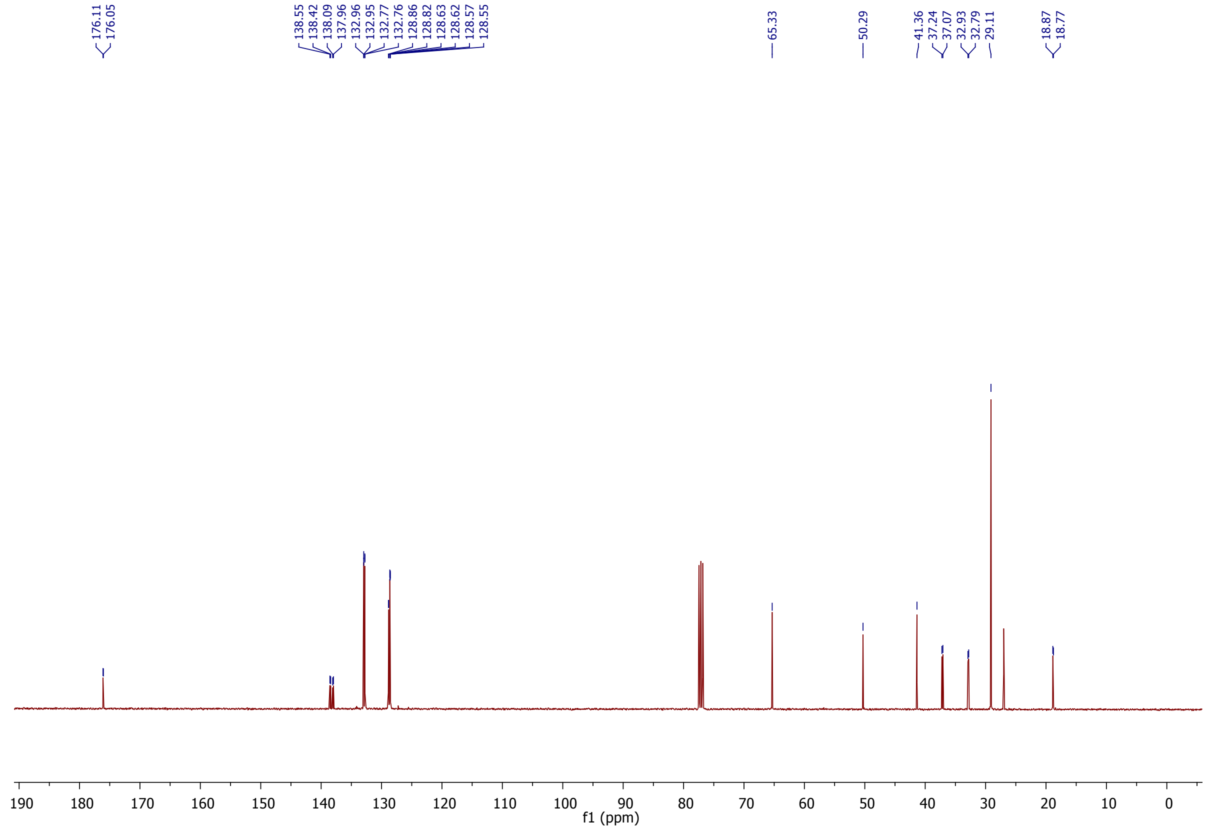

^{31}P NMR (162 MHz) $CDCl_3$

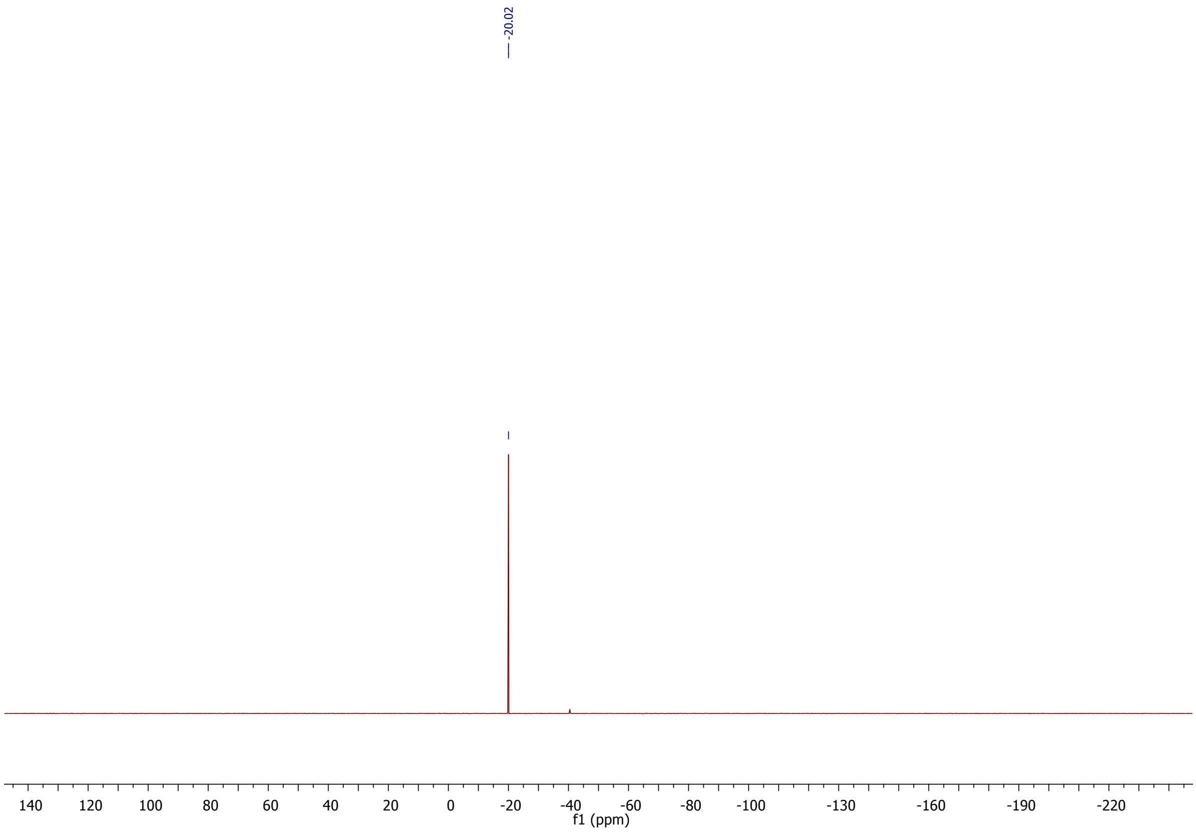

-20.02
140
120
100
80
60
40
20
0
-20
-40
-60
-80
-100
-130
-160
-190
-220
f1 (ppm)

Compound 23

^{1}H NMR (400 MHz) $CDCl_3$

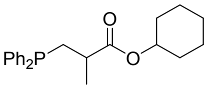

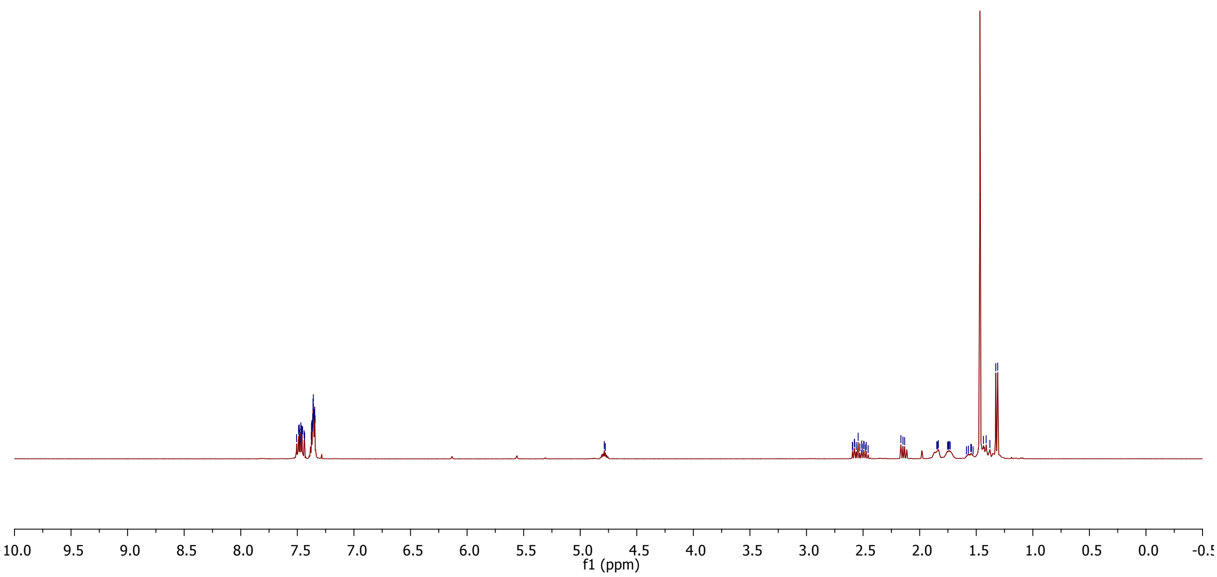

^{13}C NMR (101 MHz) $CDCl_3$

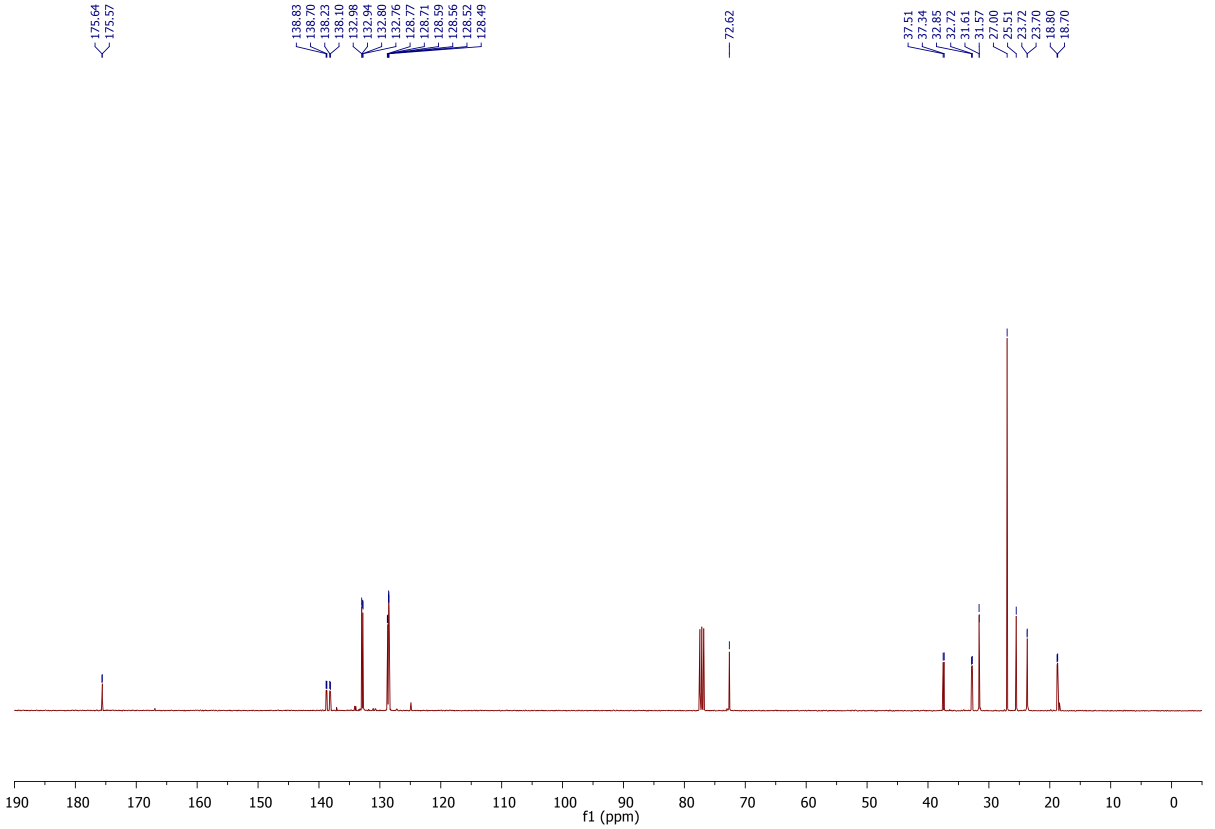

^{31}P NMR (162 MHz) $CDCl_3$

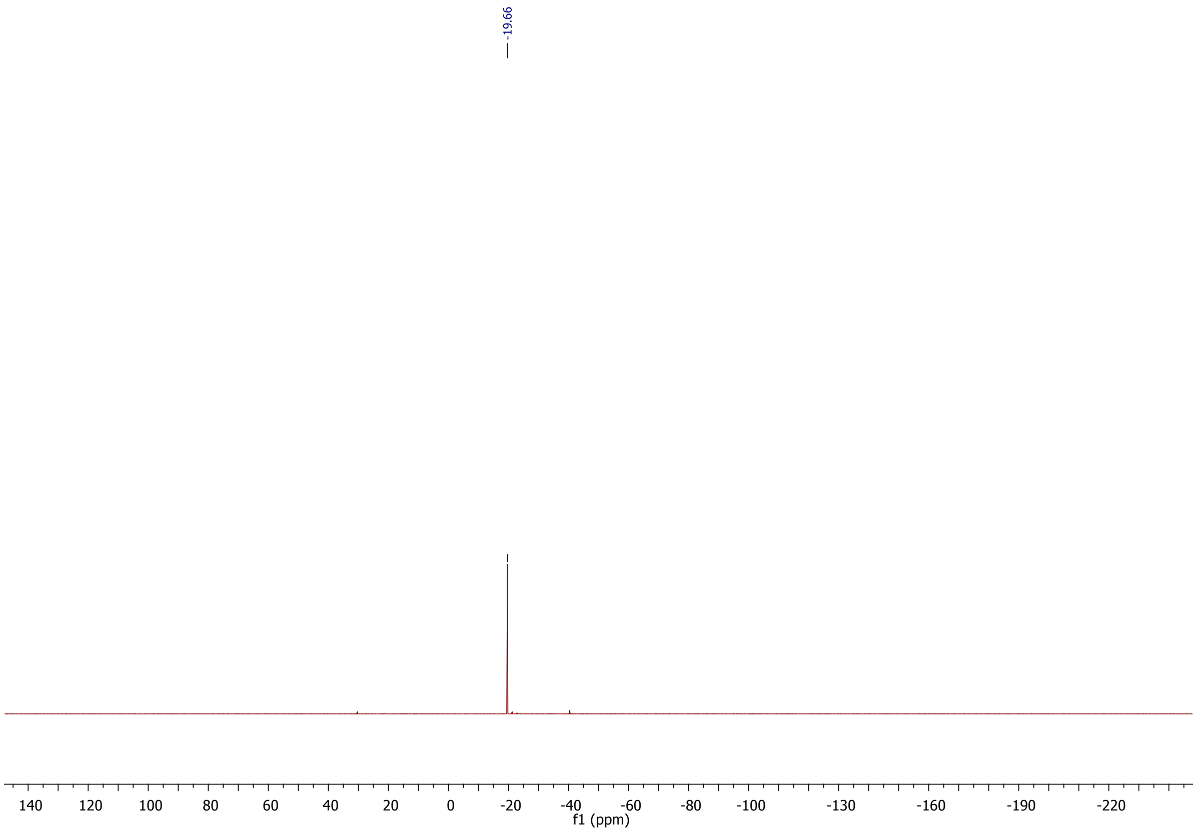

-19.66
140
120
100
80
60
40
20
0
-20
-40
-60
-80
-100
-130
-160
-190
-220
f1 (ppm)

Compound 24

^{1}H NMR (400 MHz) $CDCl_3$

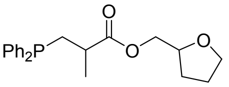

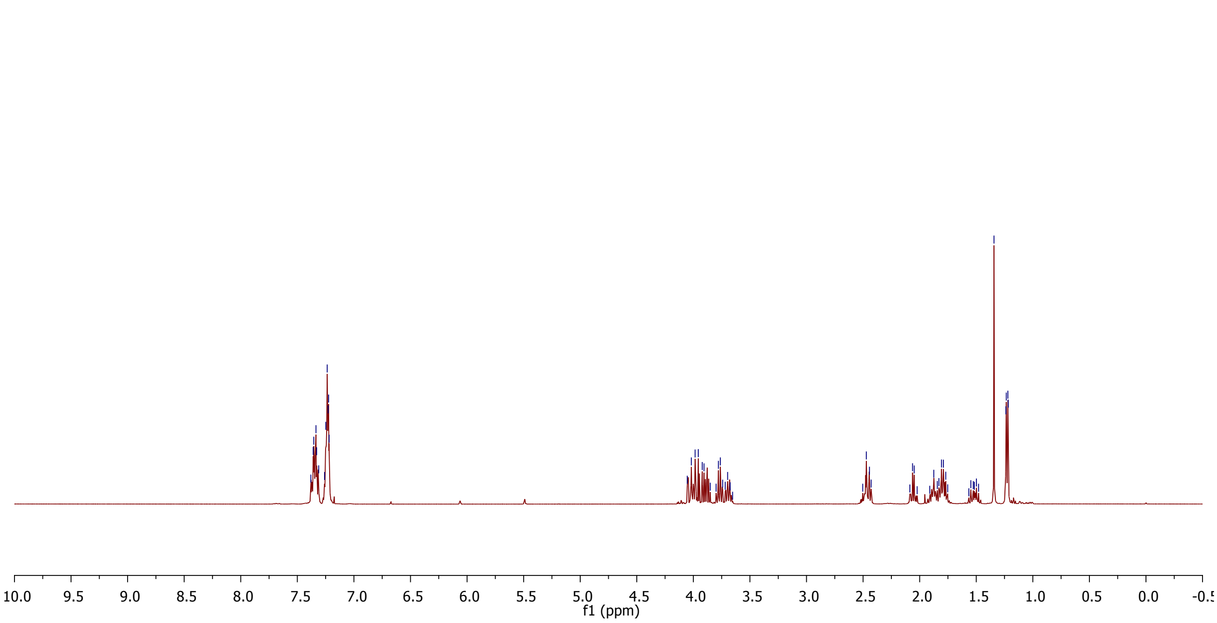

^{13}C NMR (101 MHz) $CDCl_3$

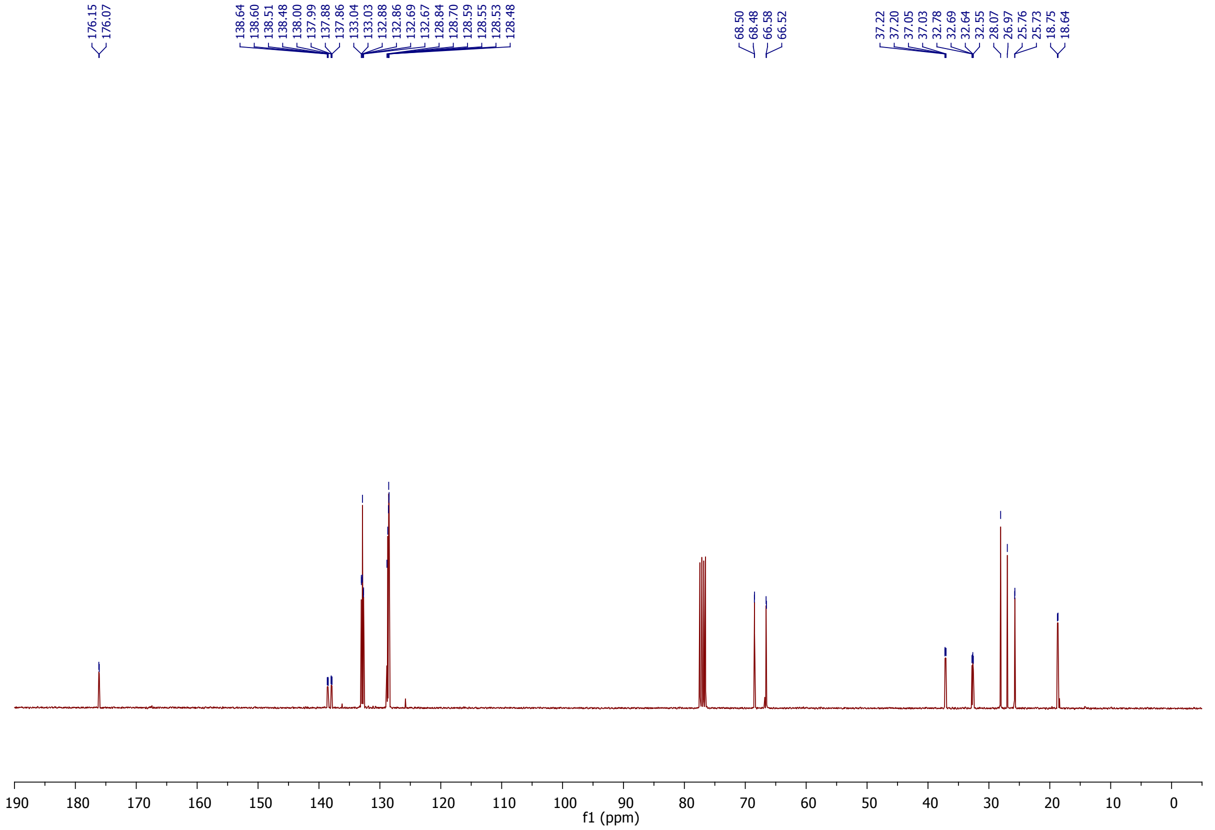

^{31}P NMR (162 MHz) $CDCl_3$

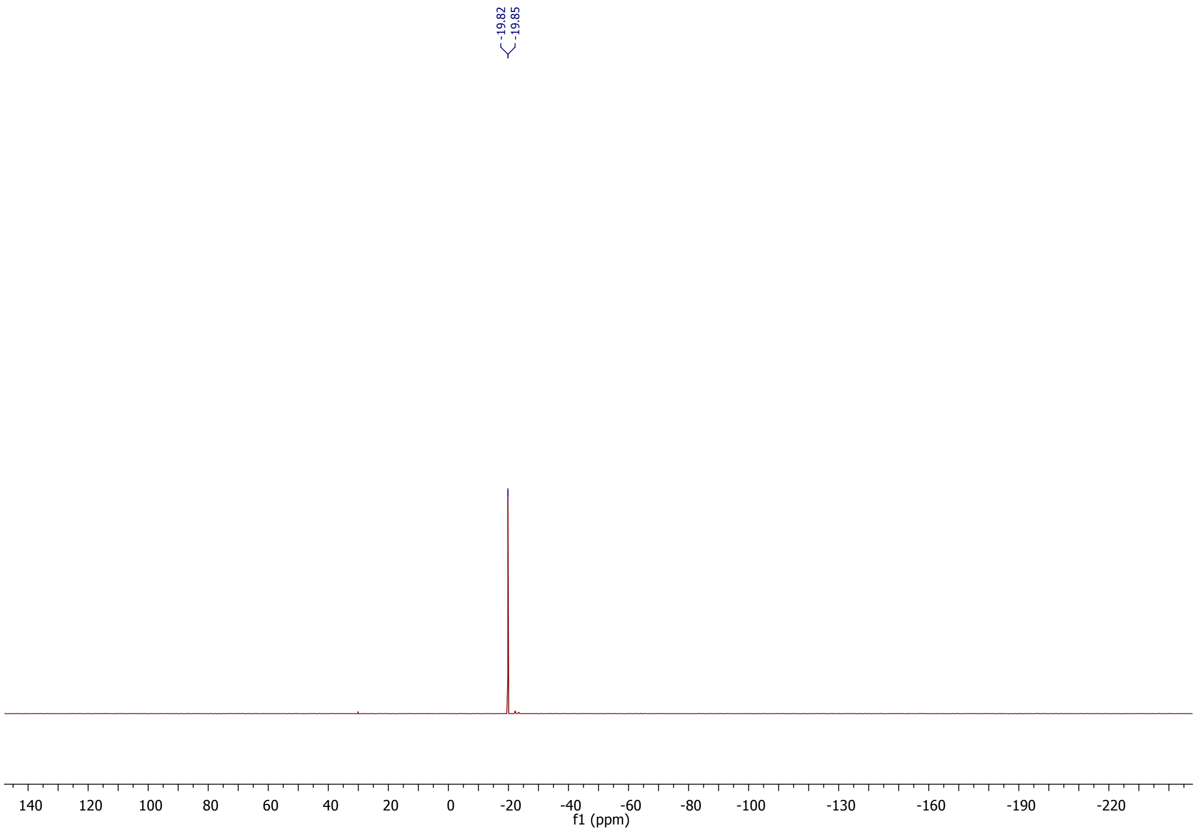

-19.82
-19.85
140
120
100
80
60
40
20
0
-20
-40
-60
-80
-100
-130
-160
-190
-220
f1 (ppm)

Compound 25

^{1}H NMR (300 MHz) $CDCl_3$

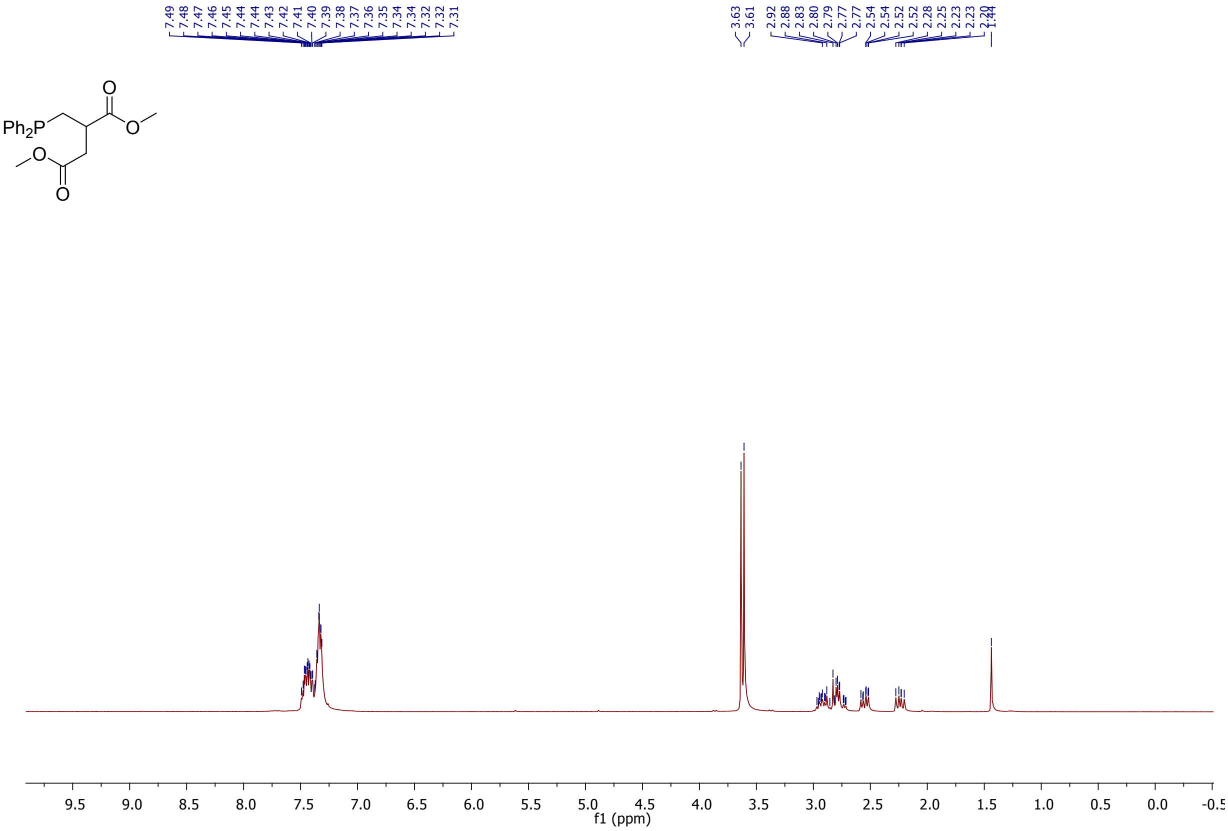

^{13}C NMR (75 MHz) $CDCl_3$

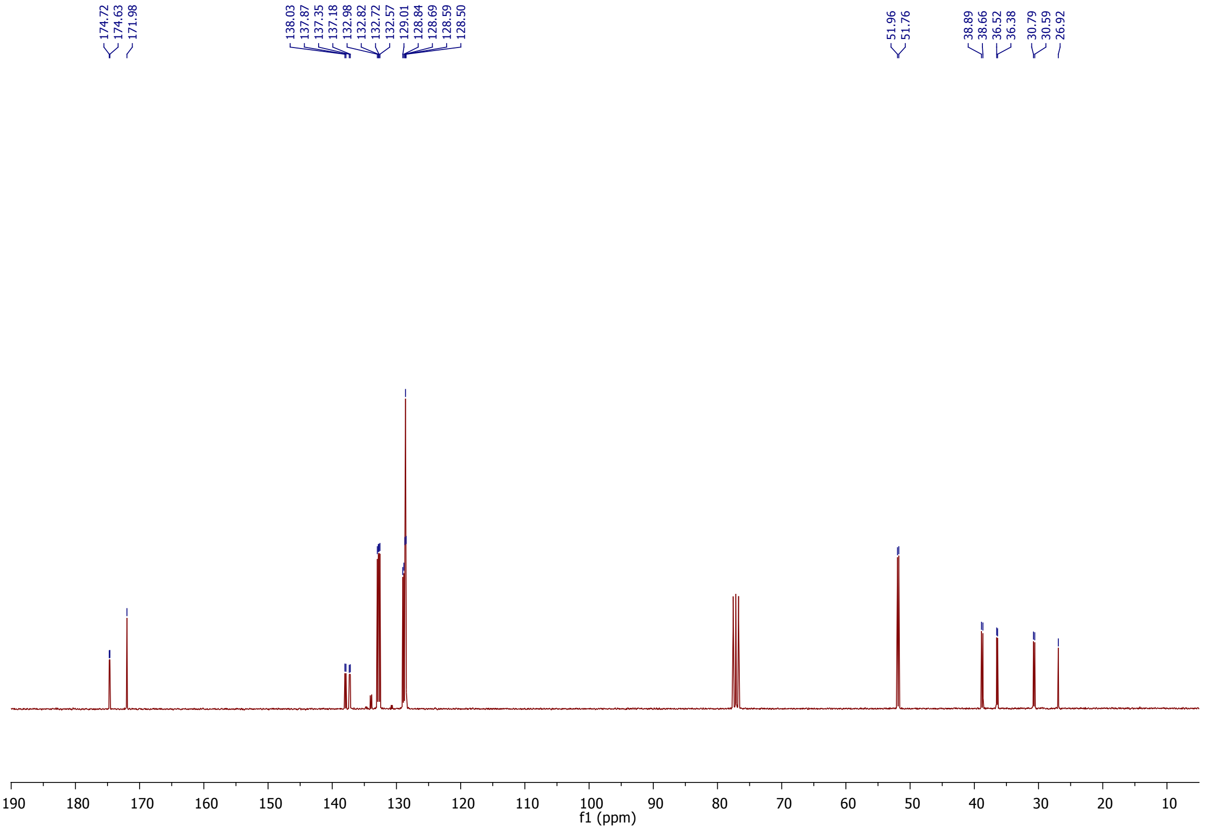

^{31}P NMR (121 MHz) $CDCl_3$

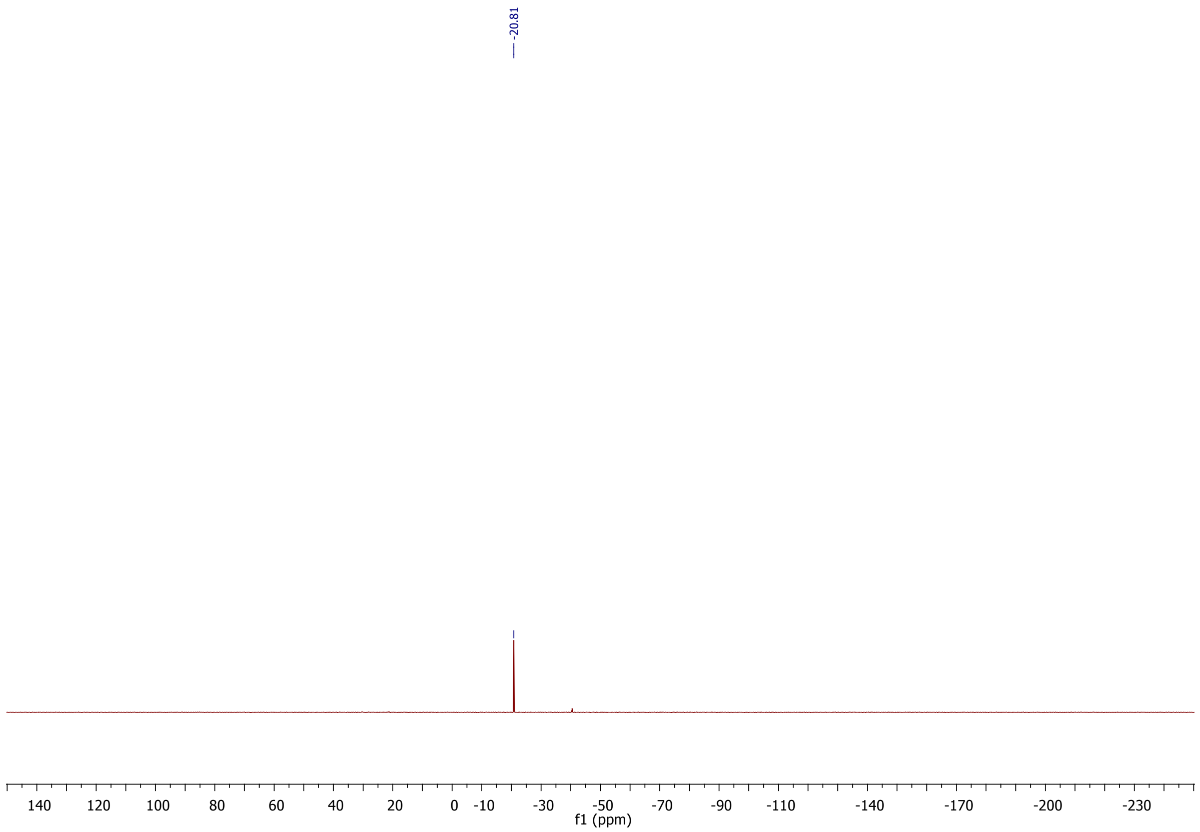
-20.81
140
120
100
80
60
40
20
0
-10
-30
-50
-70
-90
-110
-140
-170
-200
-230
f1 (ppm)

Compound 26

^{1}H NMR (500 MHz) $CDCl_3$

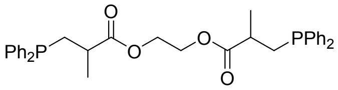

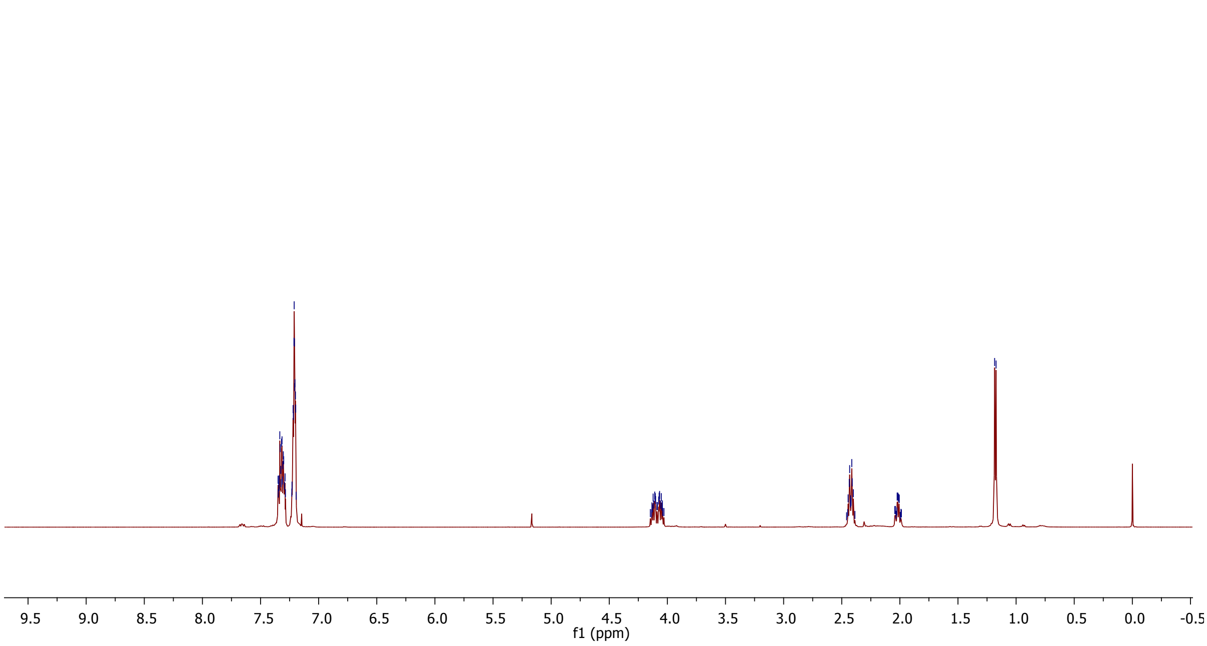

^{13}C NMR (125 MHz) $CDCl_3$

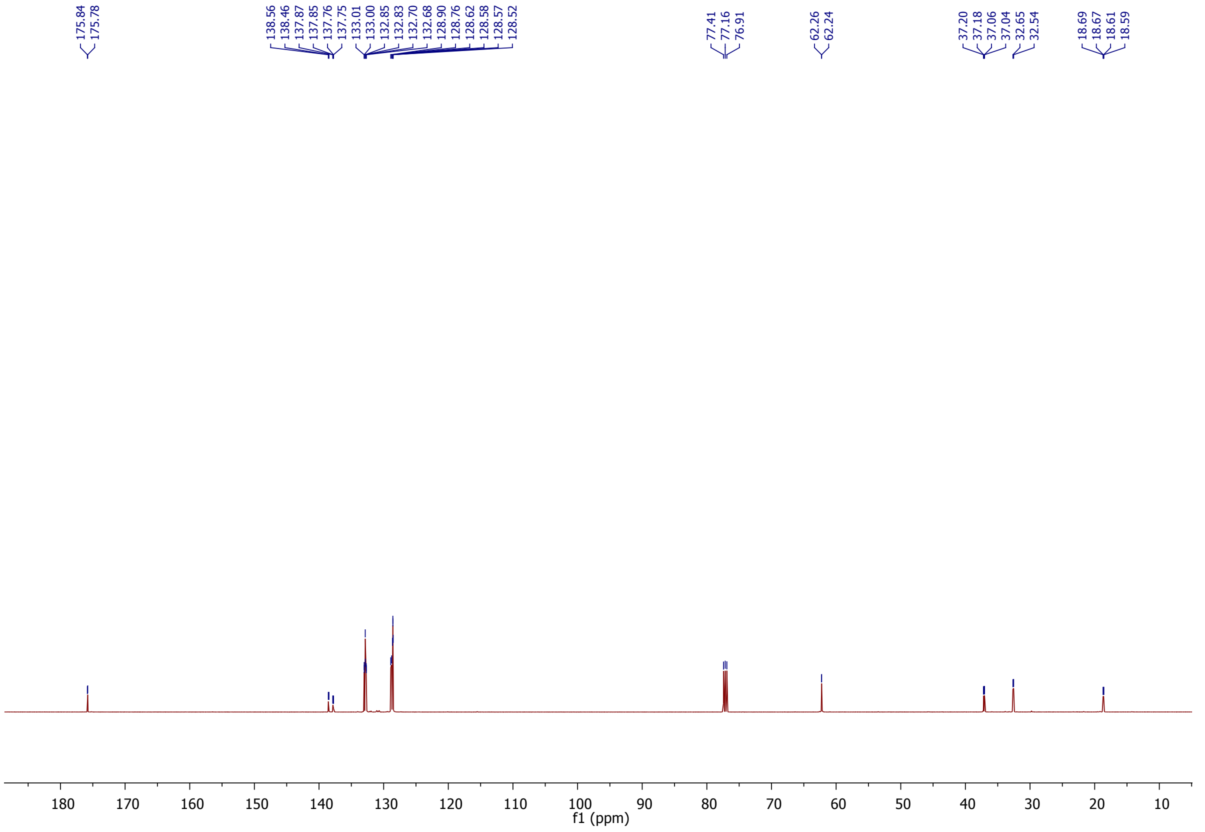

^{31}P NMR (202 MHz) $CDCl_3$

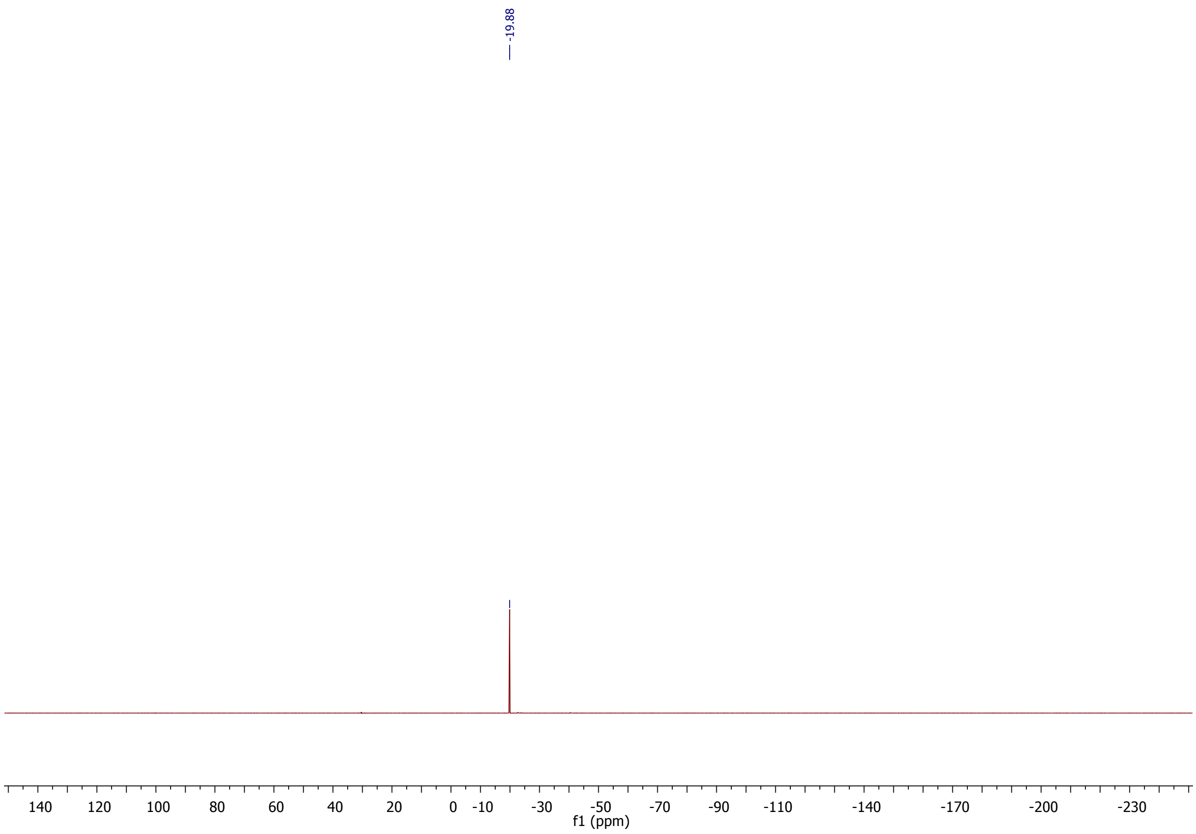

Compound 27

^{1}H NMR (500 MHz) $CDCl_3$

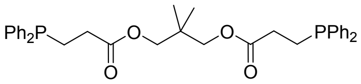

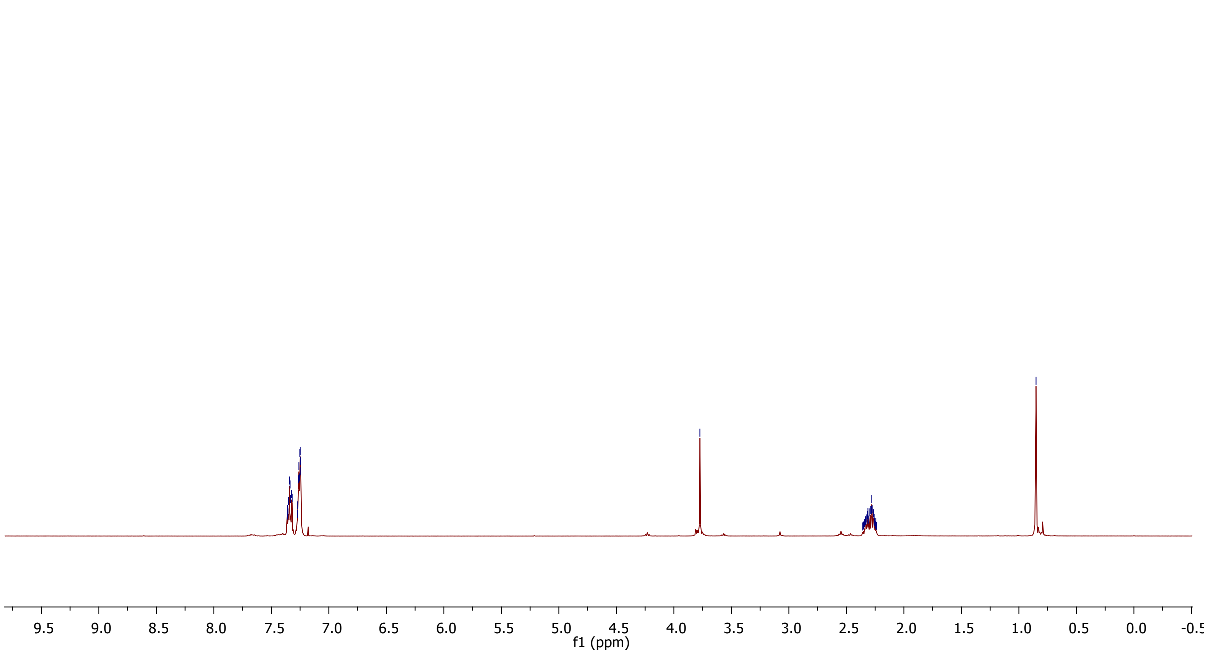

^{13}C NMR (125 MHz) $CDCl_3$

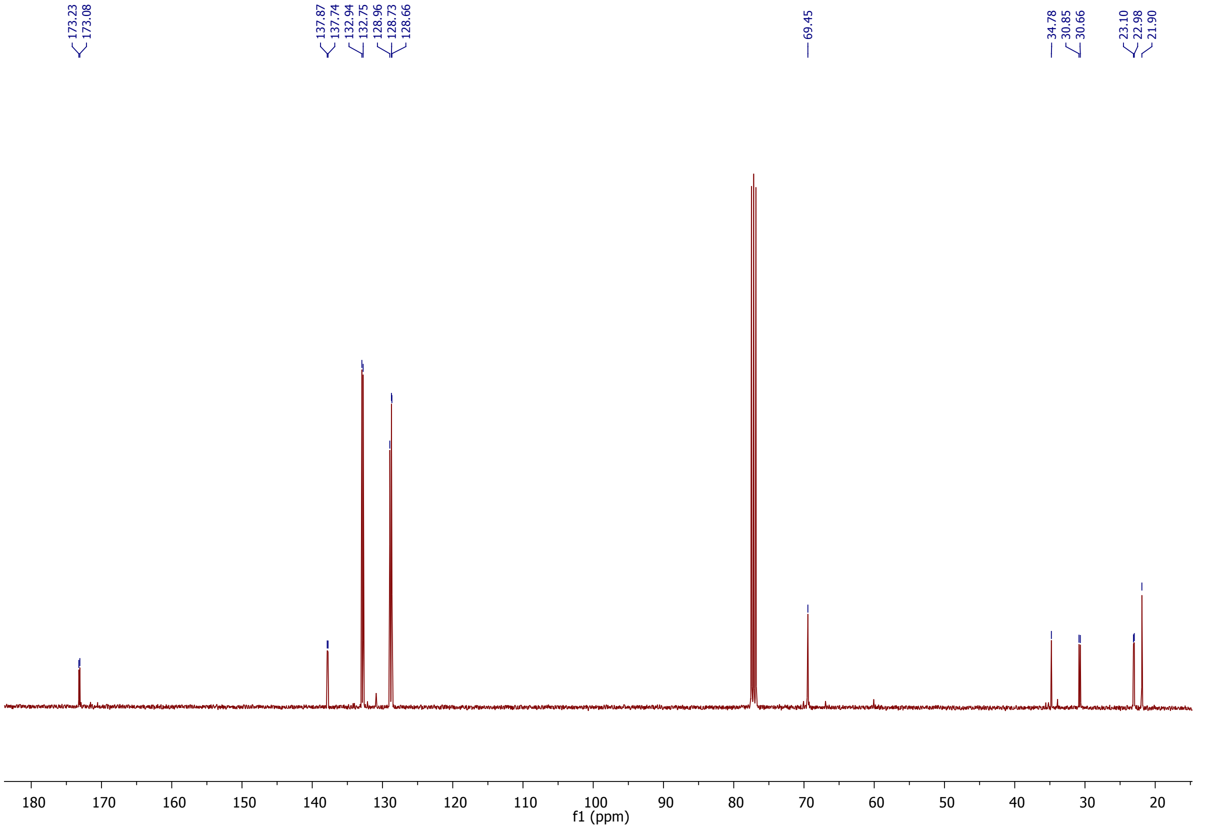

^{31}P NMR (202 MHz) $CDCl_3$

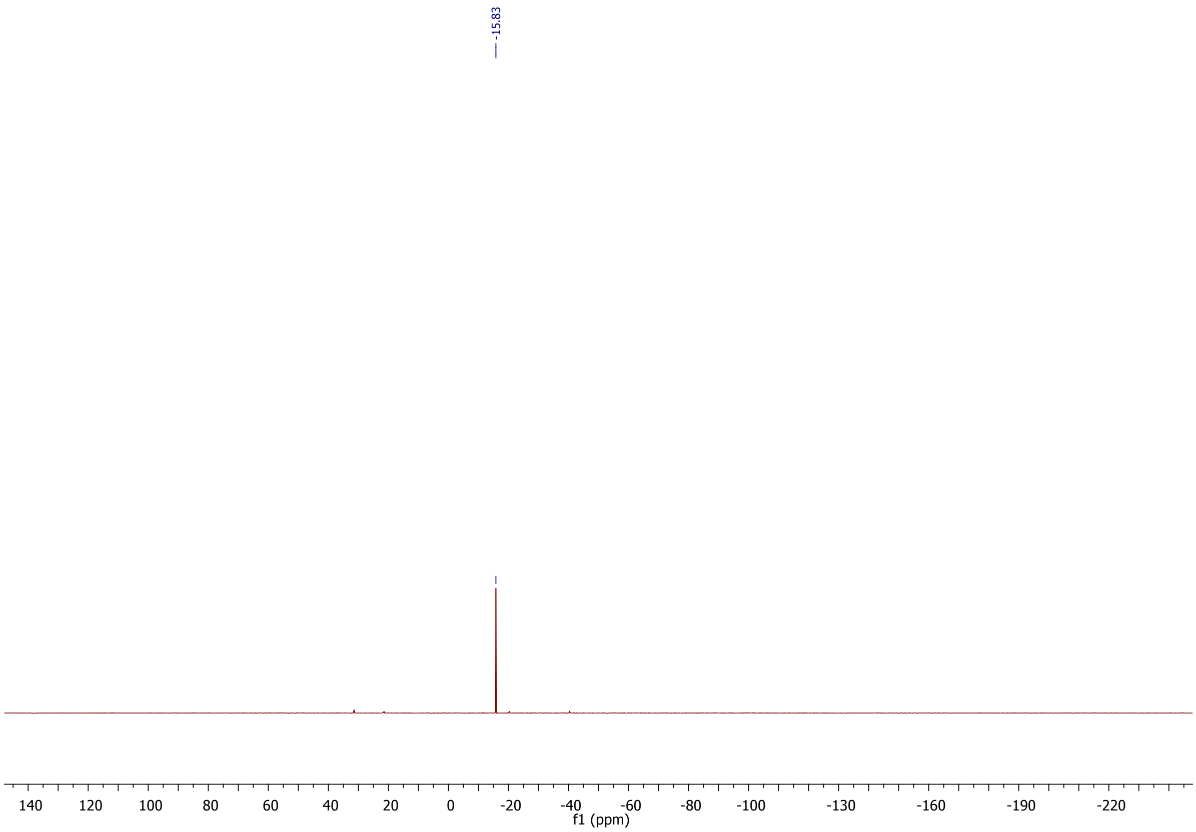

-15.83
140
120
100
80
60
40
20
0
-20
-40
-60
-80
-100
-130
-160
-190
-220
f1 (ppm)

Compound 28

^{1}H NMR (500 MHz) $CDCl_3$

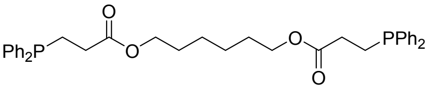

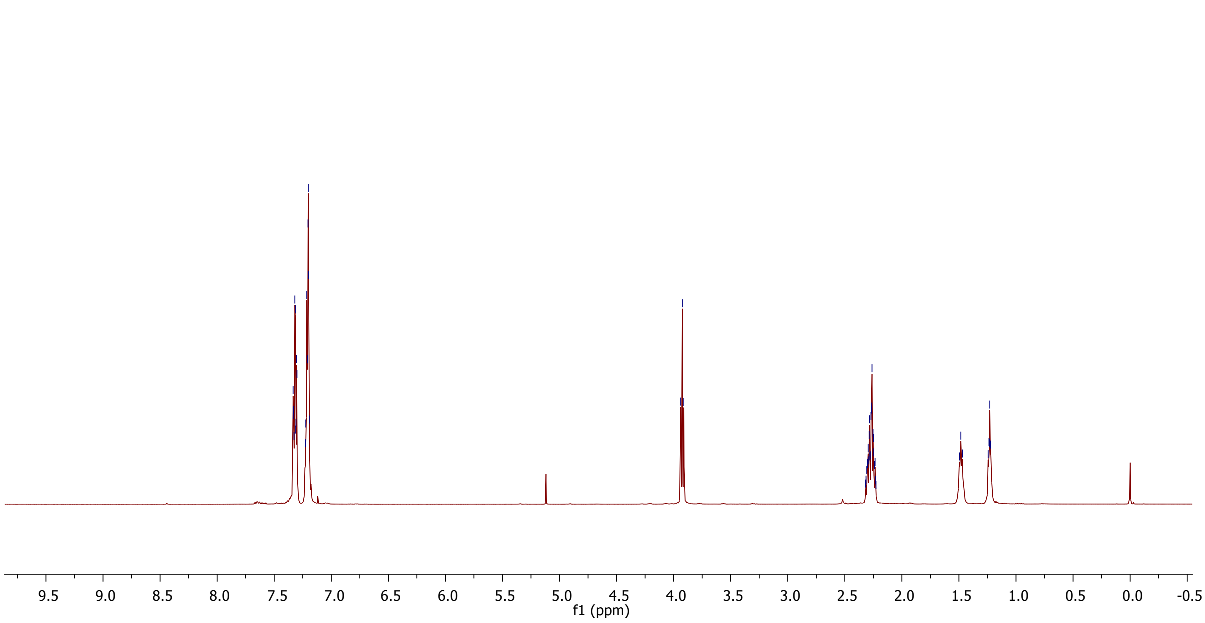

^{13}C NMR (125 MHz) $CDCl_3$

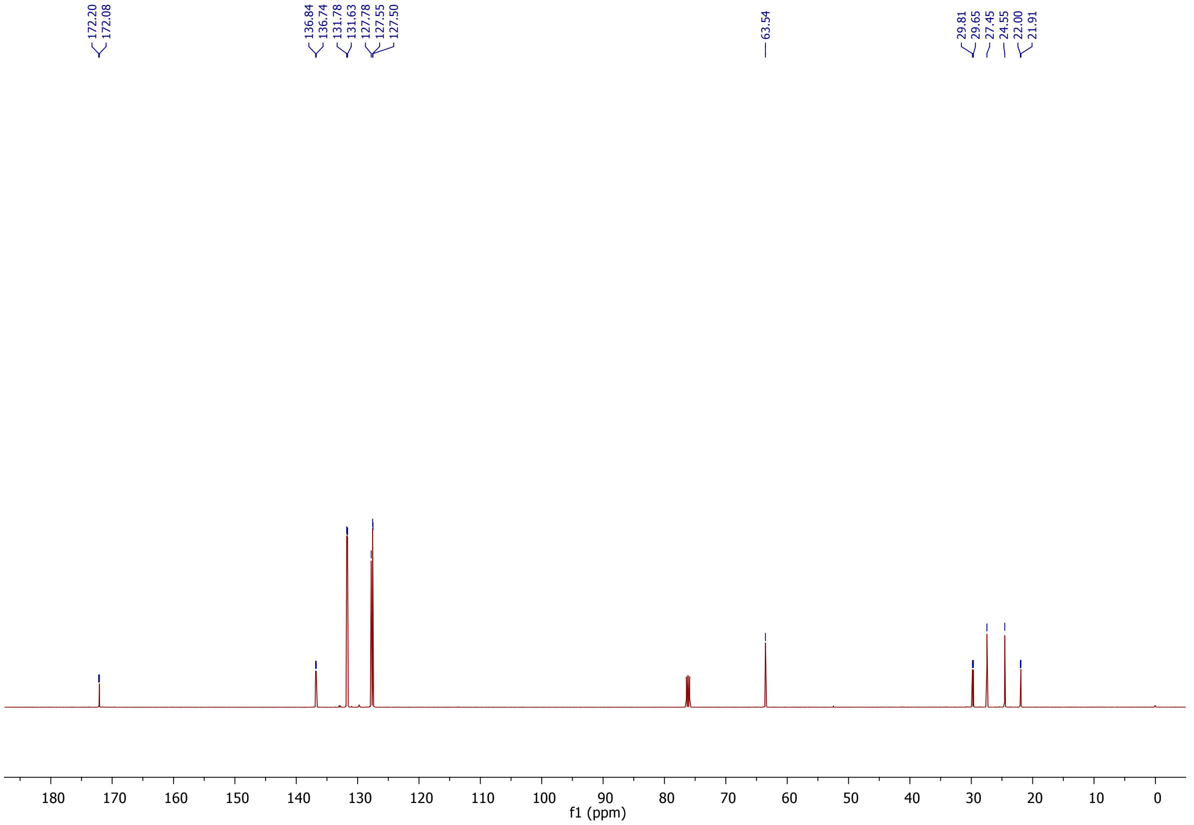

^{31}P NMR (202 MHz) $CDCl_3$

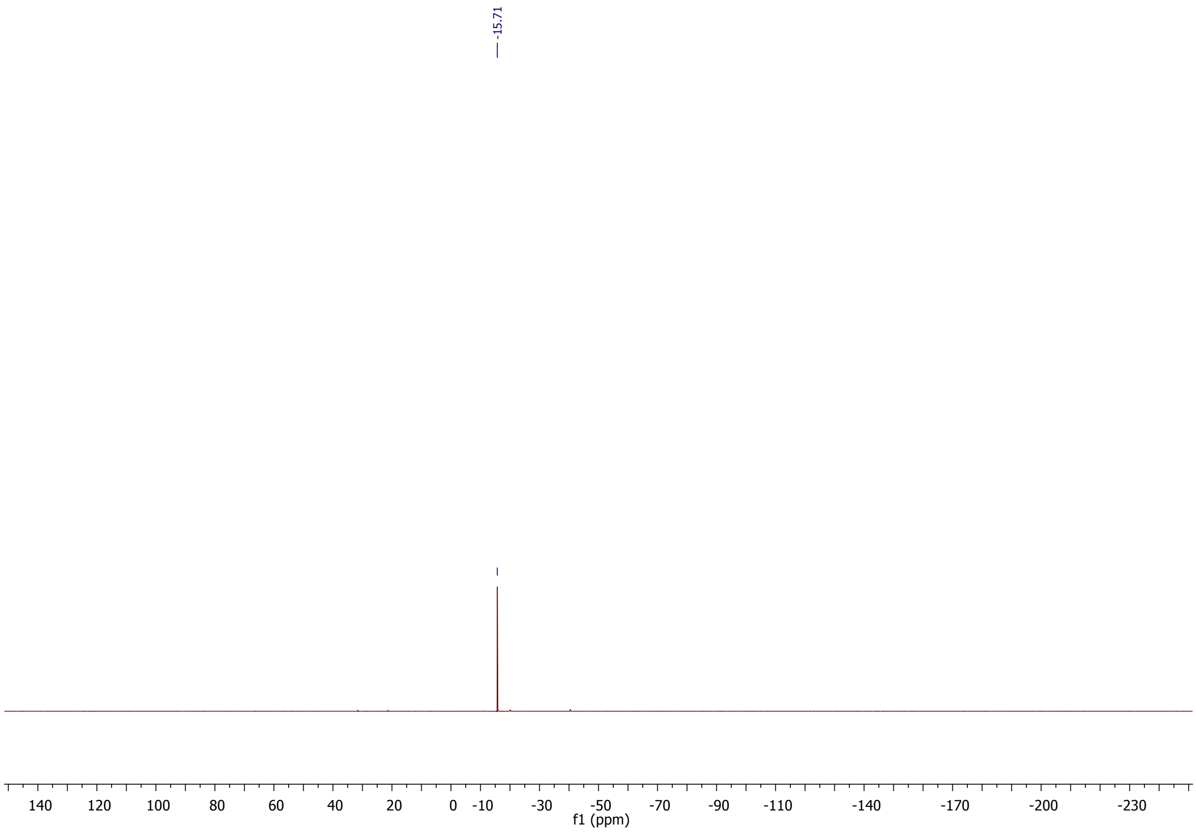

-15.71
140
120
100
80
60
40
20
0
-10
-30
-50
-70
-90
-110
-140
-170
-200
-230
f1 (ppm)

Compound 29

^{1}H NMR (400 MHz) $CDCl_3$

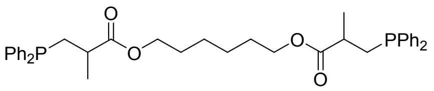

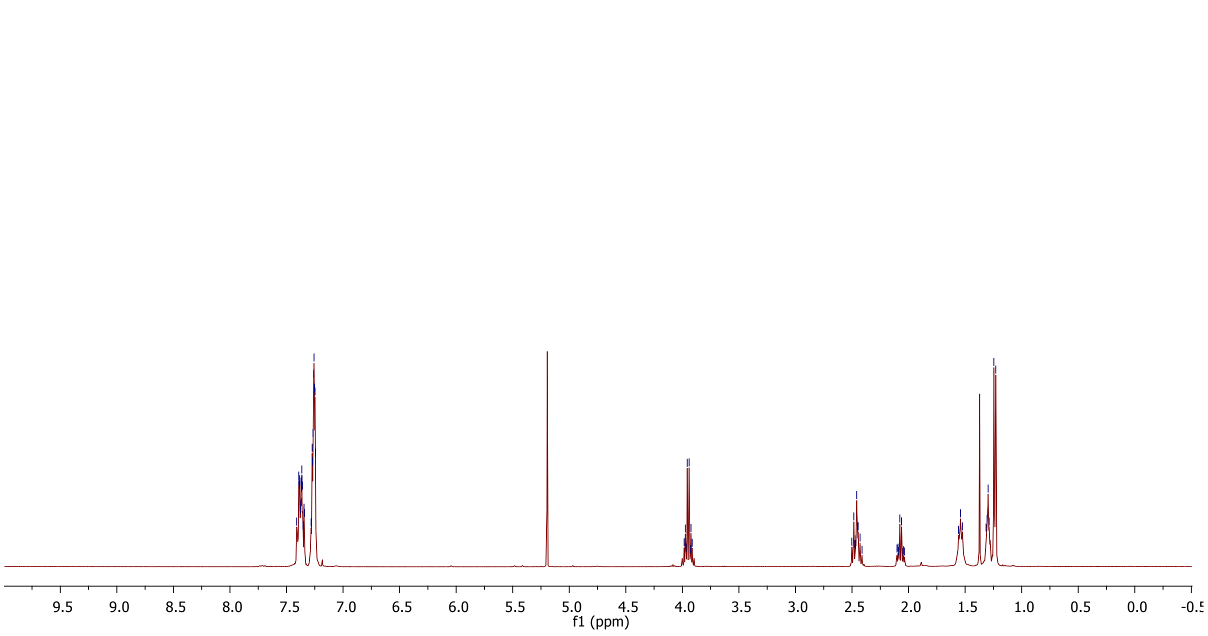

^{13}C NMR (101 MHz) $CDCl_3$

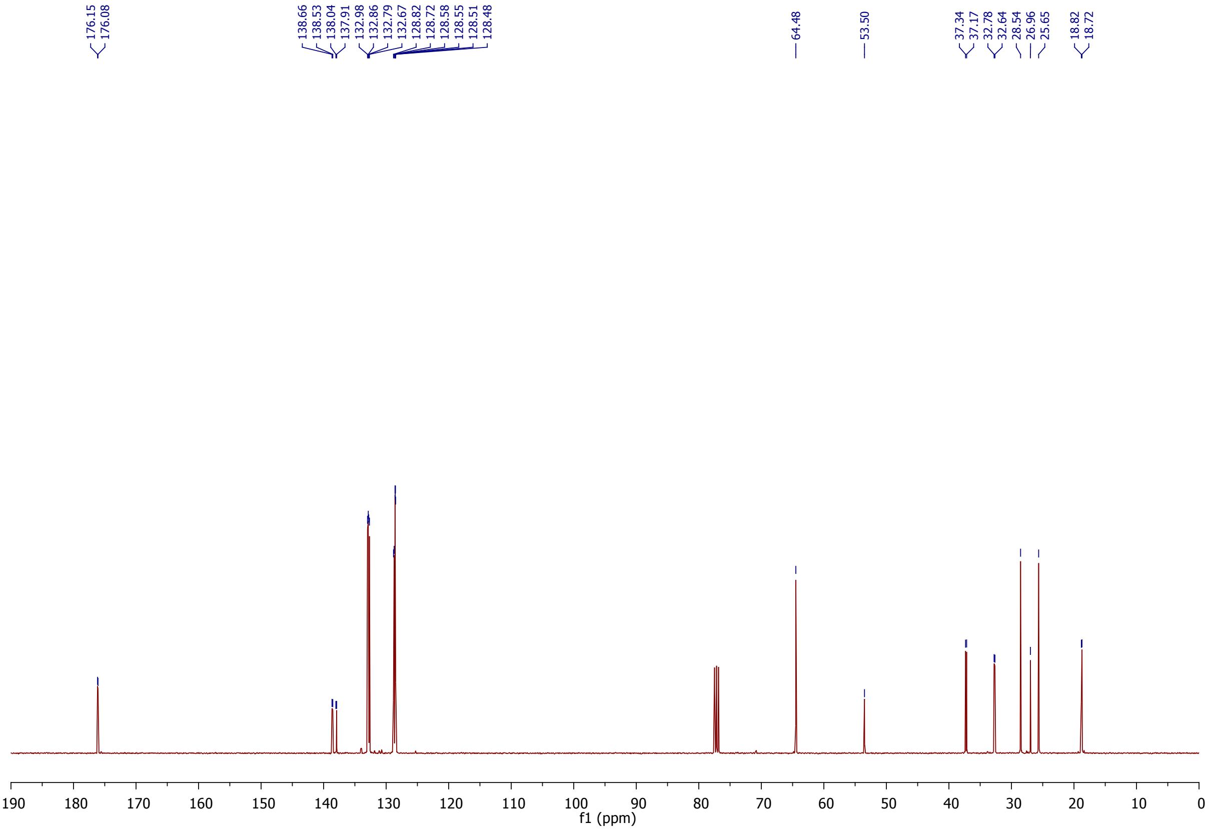

^{31}P NMR (162 MHz) $CDCl_3$

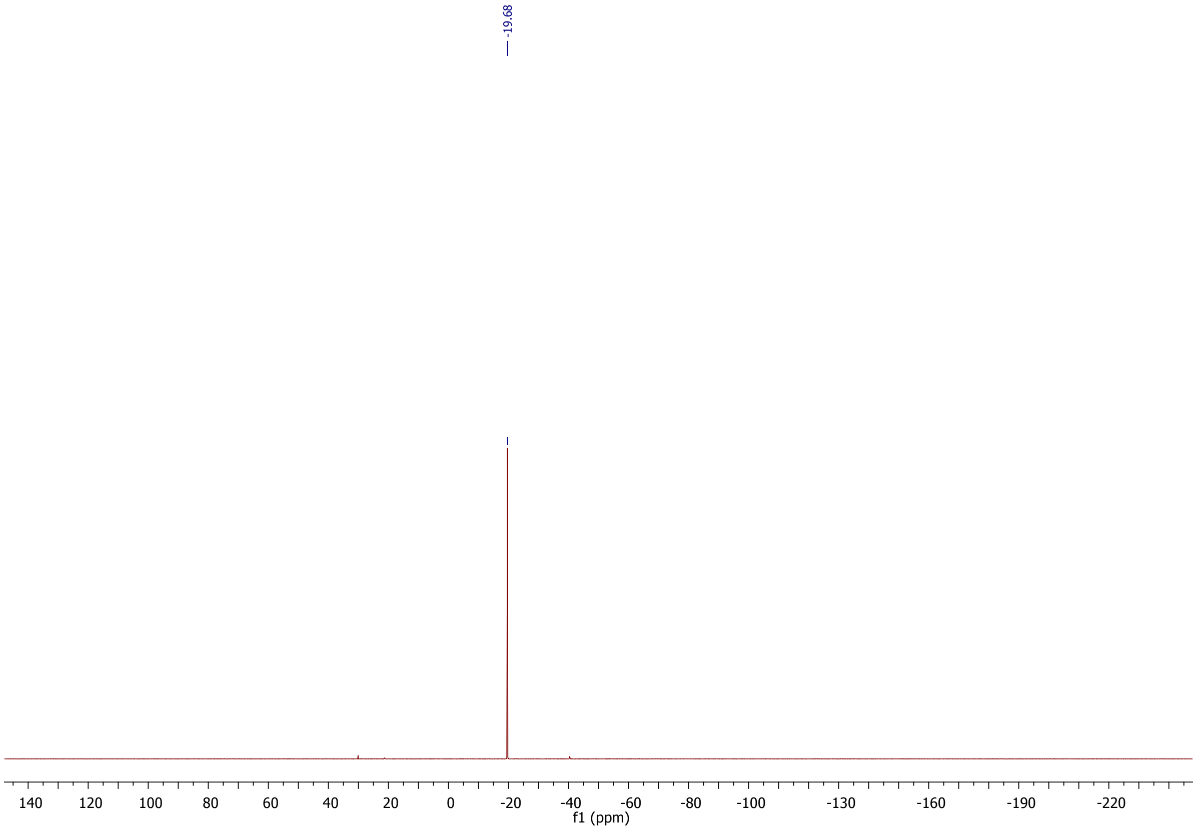

Compound 30

^{1}H NMR (500 MHz) $CDCl_3$

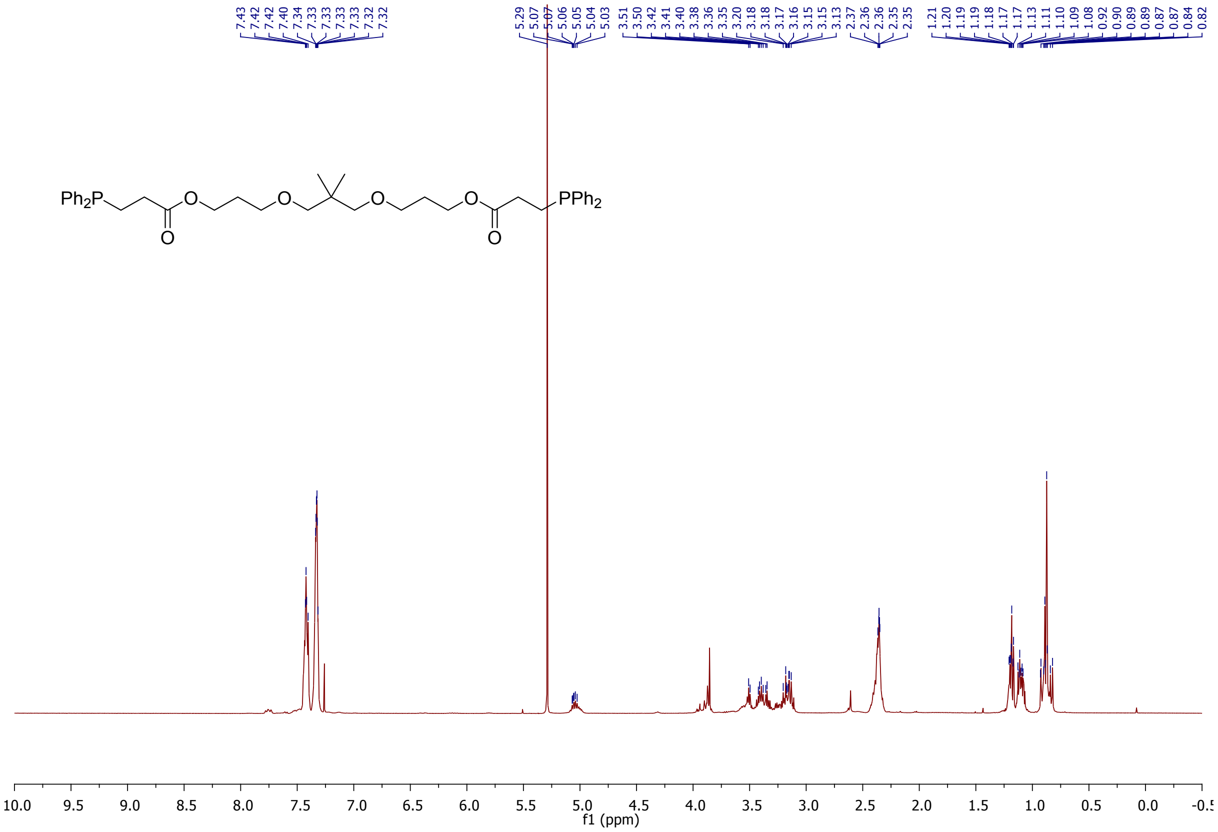

^{13}C NMR (125 MHz) $CDCl_3$

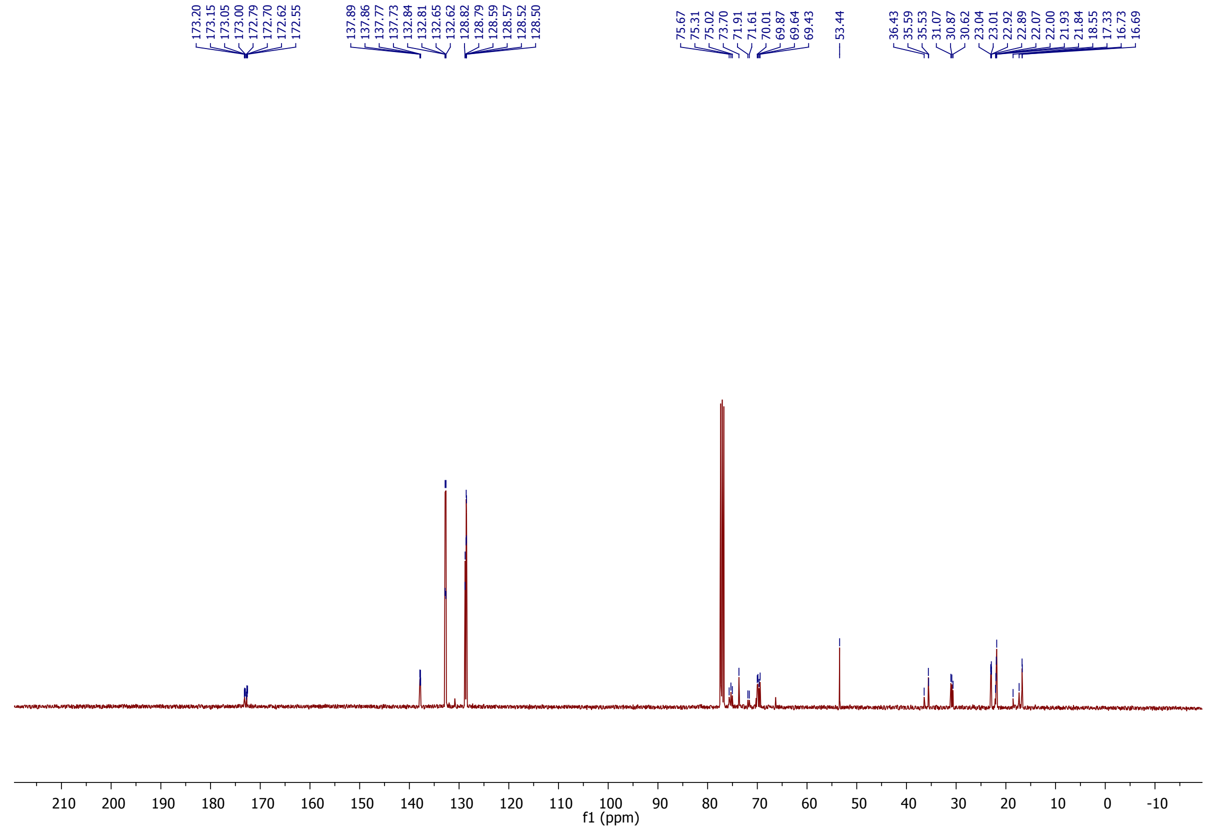

^{31}P NMR (202 MHz) $CDCl_3$

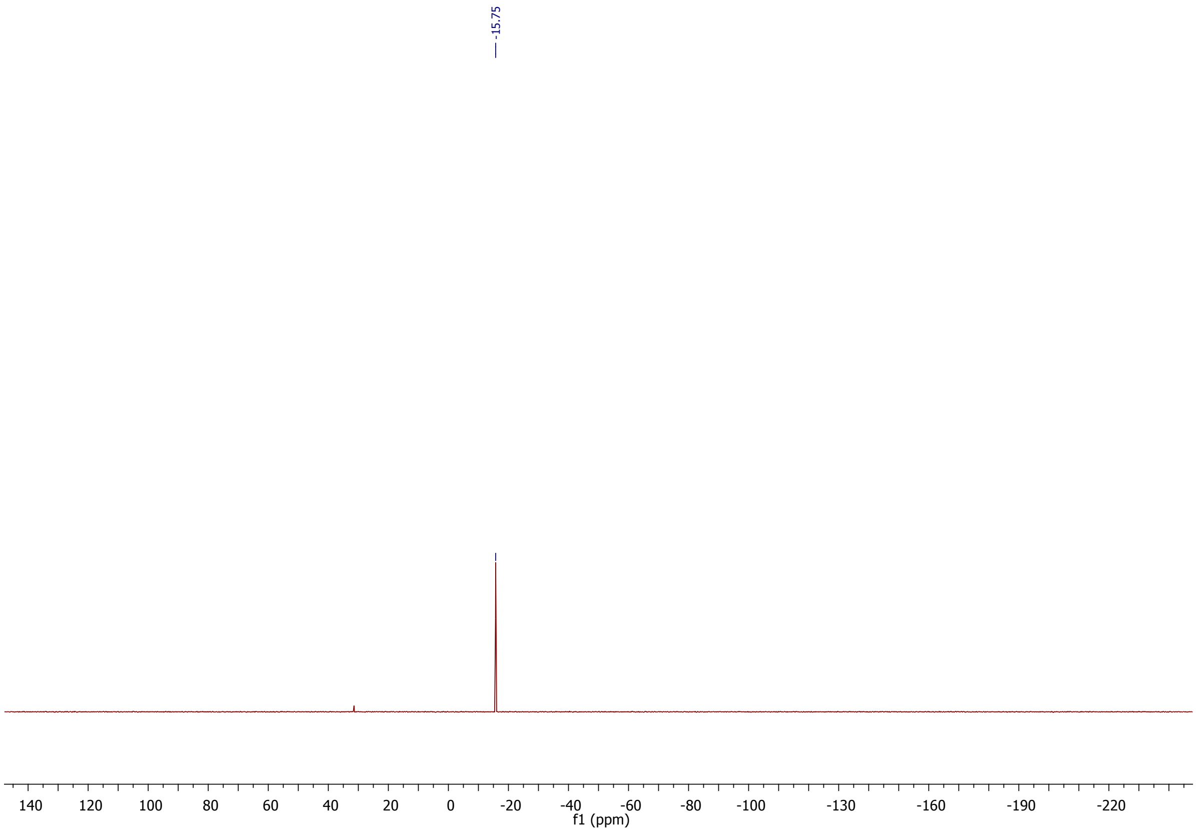

-15.75
140
120
100
80
60
40
20
0
-20
-40
-60
-80
-100
-130
-160
-190
-220
f1 (ppm)

Compound 31

^{1}H NMR (400 MHz) $CDCl_3$

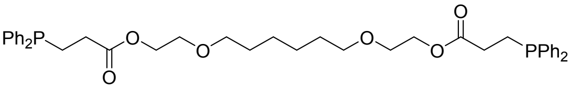

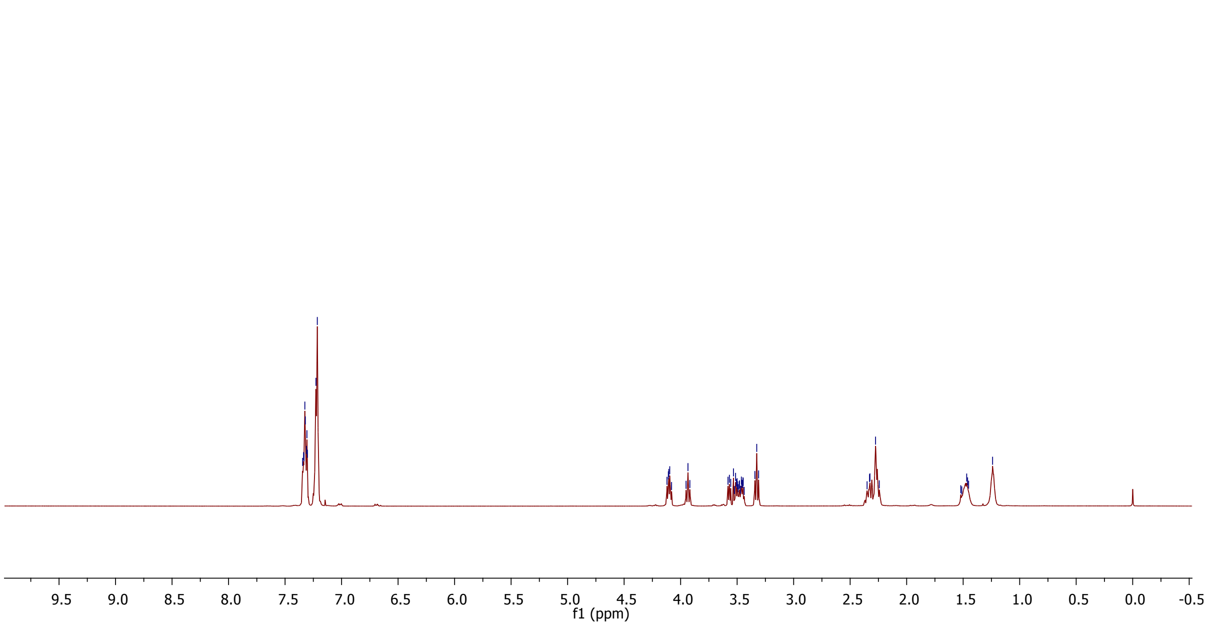

^{13}C NMR (101 MHz) $CDCl_3$

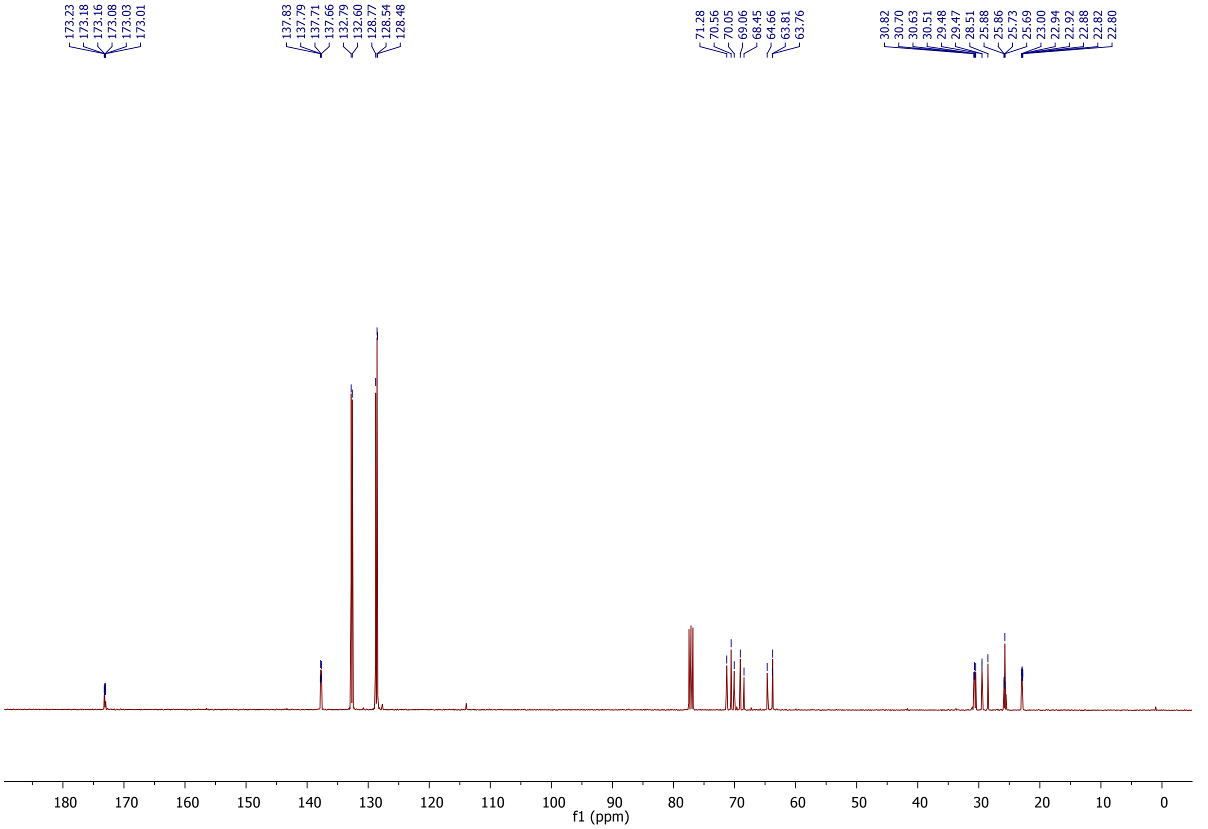

^{31}P NMR (162 MHz) $CDCl_3$

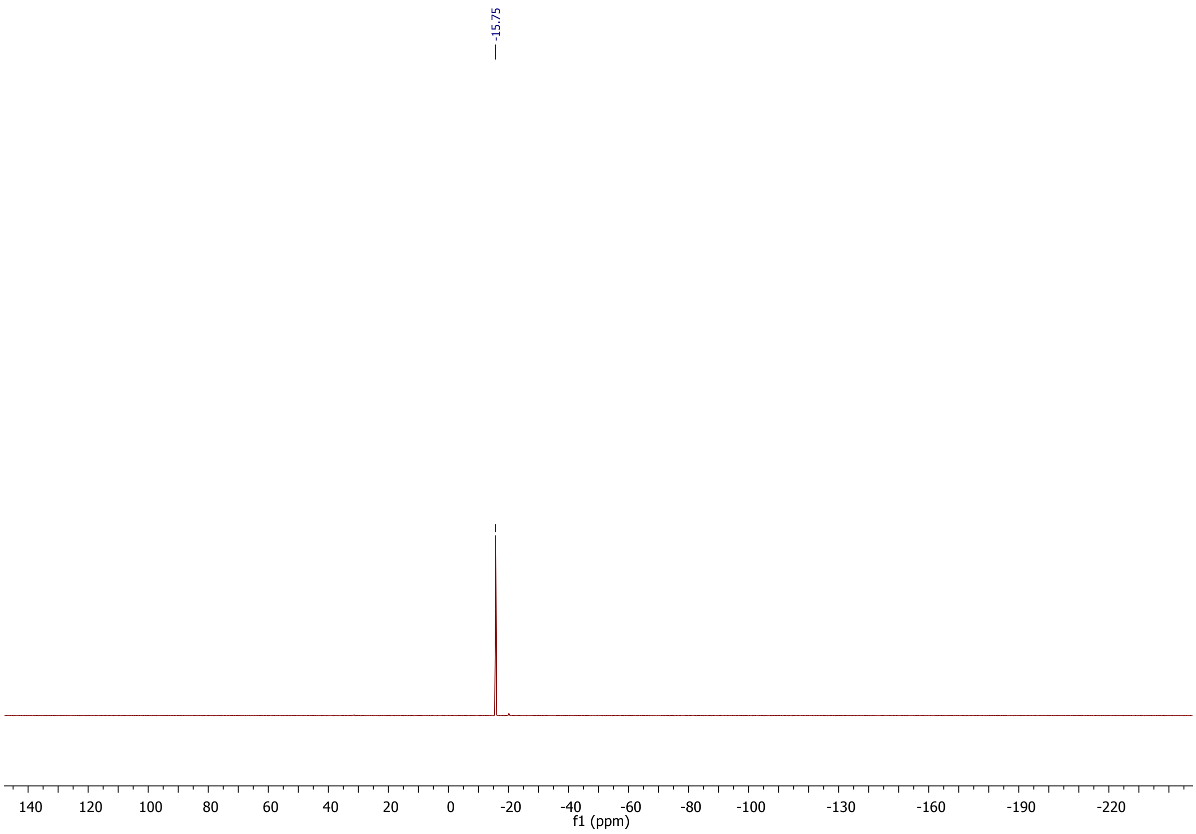

Compound 32

^{1}H NMR (400 MHz) $CDCl_3$

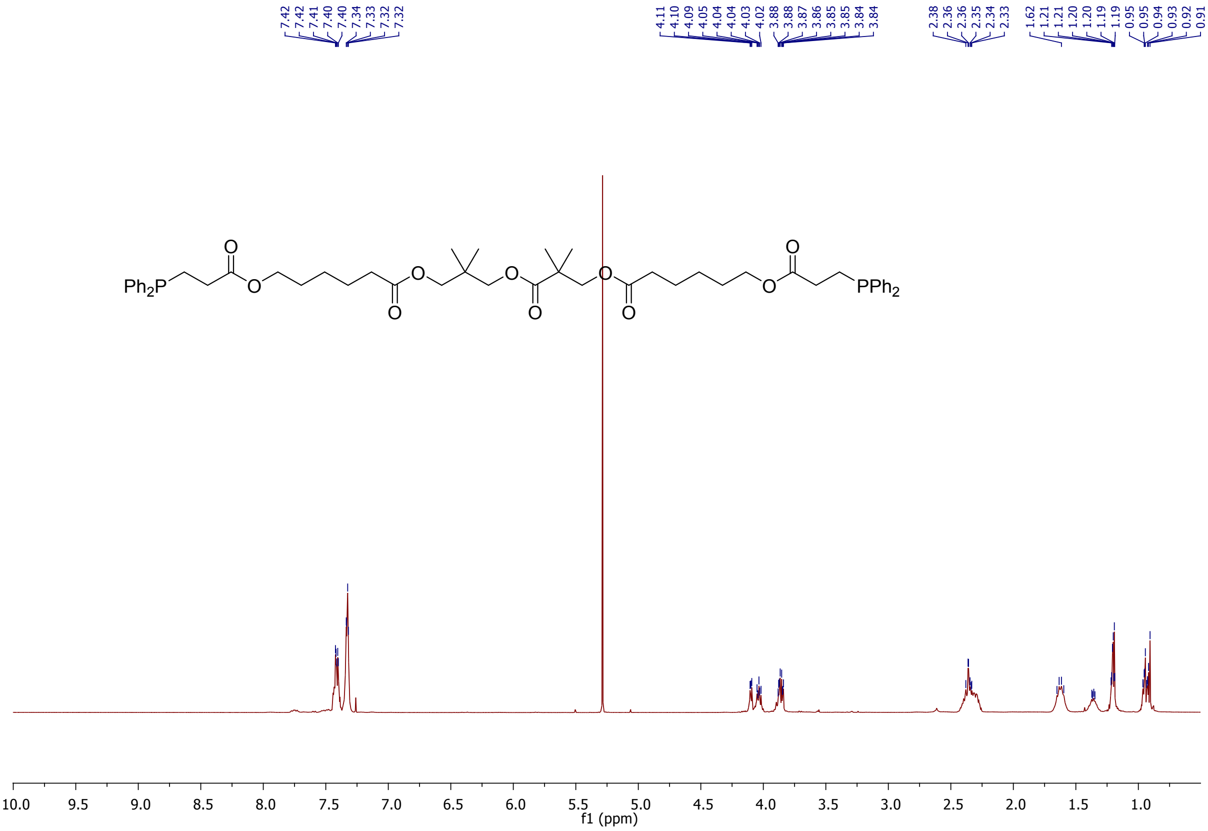

^{13}C NMR (101 MHz) $CDCl_3$

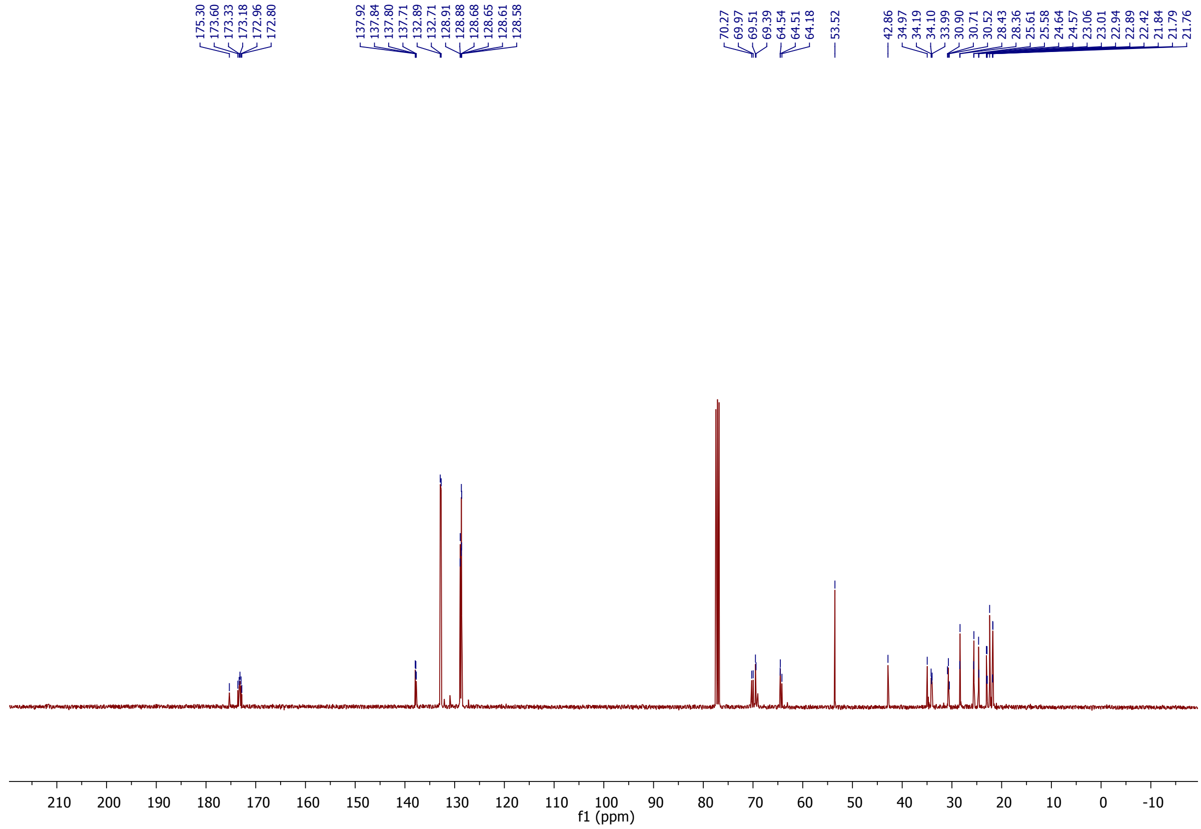

^{31}P NMR (162 MHz) $CDCl_3$

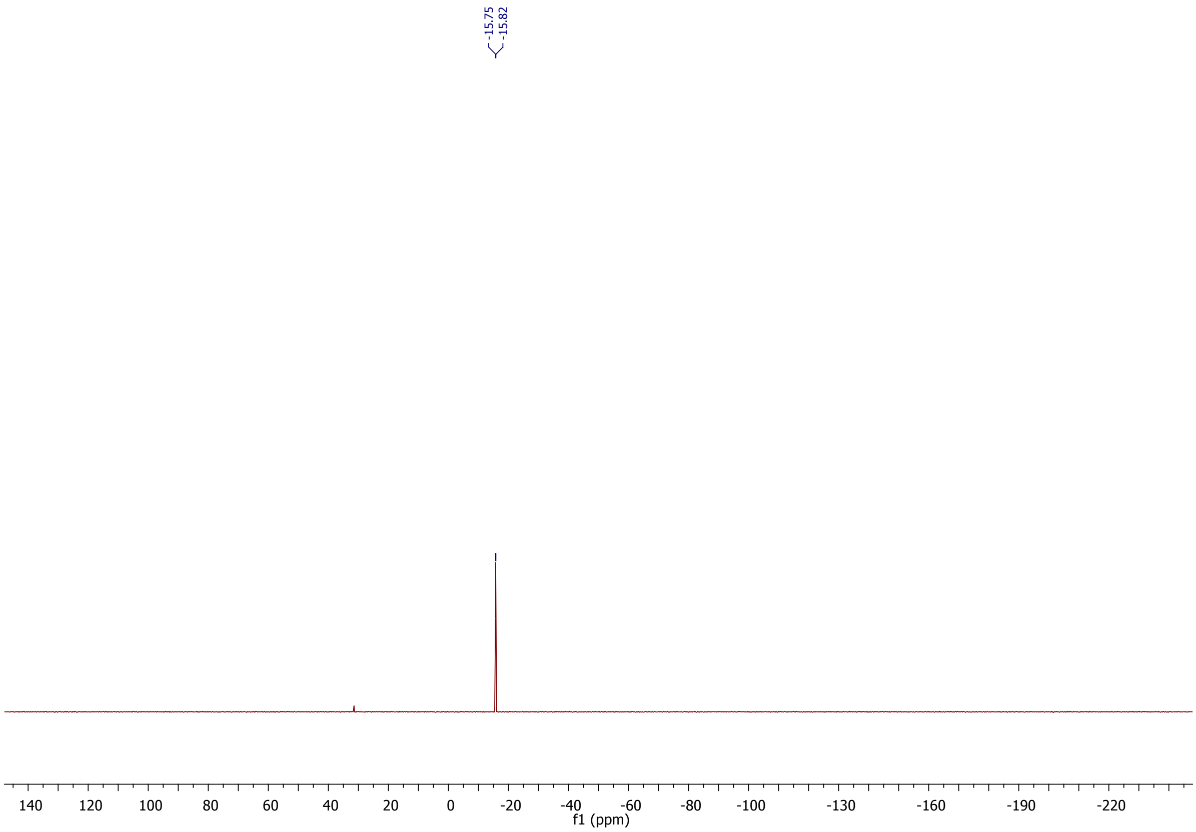

-15.75
-15.82
140 120 100 80 60 40 20 0 -20 -40 -60 -80 -100 -130 -160 -190 -220
f1 (ppm)

4.3 *Tris- and tetra-acrylate derivatives*

Compound 33

^{1}H NMR (400 MHz) $CDCl_3$

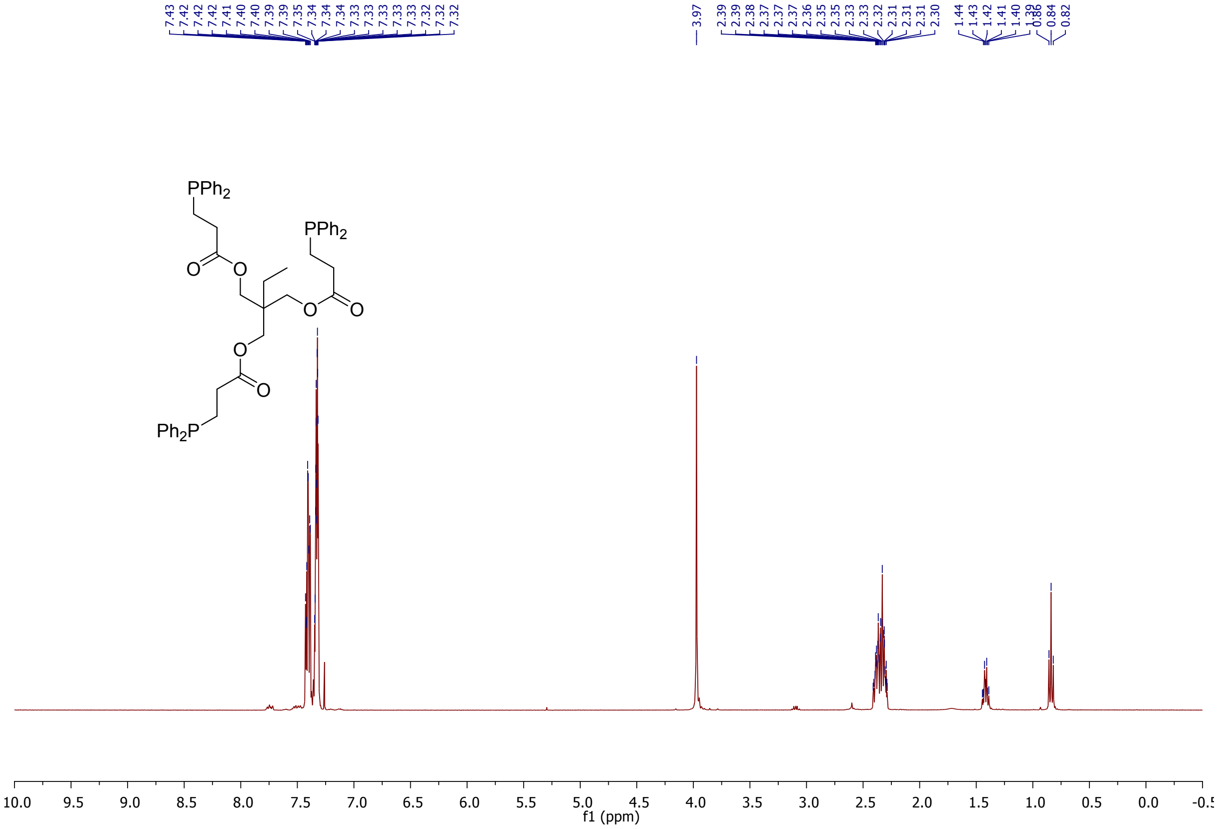

^{13}C NMR (101 MHz) $CDCl_3$

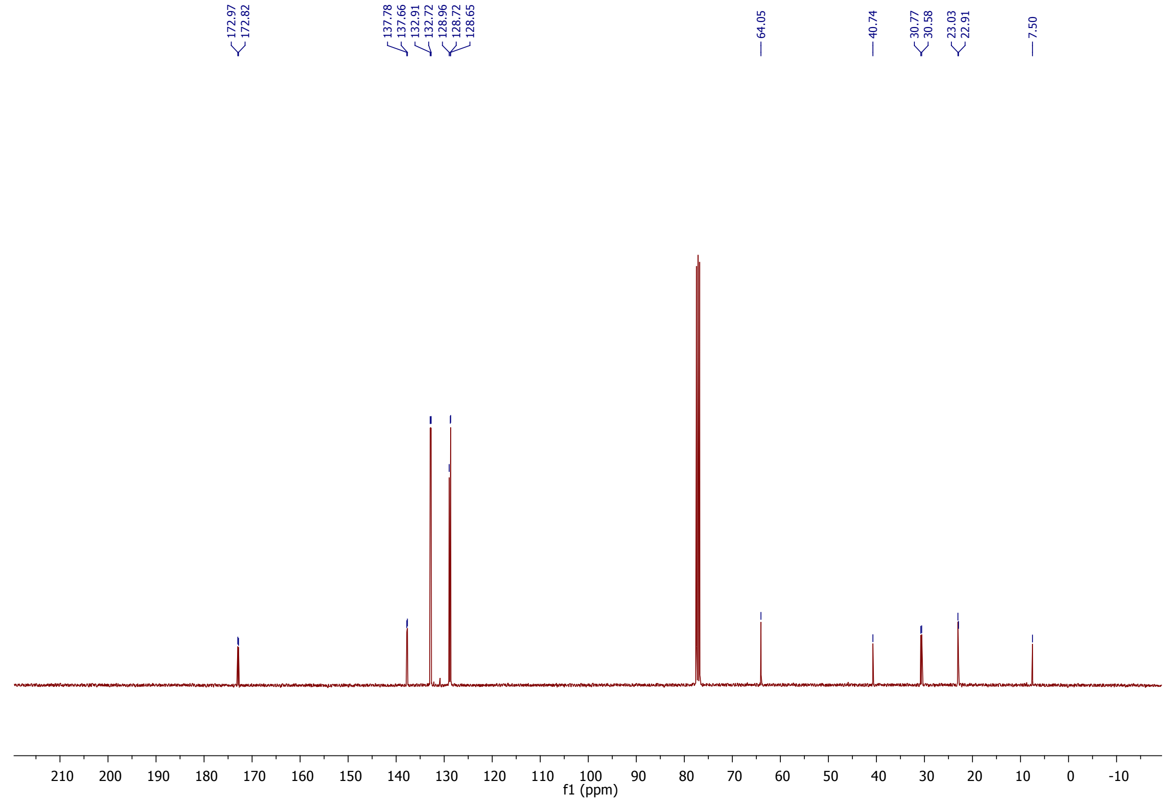

^{31}P NMR (162 MHz) $CDCl_3$

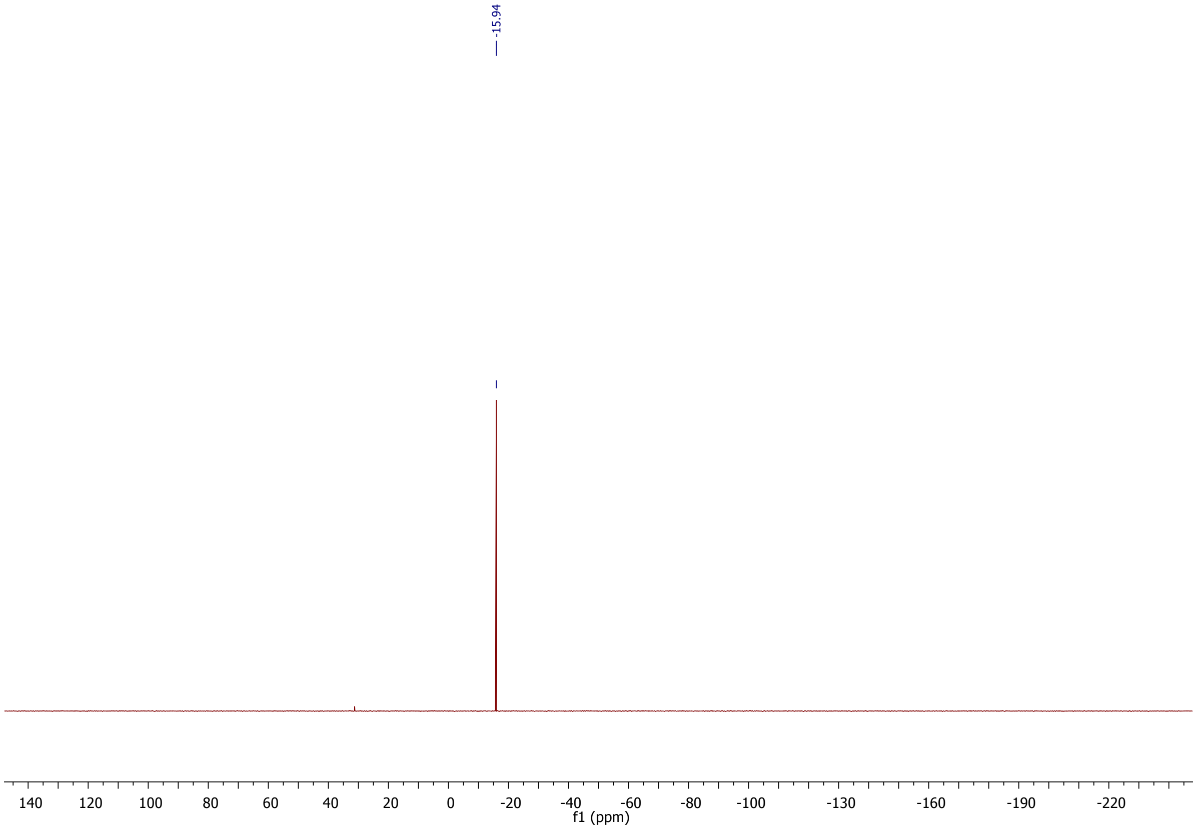

Compound 34

^{1}H NMR (400 MHz) $CDCl_3$

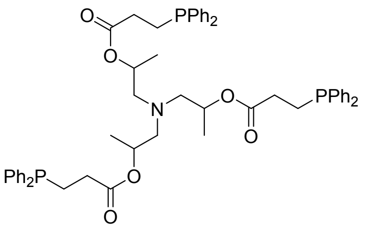

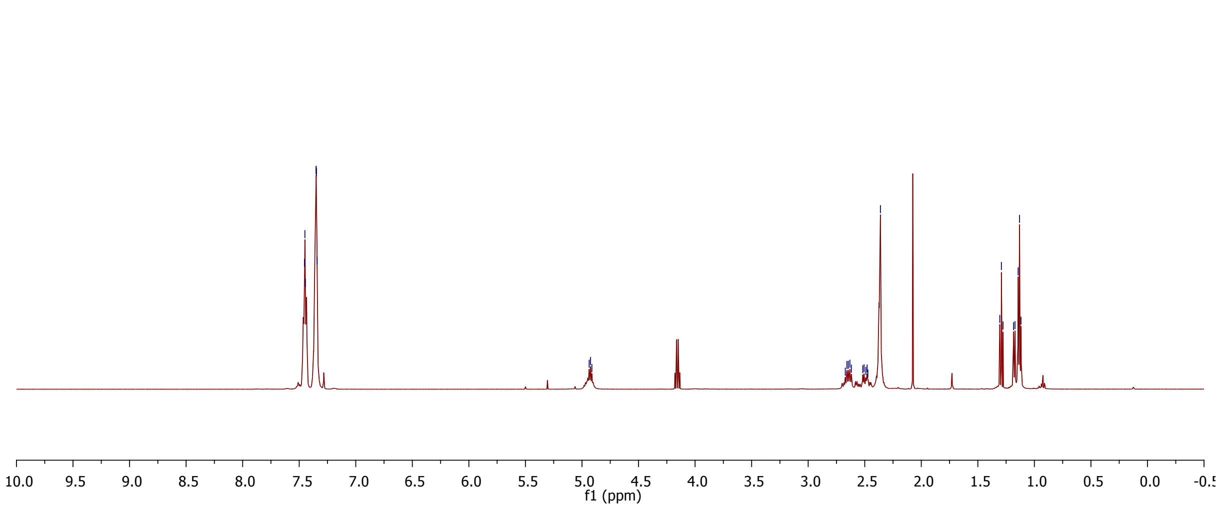

^{13}C NMR (101 MHz) $CDCl_3$

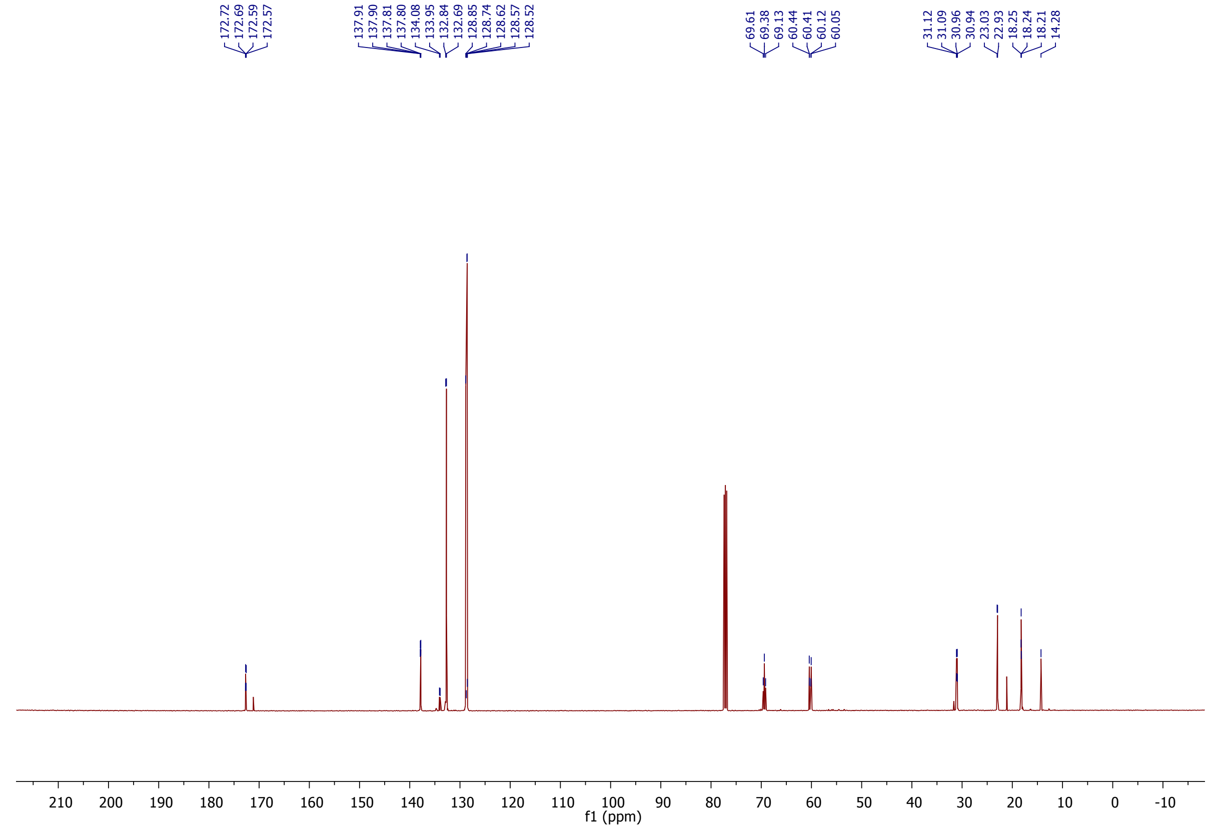

^{31}P NMR (162 MHz) $CDCl_3$

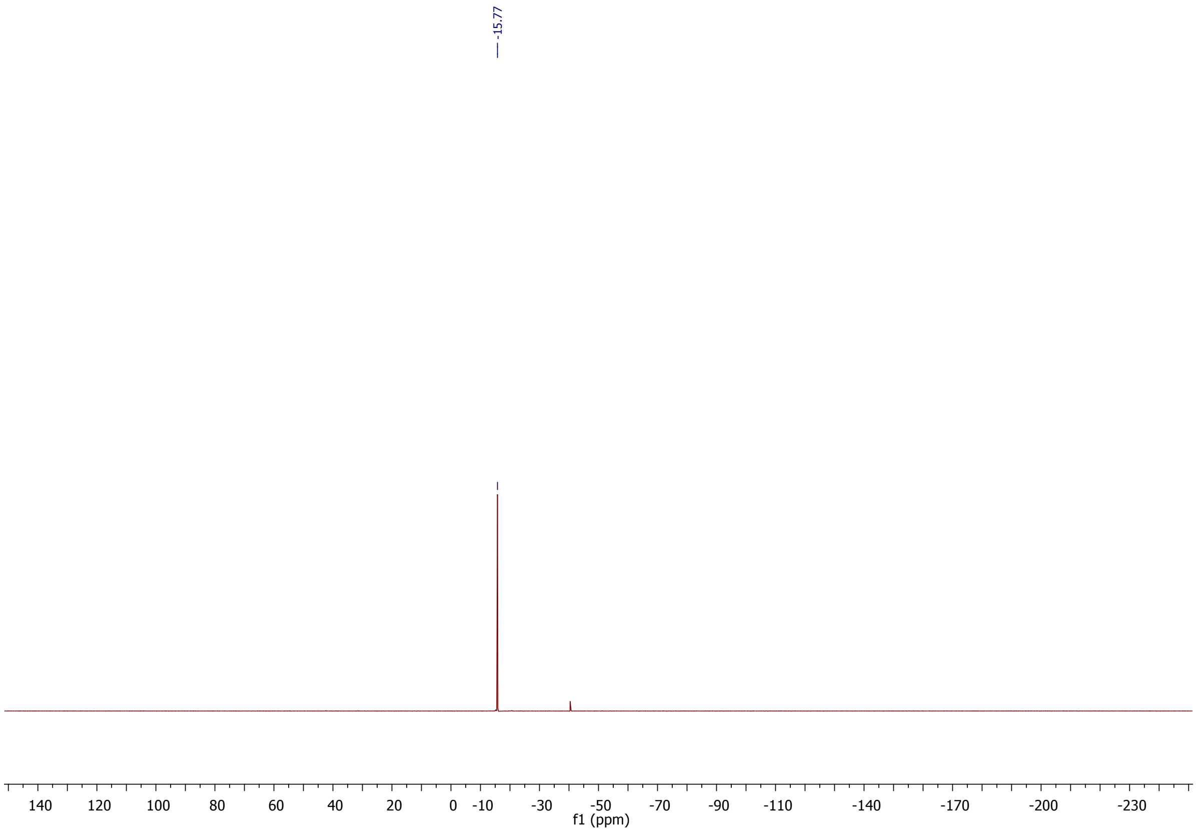

-15.77
140
120
100
80
60
40
20
0
-10
-30
-50
-70
-90
-110
-140
-170
-200
-230
f1 (ppm)

Compound 35

^{1}H NMR (400 MHz) $CDCl_3$

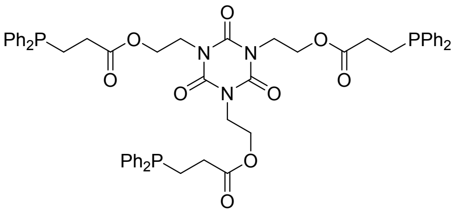

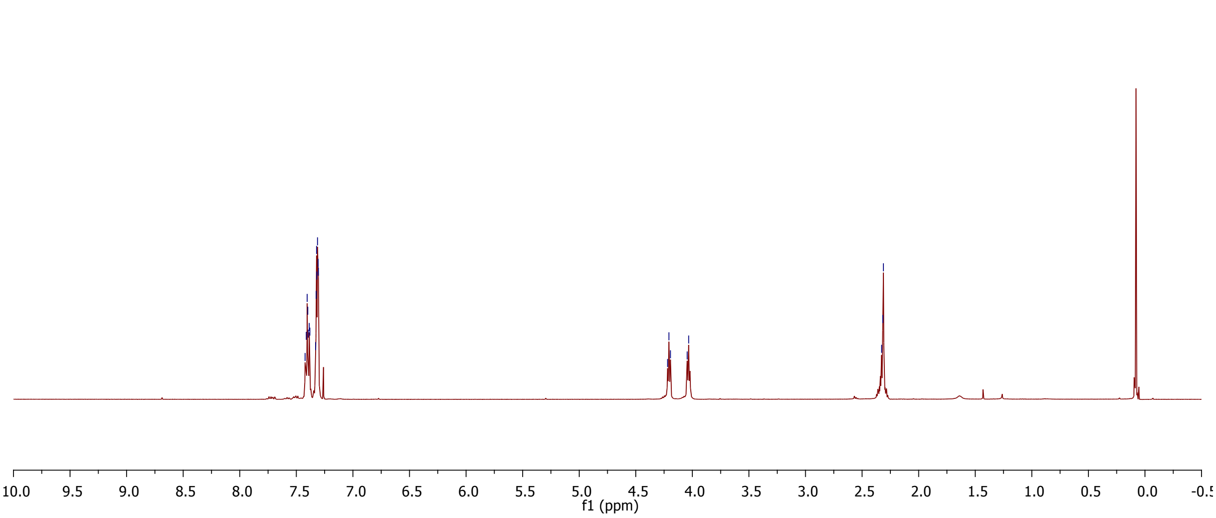

^{13}C NMR (101 MHz) $CDCl_3$

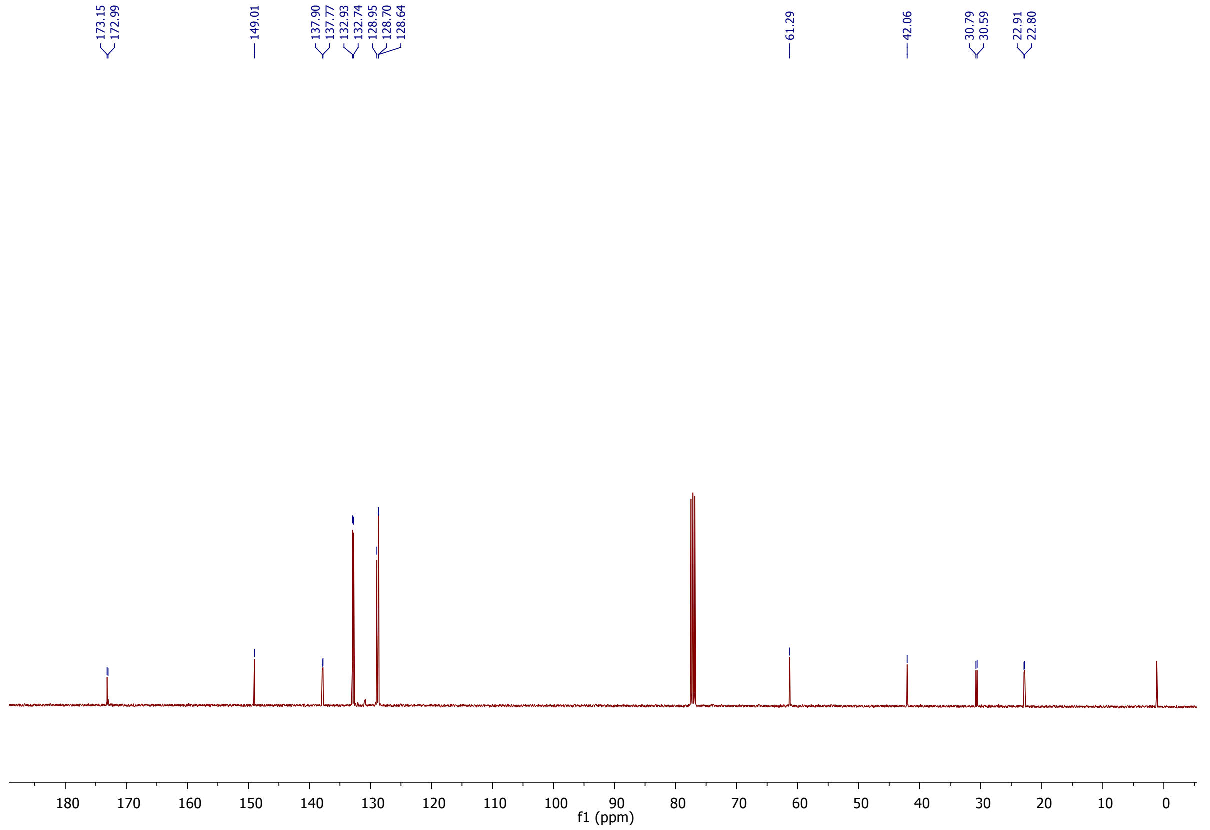

^{31}P NMR (162 MHz) $CDCl_3$

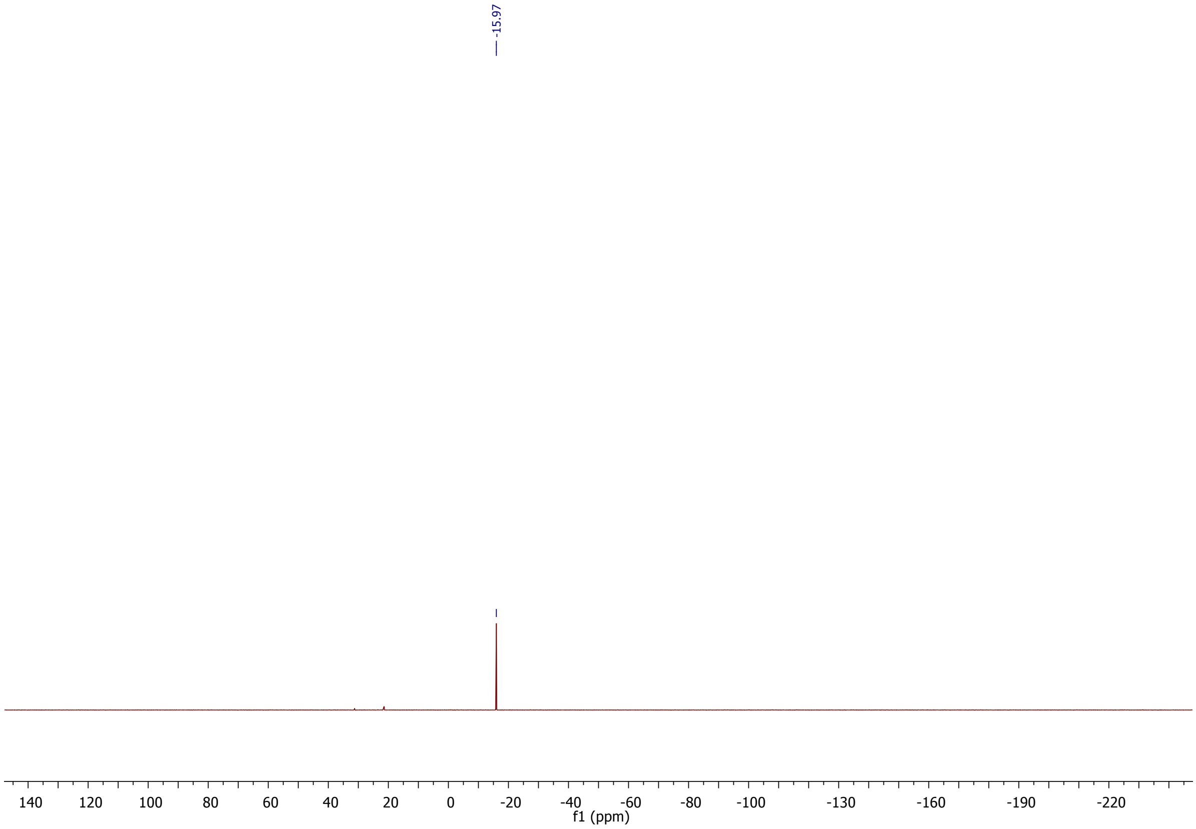

Compound 36

^{1}H NMR (500 MHz) $CDCl_3$

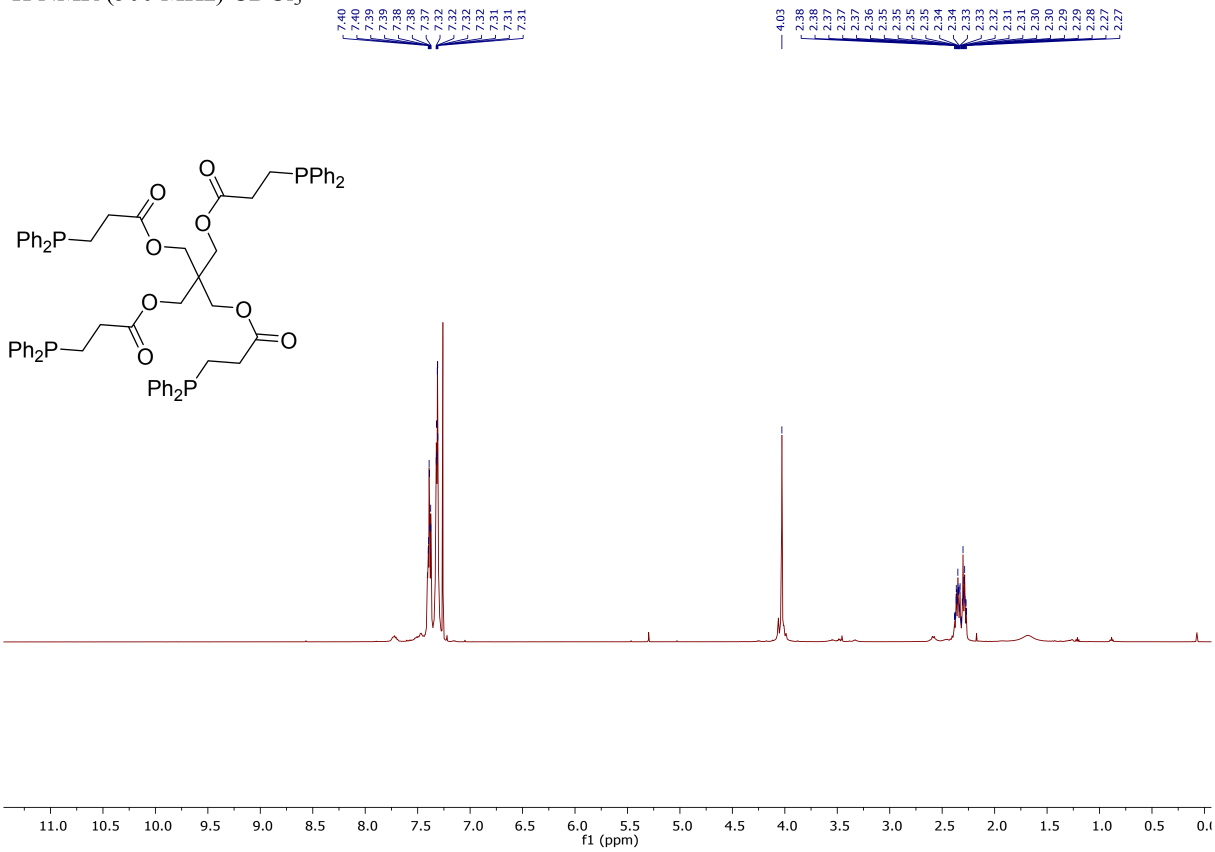

^{13}C NMR (125 MHz) $CDCl_3$

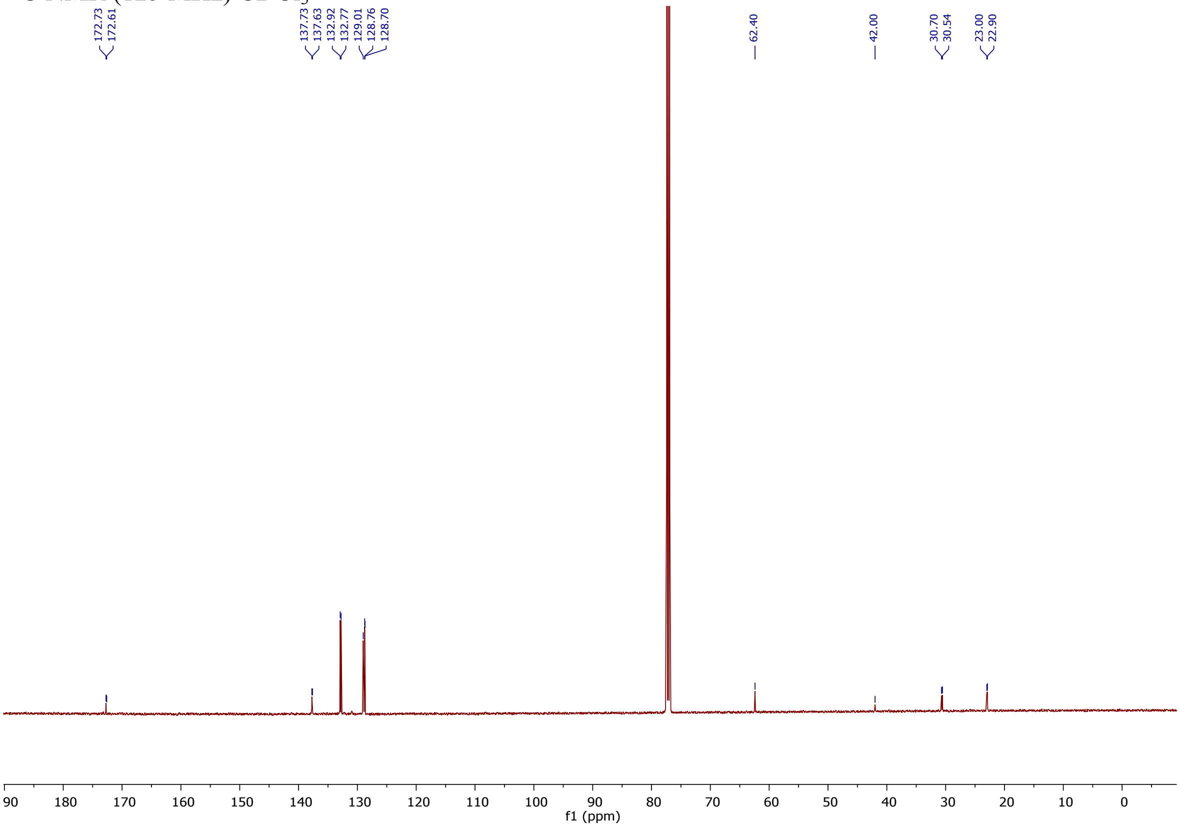

^{31}P NMR (202 MHz) $CDCl_3$

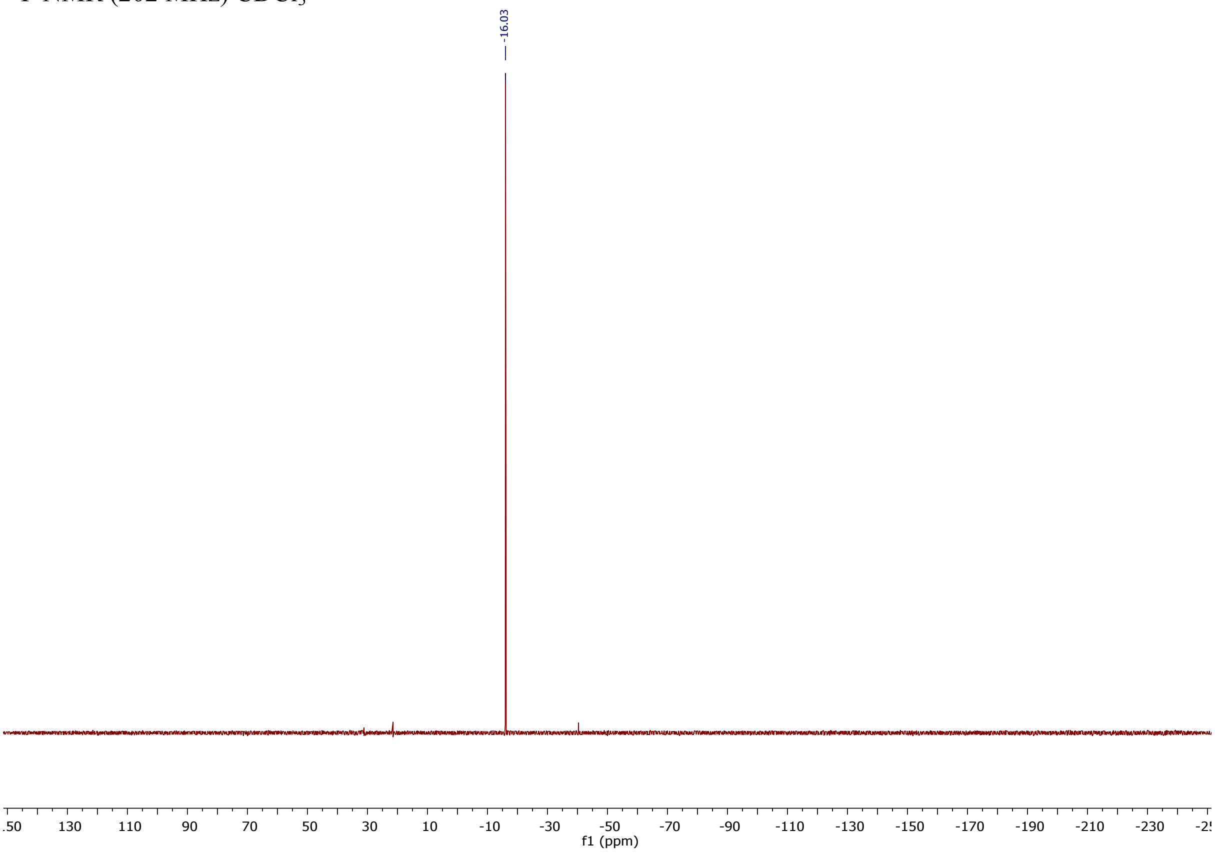

Compound 37

^{1}H NMR (400 MHz) $CDCl_3$

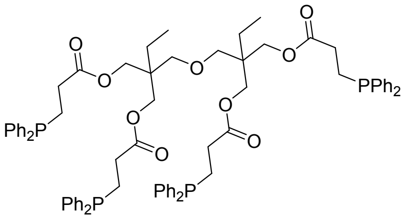

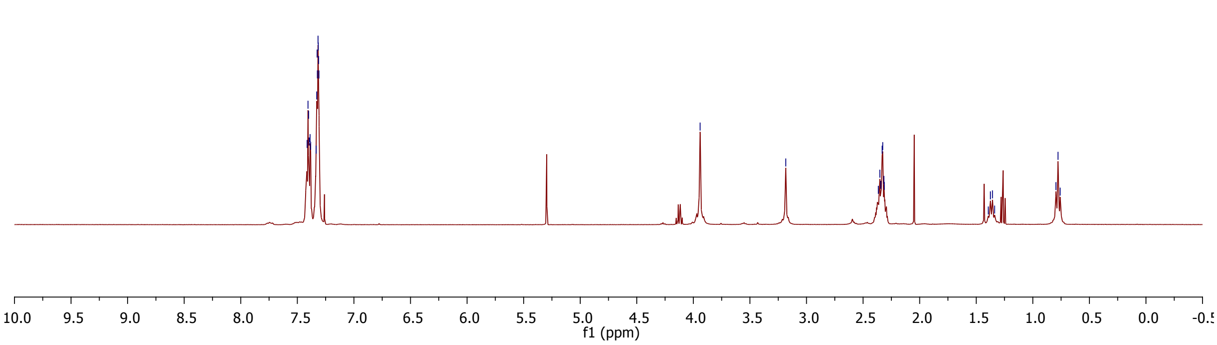

^{13}C NMR (101 MHz) $CDCl_3$

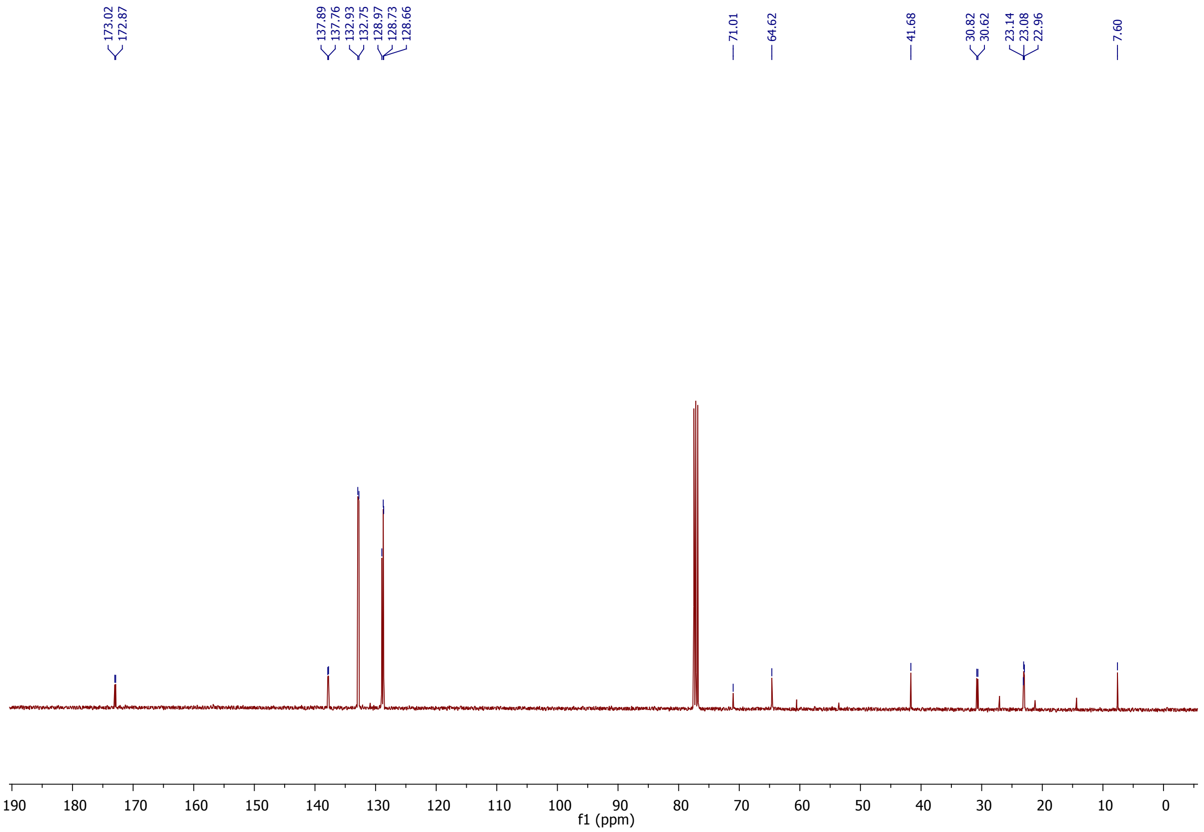

^{31}P NMR (162 MHz) $CDCl_3$

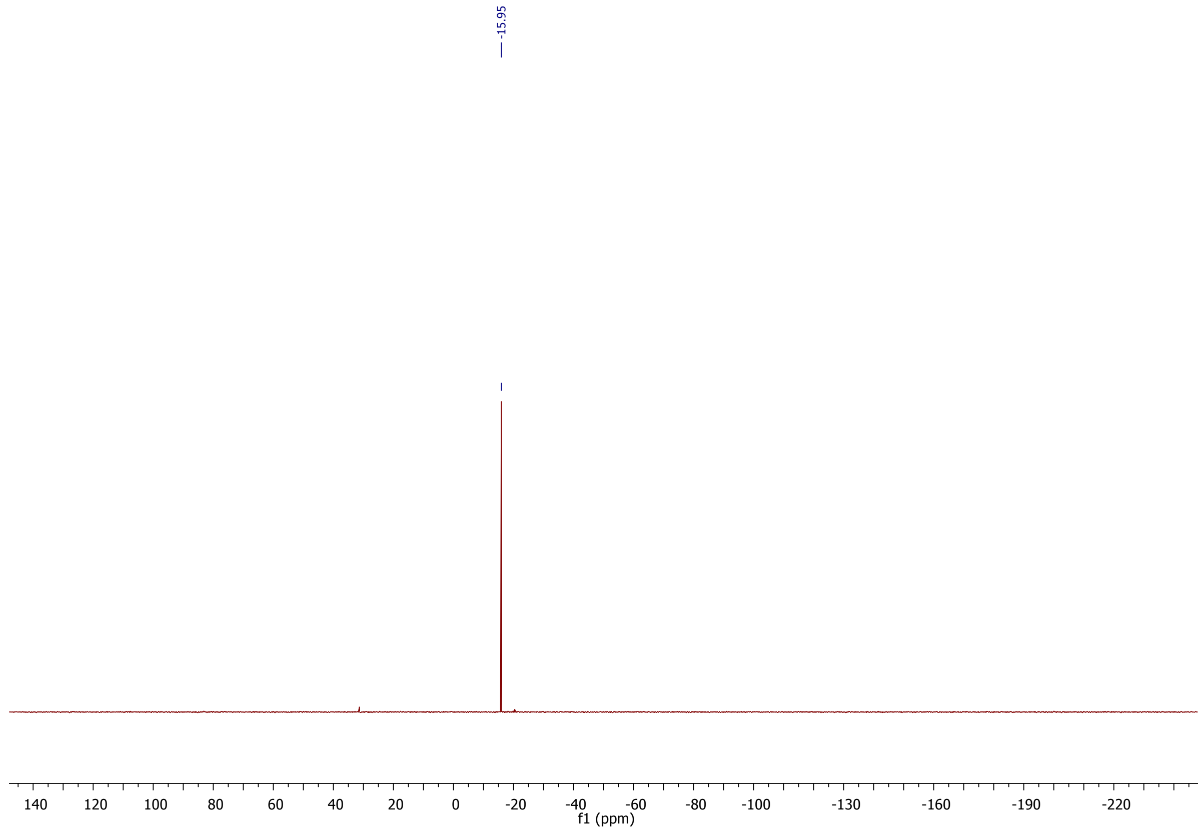

Compound 38

^{1}H NMR (500 MHz) $CDCl_3$

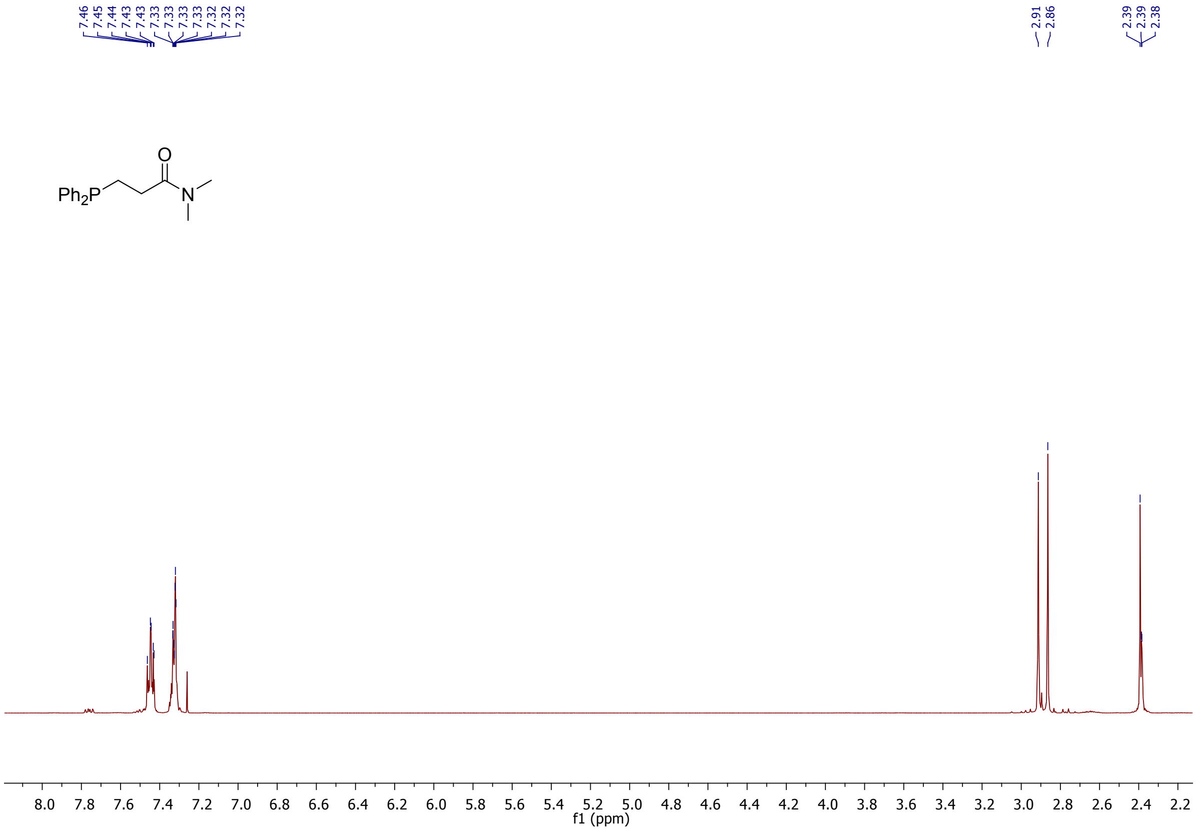

^{13}C NMR (125 MHz) $CDCl_3$

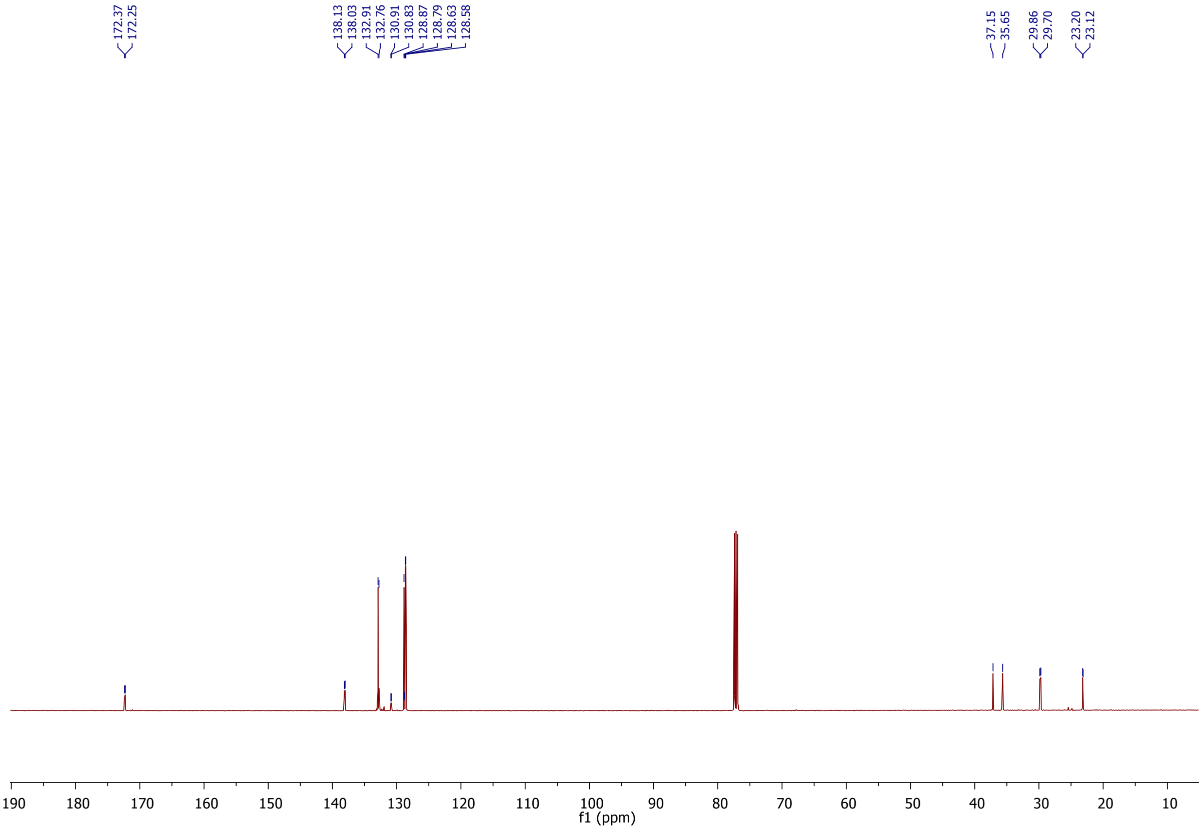

^{31}P NMR (202 MHz) $CDCl_3$

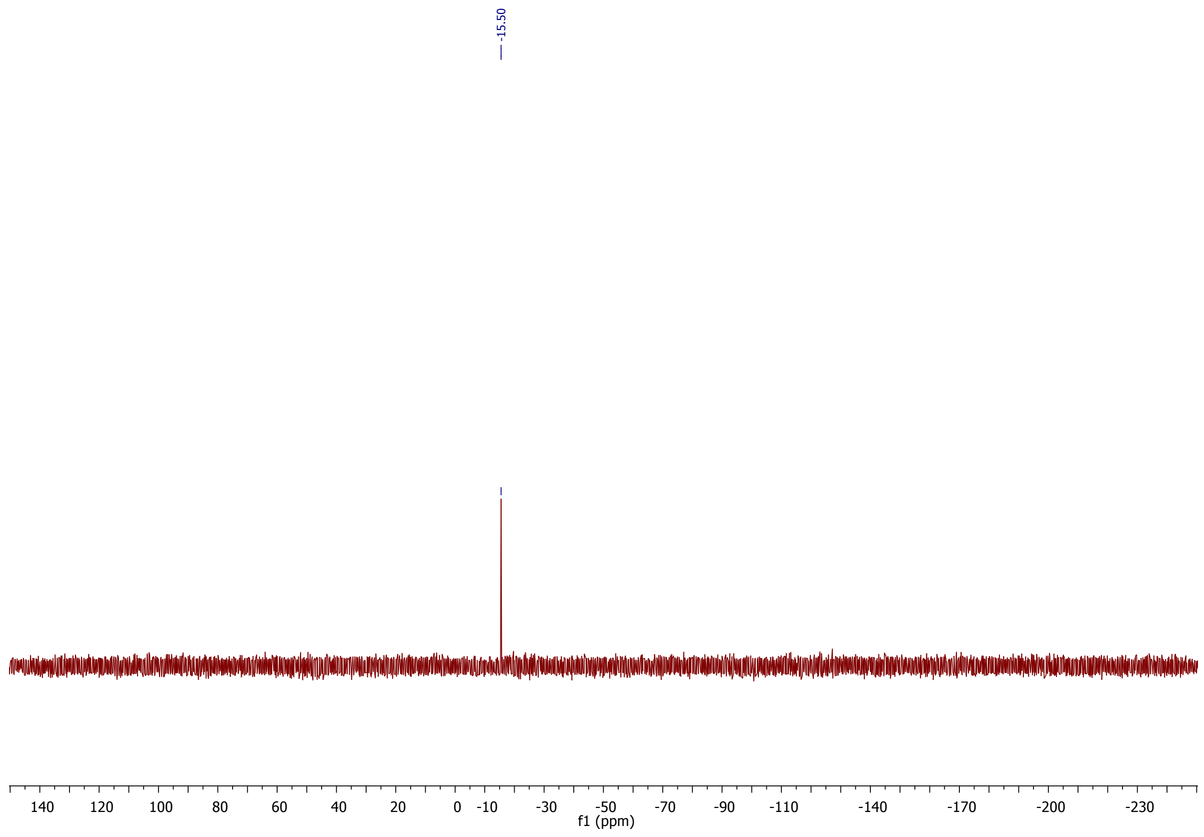

Compound 39

^{1}H NMR (500 MHz) $CDCl_3$

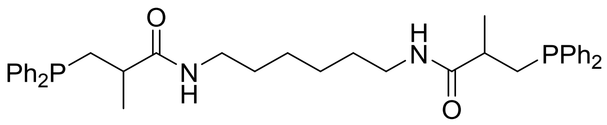

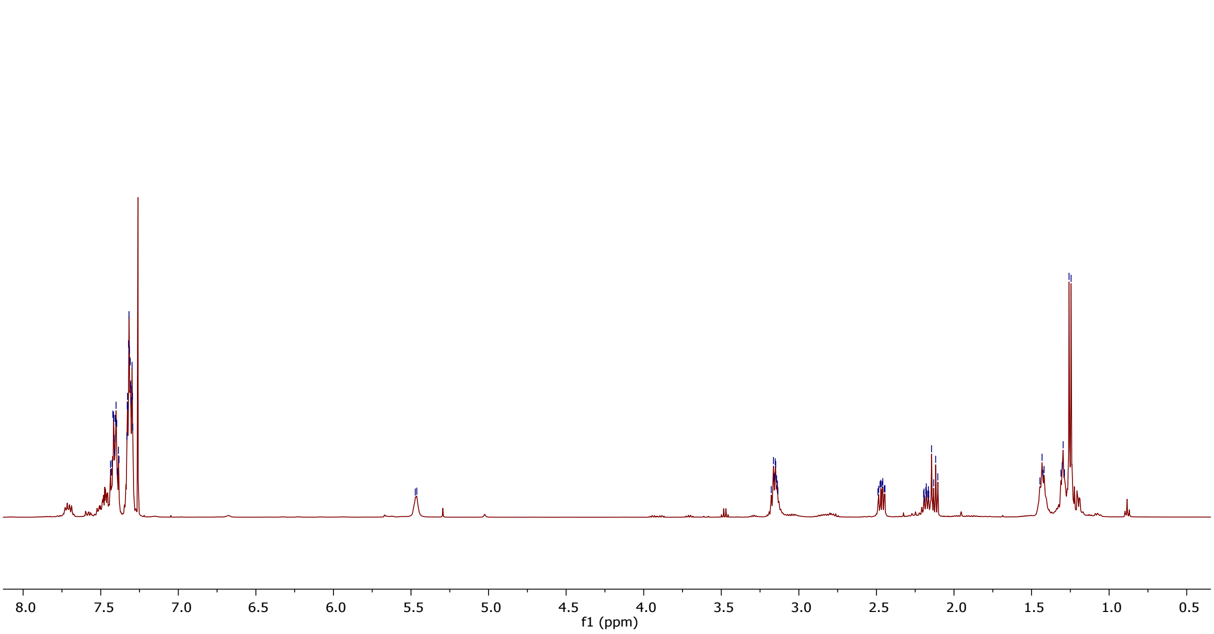

^{13}C NMR (125 MHz) $CDCl_3$

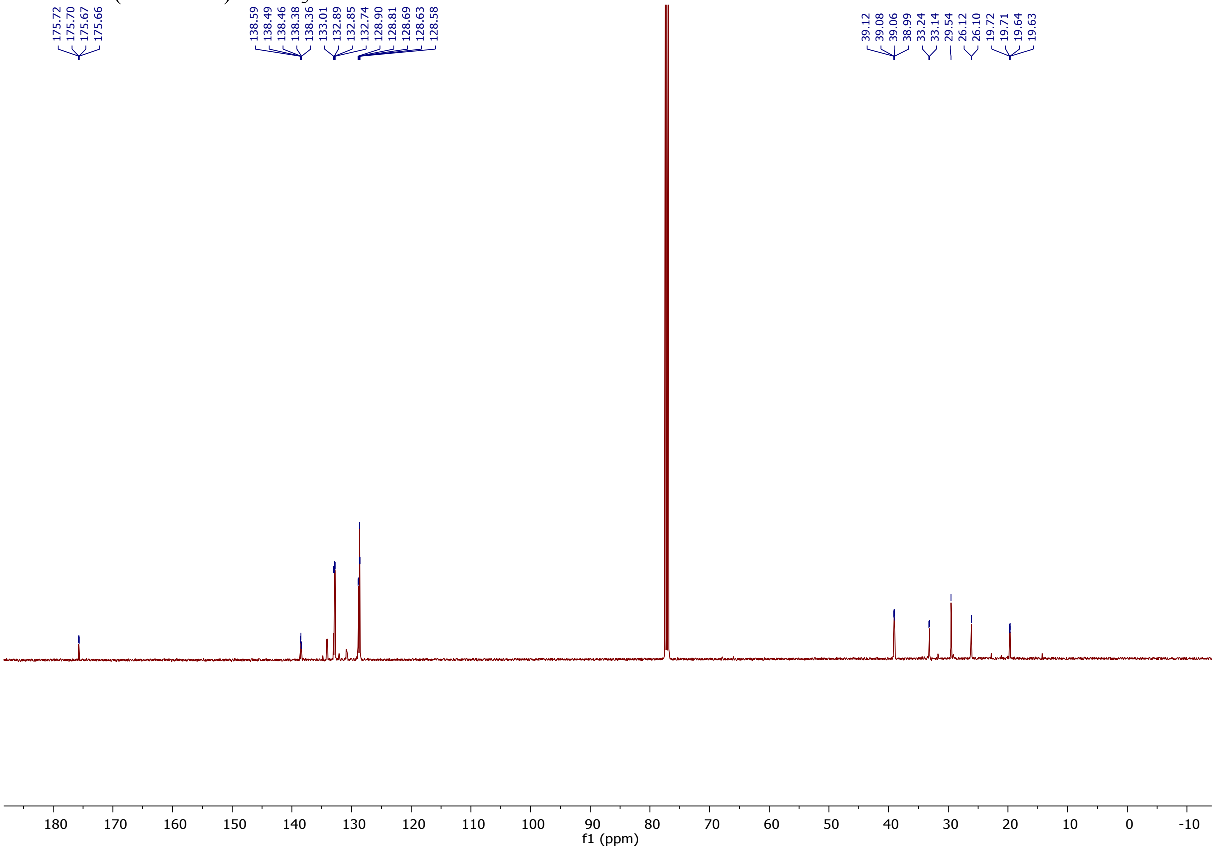

^{31}P NMR (202 MHz) $CDCl_3$

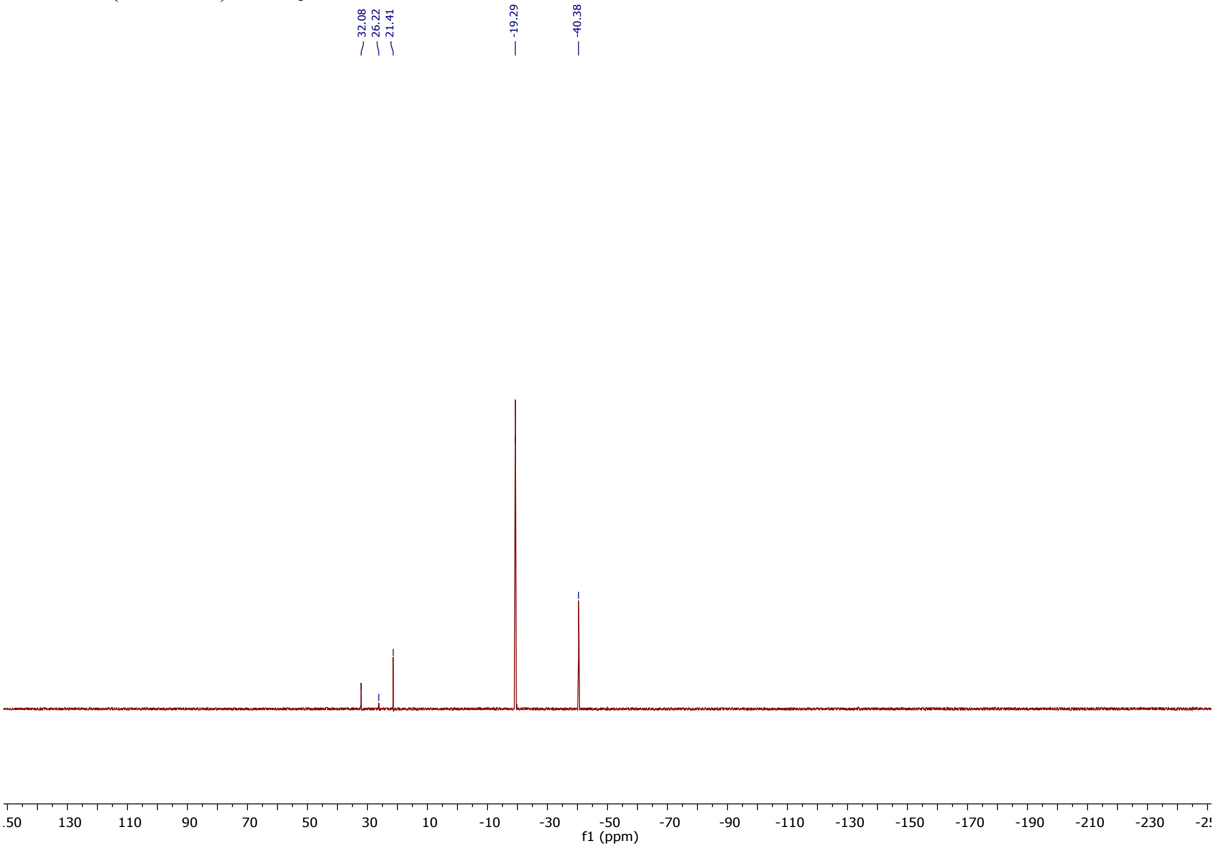

Compound 40

^{1}H NMR (500 MHz) $CDCl_3$

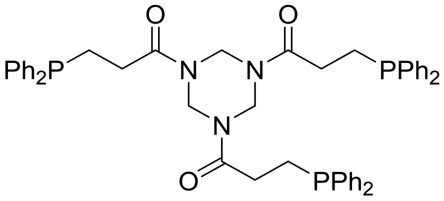

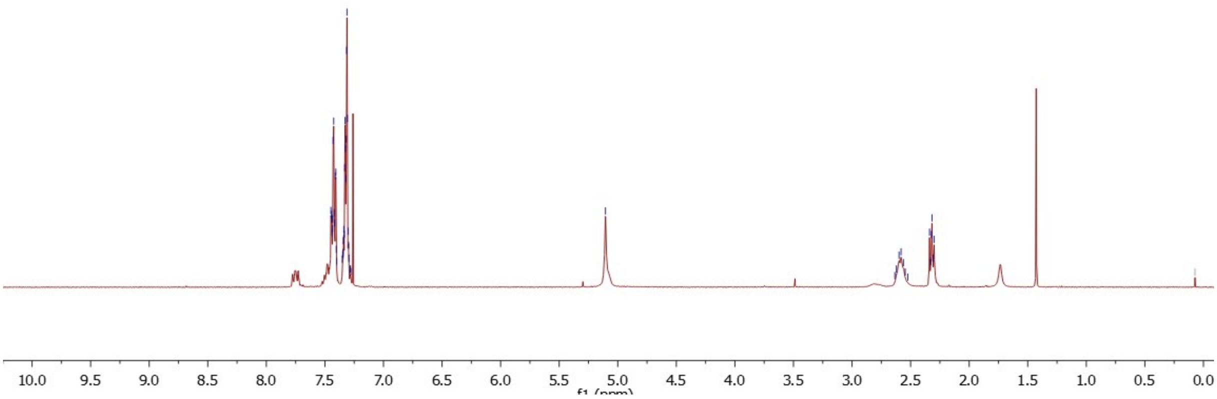

^{13}C NMR (125 MHz) $CDCl_3$

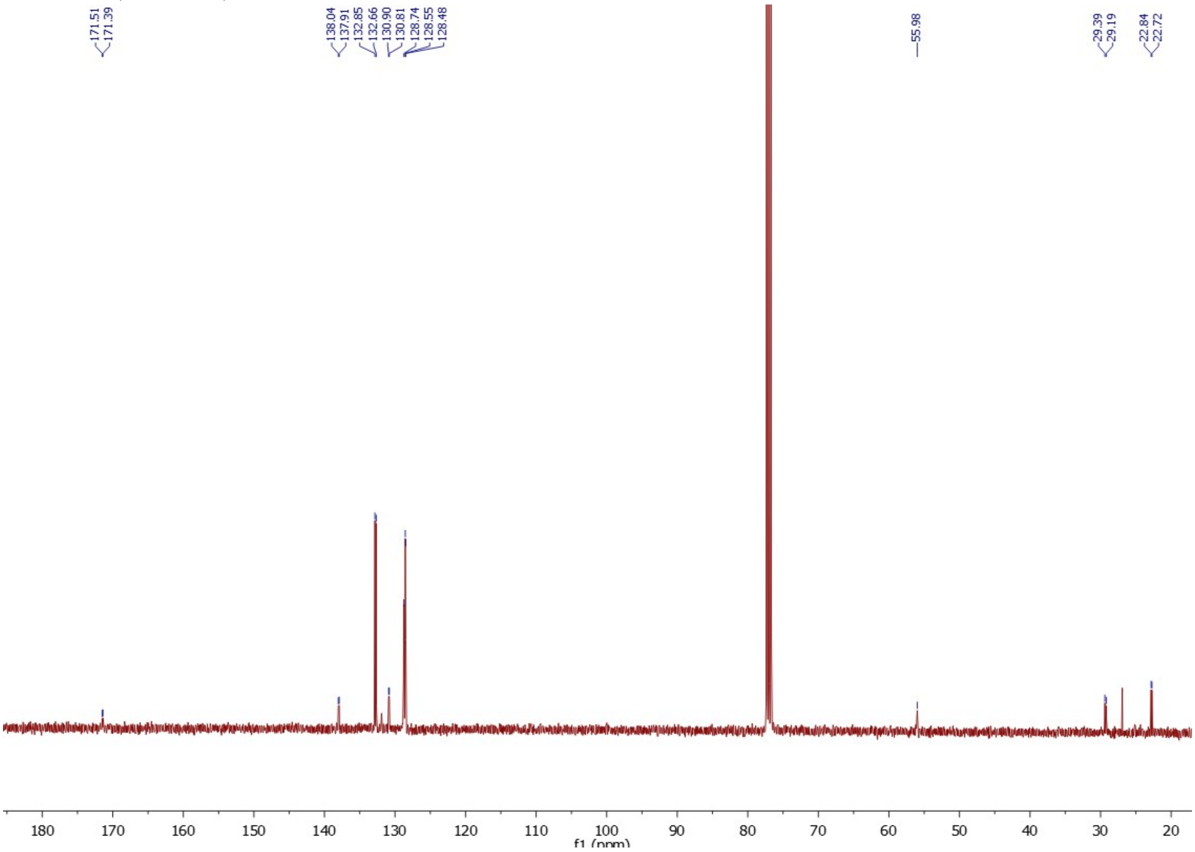

^{31}P NMR (202 MHz) $CDCl_3$

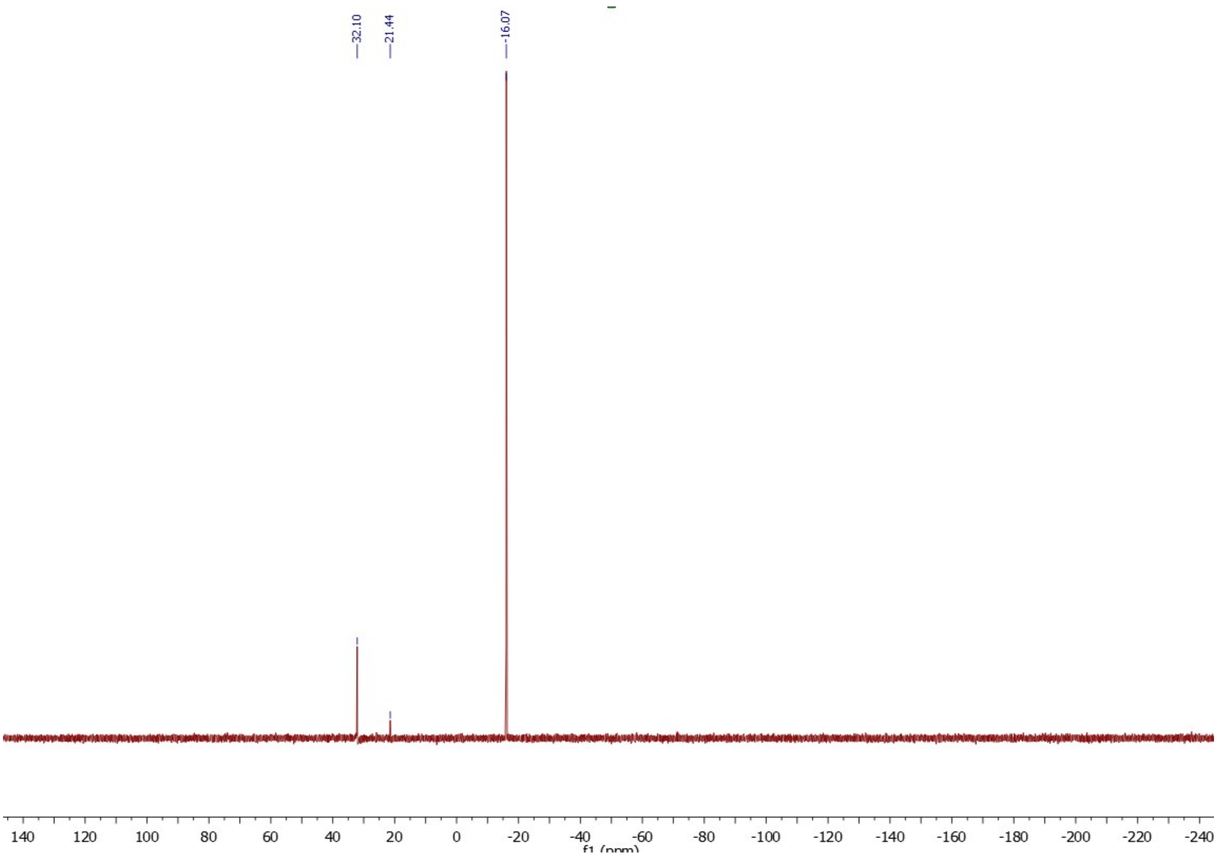

Compound 41

^{1}H NMR (500 MHz) $CDCl_3$ (reaction in presence of 2-MeTHF)

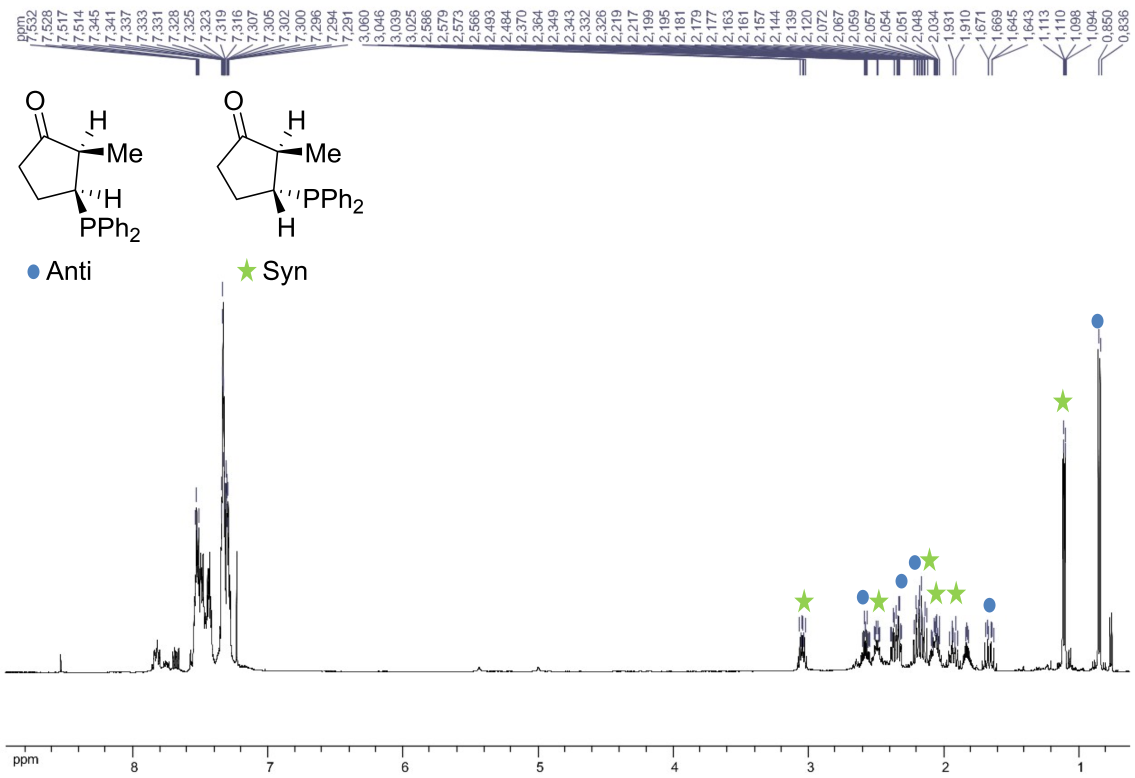

^{13}C NMR (125 MHz) $CDCl_3$ (reaction in presence of 2-MeTHF)

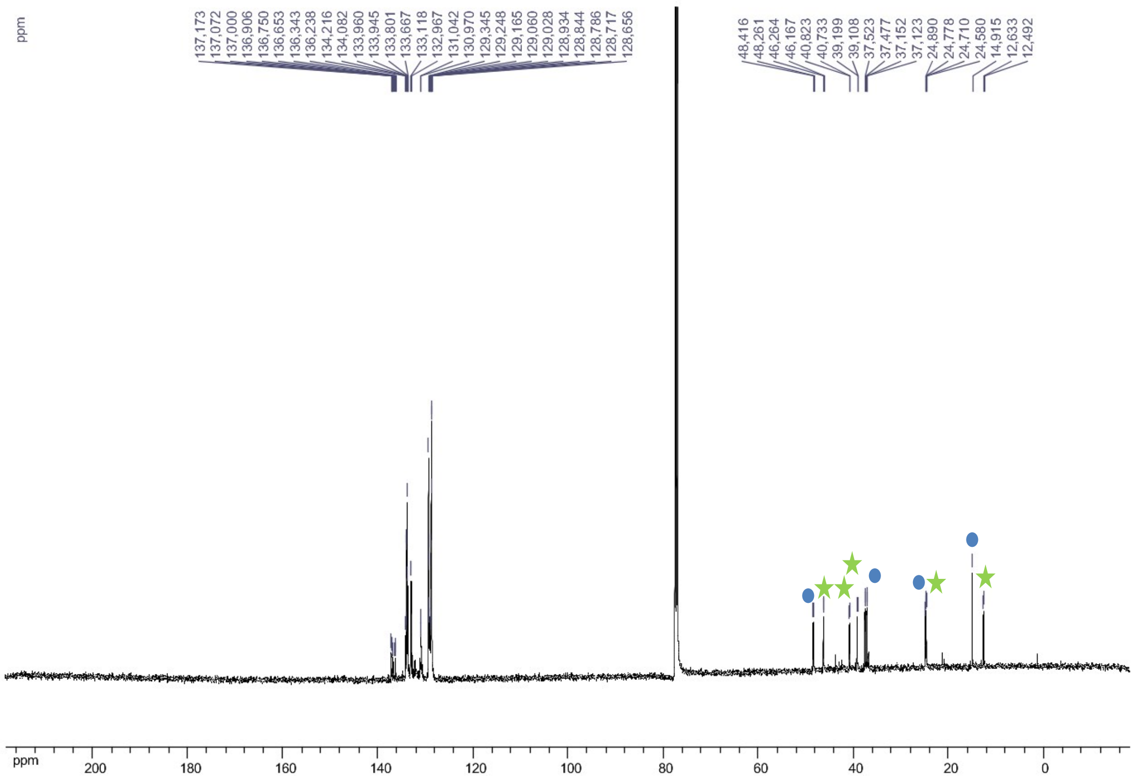

^{31}P NMR (202 MHz) $CDCl_3$ (reaction in presence of 2-MeTHF)

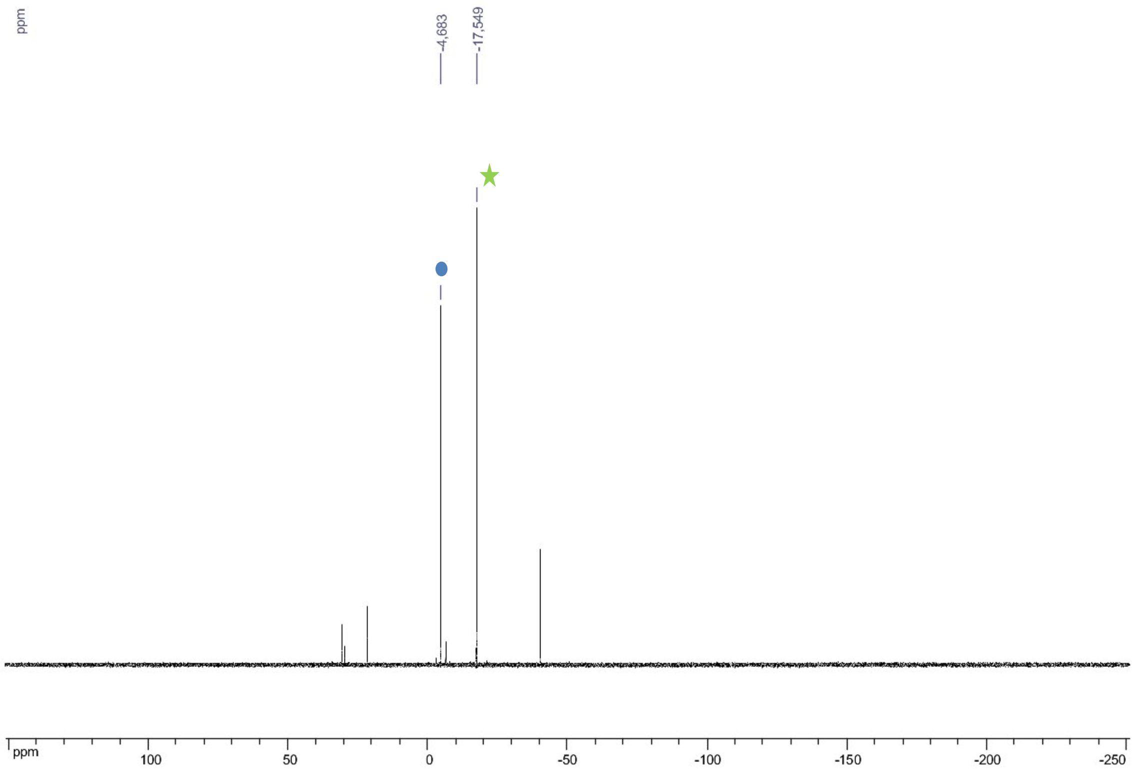

Mass spectrum from crude mixture

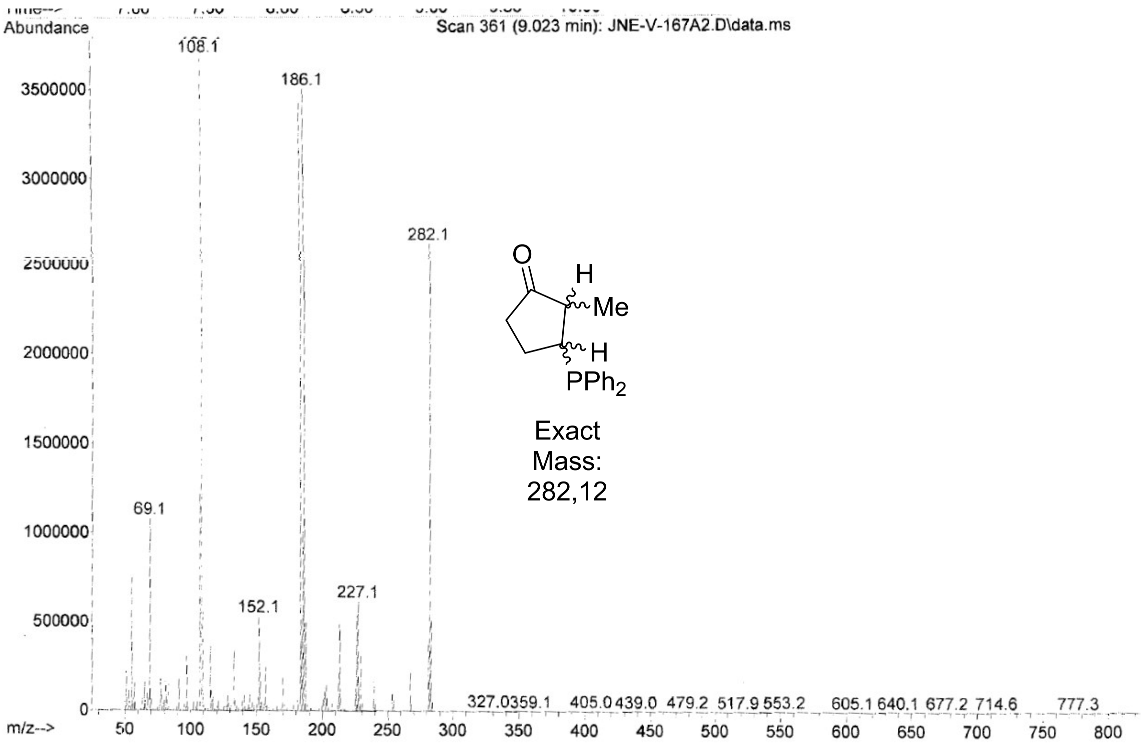

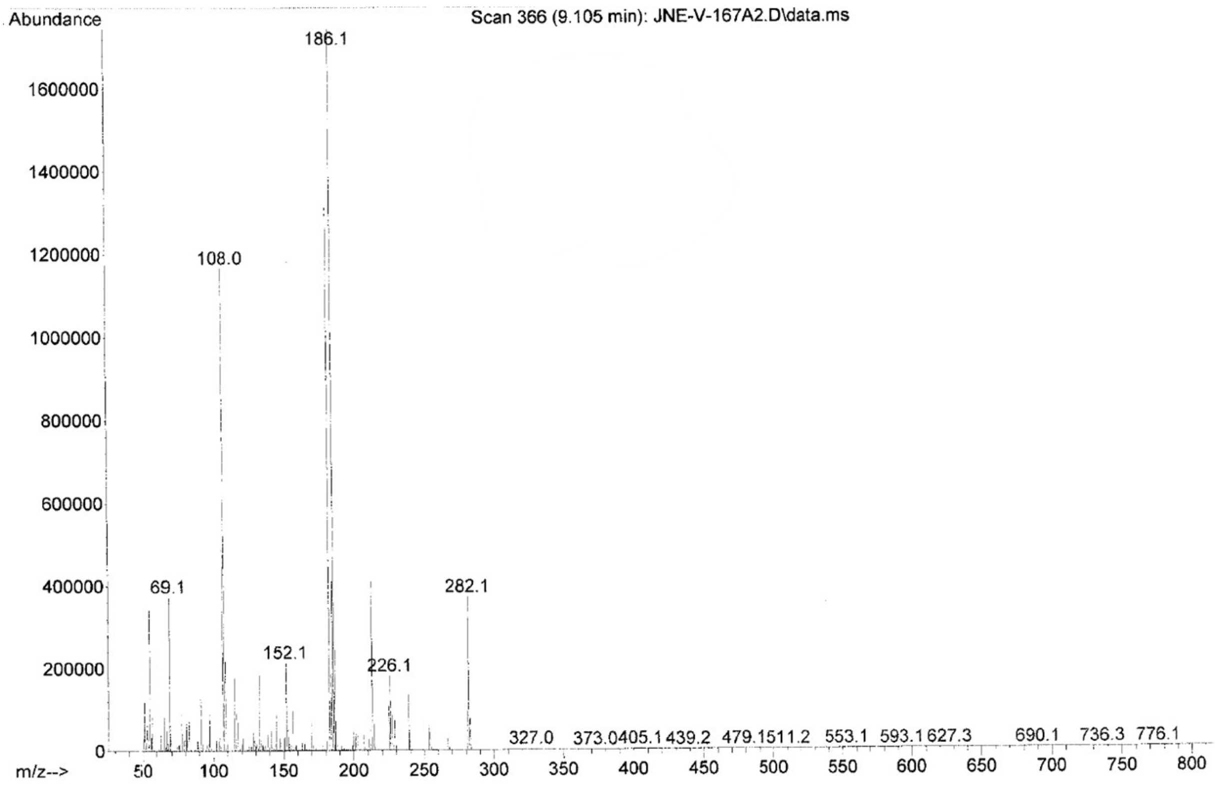

Supplement: RA-009-C9RA04896K-s001 [file RA-009-C9RA04896K-s001.pdf]
